# 3D Imaging and metabolomic profiling reveal higher neuroactive kavalactone contents in lateral roots and crown root peels of *Piper methysticum* (kava)

**DOI:** 10.1093/gigascience/giaa096

**Published:** 2020-09-22

**Authors:** Yogini S Jaiswal, Aaron M Yerke, M Caleb Bagley, Måns Ekelöf, Daniel Weber, Daniel Haddad, Anthony Fodor, David C Muddiman, Leonard L Williams

**Affiliations:** Center for Excellence in Post Harvest Technologies, North Carolina Agricultural and Technical State University, The North Carolina Research Campus, 500 Laureate Way, Kannapolis, NC 28081, USA; Department of Bioinformatics and Genomics, University of North Carolina at Charlotte, Charlotte, NC 28223, USA; Department of Chemistry, North Carolina State University, Raleigh, NC 27695, USA; Department of Chemistry, North Carolina State University, Raleigh, NC 27695, USA; Fraunhofer Development Centre X-Ray Technology EZRT, Division of Fraunhofer Institute for Integrated Circuits IIS, Department Magnetic Resonance and X-Ray Imaging MRB, Am Hubland D-97074 Wurzburg, Germany; Fraunhofer Development Centre X-Ray Technology EZRT, Division of Fraunhofer Institute for Integrated Circuits IIS, Department Magnetic Resonance and X-Ray Imaging MRB, Am Hubland D-97074 Wurzburg, Germany; Department of Bioinformatics and Genomics, University of North Carolina at Charlotte, Charlotte, NC 28223, USA; Department of Chemistry, North Carolina State University, Raleigh, NC 27695, USA; Molecular Education, Technology and Research Innovation Center (METRIC), North Carolina State University, Raleigh, NC 27695, USA; Center for Excellence in Post Harvest Technologies, North Carolina Agricultural and Technical State University, The North Carolina Research Campus, 500 Laureate Way, Kannapolis, NC 28081, USA

**Keywords:** kava, kavalactones, metabolomics, 3D imaging, mass spectrometry imaging

## Abstract

**Background:**

Kava is an important neuroactive medicinal plant. While kava has a large global consumer footprint for its clinical and recreational use, factors related to its use lack standardization and the tissue-specific metabolite profile of its neuroactive constituents is not well understood.

**Results:**

Here we characterized the metabolomic profile and spatio-temporal characteristics of tissues from the roots and stems using cross-platform metabolomics and a 3D imaging approach. Gas chromatography–mass spectrometry and liquid chromatography–mass spectrometry revealed the highest content of kavalactones in crown root peels and lateral roots. Infrared matrix-assisted laser desorption electrospray ionization (IR-MALDESI) imaging revealed a unique tissue-specific presence of each target kavalactone. X-ray micro-computed tomography analysis demonstrated that lateral roots have morphological characteristics suitable for synthesis of the highest content of kavalactones.

**Conclusions:**

These results provide mechanistic insights into the social and clinical practice of the use of only peeled roots by linking specific tissue characteristics to concentrations of neuroactive compounds.

## Introduction

*Piper methysticum* Forster f. is a plant native to the Pacific region, and its roots and products are commonly known as “kava” [1–3]. Kava is high in demand as a medicinal plant famously known for its anxiolytic, sedative, psychoactive, and calming properties when used as a recreational beverage, herbal medicine, or as a dietary supplement [2, 4, 5]. Kava is an official medicine listed in many pharmacopoeias and is used in folk medicine in the Pacific Islands [3, 5–9]. For >2 decades, kava cultivators and its market existence have continued to face the challenges of legislative and industrial disputes [4].

The bioactive neuroactive compounds from kava are called “kavalactones,” and these are predominantly present in the roots. Pathways of enzymes that affect the biosynthesis of these compounds have been identified and reported by Yang et al [10]. Novel dimeric kavalactones, namely, diyangonins (A–C), have also been reported to be isolated from kava roots [10, 11]. The kavalactone profiles genetically vary among varieties. On the basis of the chemotypes, kava varieties are classified as “noble," “medicinal," or “two-day" varieties. The Kava Act of 2002 declares noble varieties of kava to be the only legally cultivated varieties in Vanuatu, and little information is published in the literature about the non-noble varieties [12]. Irrespective of the variety, the kavalactone content can vary in different organs of the plant. Thus, it is of vital importance to establish tissue-specific chemical profiles that can aid in selection of appropriate starting raw material.

Traditionally, only peeled roots have been used for preparation of beverages [2]. However, in kava bars, the plant parts (peeled or unpeeled root or stems), the varieties (noble or adulterant non-noble type), and the concentrations used remain unregulated. The raw material sold in markets is in the form of pre-cut pieces with no identification of plant parts used. Stems and stem peels are cheap adulterants used by vendors to substitute for highly priced kava roots, and these are unsuitable for consumption. The stems contain a high pipermethystine content and are reported to be hepatotoxic, whereas the suitable plant parts (roots) do not have high pipermethystine content [13]. Thus, it is crucial to control the plant parts and varieties, which are of foremost importance among a multitude of factors that affect the resultant kavalactone content and pharmacological effects of kava [14, 15]. To our knowledge, no published studies have reported tissue-specific kavalactone contents and profiles of other secondary metabolites.

In the present study, we for the first time systematically explore the metabolites found in different parts of the kava plant. We expand on previous analytical work that used lower sensitivity instruments and did not discriminate between different tissues [2, 16–21]. We carefully control for the variety of the plant used and the tissues selected and use a combination of metabolomics and imaging technology to generate the most detailed picture to date of how metabolites differ in different specific tissues of the plant. This work represents an initial view of how social customs such as the use of the peeled roots of the plant can be linked to measurable metabolite concentrations of the neuroactive compounds.

## Results

### Mass spectrometry–based profiling reveals unique tissue-specific metabolite profiles

**Figure 1:**
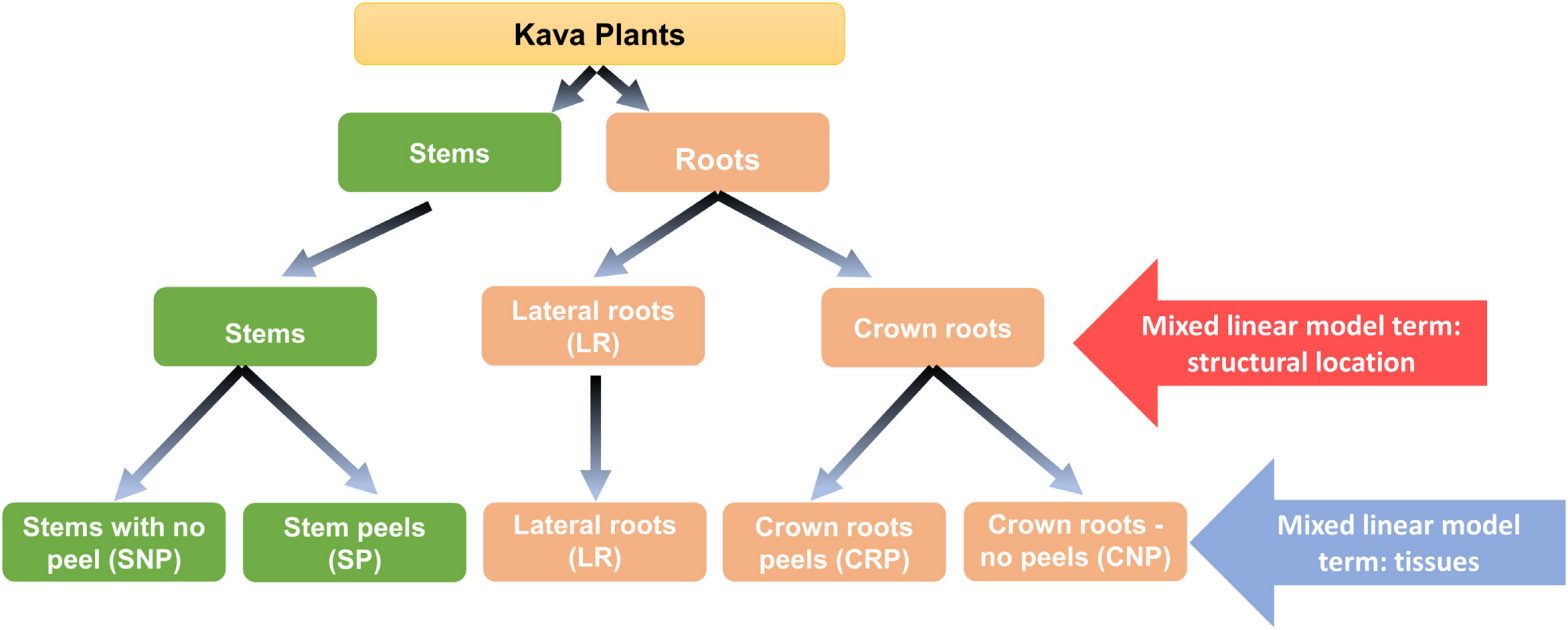
Specific kava root and stem tissues selected for the study, and the applied statistical models. Two mixed linear models were made on the basis of the selection of the various parts of *P. methysticum*. CRP,CNP, and LR denote the crown root peel, crown with no peel, and lateral roots. SP and SNP denote stem peels and stems with no peels, respectively. The plant parts are divided into stem, crown roots, and lateral roots; thus there was a model for these structural locations with levels: stem, lateral root, and crown root. Finally the stems and crown roots were divided into “peel” or “no peel” and we used these, with the lateral roots, as the levels for the model with a term for tissue (stems and crown roots “with peels” samples were excluded from the mixed linear model).

In this study, we analysed peeled and unpeeled roots and stems for their kavalactone content to establish quantitative and qualitative tissue-specific metabolite profiles. Kava stems and roots of the noble variety with >3 years of maturity were used. The tissues of the roots and stems were selected for identification of the secondary metabolites by means of liquid chromatography–mass spectrometry (LC-MS), and quantitation of kavalactones by means of gas chromatography–mass spectrometry (GC-MS) analysis for 3 separate individual plants (Fig. 1). Crown root peels (CRP), crown roots with no peels (CNP), crown roots with peels (CWP), lateral roots (LR), stem peels (SP), stems with no peels (SNP), and stems with peels (SWP) were the tissues selected for analysis. Quantitative GC-MS analysis of kavain, dihydromethysticin, and desmethoxyyangonin revealed that the highest contents of these kavalactones were found in the LR followed by crown roots and stems (LR > crown roots > stems). This pattern was observed whether or not separated tissue groups were considered individually (Table 1).

**Table 1:** Results of quantitative analysis of selected kava lactones by GC-MS in various plant parts and separated tissues of *P. methysticum*

Sample	Kavain (mg/g ± SD)	Dihydromethysticin (mg/g ± SD)	Desmethoxyyangonin (mg/g ± SD)
CWP	0.425 ± 0.235	1.689 ± 0.862	0.355 ± 0.138
LR	2.003 ± 0.615	2.842 ± 0.748	0.901 ± 0.182
SWP	0.042 ± 0.012	0.290 ± 0.110	0.080 ± 0.015
CRP	0.987 ± 0.235	1.875 ± 0.467	0.660 ± 0.146
CNP	0.922 ± 0.187	2.055 ± 0.445	0.516 ± 0.088
SP	0.069 ± 0.021	0.500 ± 0.204	0.161 ± 0.044
SNP	0.128 ± 0.027	0.356 ± 0.067	0.165 ± 0.029

CWP, LR, and SWP denote the whole crown roots, lateral roots, and stems of *P. methysticum*, respectively. CRP,CNP,SP, and SNP**denote** the crown root peel, crown root with no peel, stem peels, and stems with no peels, respectively. The concentrations of kavalactones in selected plant parts were calculated on a dry weight basis.

Different constituents had different orders of concentration in separated tissues and whole plant parts. For example, in whole roots and stems the content of dihydromethysticin was higher compared to kavain and desmethoxyyangonin. And in the separated tissues, content of dihydromethysticin was highest in CNP in contrast to kavain with highest content in CRP.

**Figure 2:**
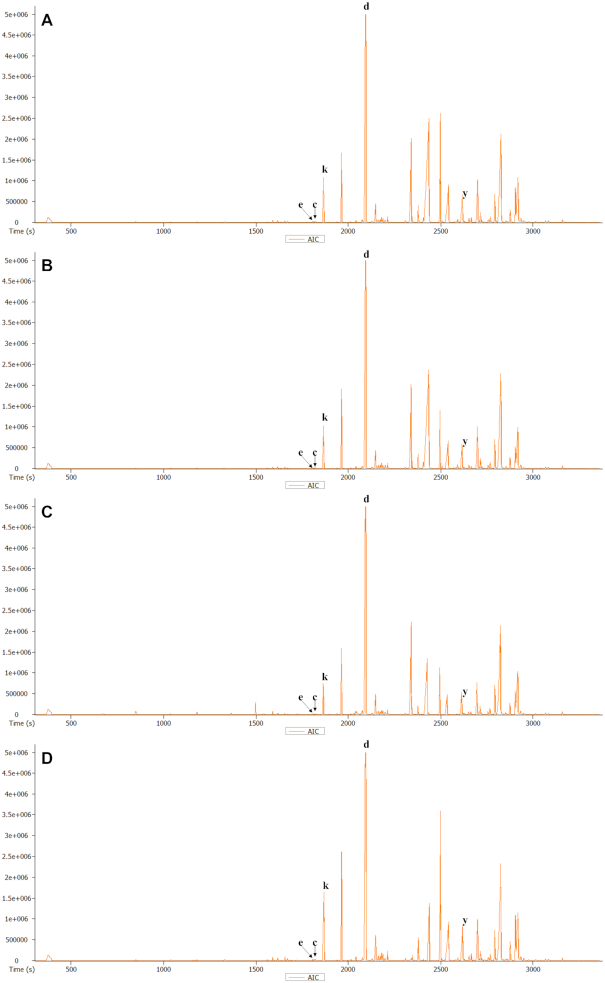
GC-MS Total Ion chromatograms of various parts of *P. methysticum* roots. **A**, peel of crown roots (CRP), **B**, crown root with no peel (CNP), **C**, crown root with peel (CWP), and **D**, lateral roots (LR). Some representative kavalactones and other compounds found in the extracts are denoted as δ-cadinol (c), dihydromethysticin (d), α-epi-7-epi-5-eudesmol (e), kavain (k), and desmethoxyyangonin (y).

In addition to quantitative analysis, we also performed untargeted metabolite profiling by means of GC-MS, which revealed the presence of 7 kavalactones, 3 dihydrochalcones, and 19 non-kava lactone compounds (Fig. 2, Supplementary Table S1, and Repository Figs R1 and R2) [22]. These profiles revealed that δ-cadinol and α-epi-7-epi-5-eudesmol are distinctly present in all tissue parts of the lateral and crown roots. Pipermethystine and benzenepropanal were found in the whole stem, and hydrocinnamic acid was found only in the SP. Overall, it was found that the crown roots and LR have a higher number of constituents compared with the stems. Except for the aforementioned differences in tissue-specific occurrence of some metabolites, all other metabolites were found in common among the crown roots, LR, and stems.

Untargeted LC-MS analysis in both positive and negative mode was also performed, and 14 kavalactones, 3 dihydrochalcones, and 19 non-kava lactone compounds were putatively identified (Supplementary Table S2, Repository Figs R3–R6)[22]. Qualitative LC-MS shows minor differences between the presence of secondary metabolites in LC-MS positive and LC-MS negative modes. In the LC-MS positive mode, there were 14 metabolites found that were not found in the LC-MS negative mode. There were 12 compounds found in LC-MS negative mode that were not seen in the LC-MS positive mode (Supplementary Table S3). Between positive and negative mode ionization of LC-MS analysis, 14 kavalactones, 3 dihydrochalcones, and 19 non-kavalactones were found in common (Supplementary Table S2). The common metabolites identified in GC-MS and LC-MS were 6 kavalactones (kavain, dihydromethysticin, dihydro-5,6-dehydrokavain, dihydrokavain, desmethoxyyangonin, and yangonin), 3 dihydrochalcones (flavokavains A–C), and 2 non-kavalactones (bornyl cinnamate and pipermethystine).

### Statistical modelling reveals that crown roots and lateral roots are similar in metabolite signatures but have a large difference from stems

#### PCA1 separates both roots from the stem samples

**Figure 3:**
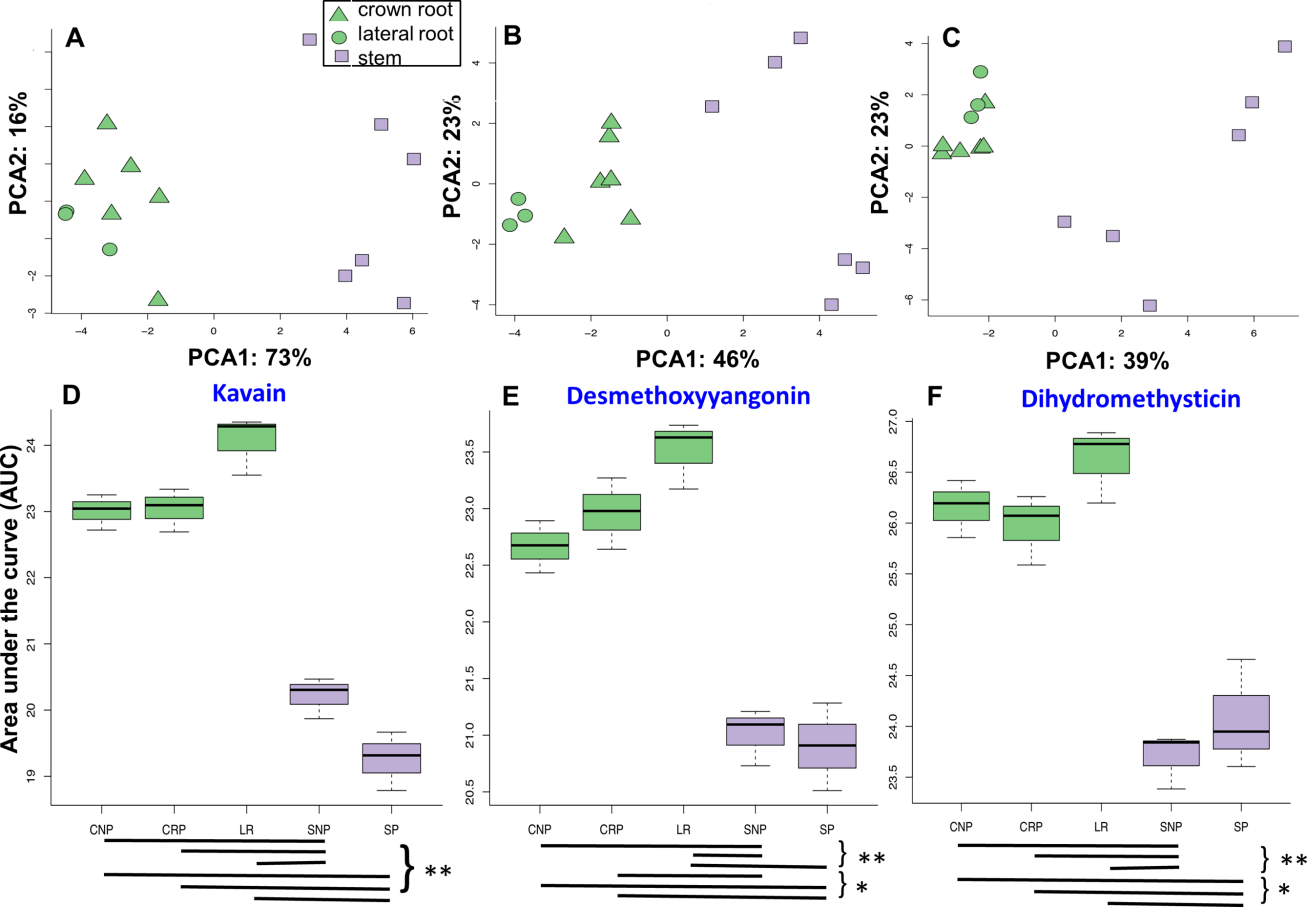
PCA and box plots for GC-MS and LC-MS analysis of kava roots and stems. PCA plots of **(A)** GC-MS, **(B)** LC-MS positive, and **(C)** LC-MS negative mode indicate a clear distinction between roots (green) and stems (purple) for each dataset. PCA1 separated the crown roots, lateral roots, and stems with adjusted *P*-values of the pairwise Student *t*-test as (3.43E−05, 0.0003458) in **(A)**, (0.000475, 0.00257) in **(B)**, and (0.000585, 0.00274) in **(C)**, respectively. PCA1 also separates both crown roots (triangles) and lateral roots (circles) from stem (squares). The mixed linear model of the adjusted*P*-values for PCA1 and PCA2 of GC-MS, LC-MS positive, and LC-MS negative mode are 2.037E−06, 0.00206, and 2.137E−05, respectively. The axes of each plot show the principal components and the percentage of the variance that they explain rounded to the nearest percent. **(D)** Kavain, **(E)** desmethoxyyangonin, and **(F)** dihydromethysticin indicated in the box plots have adjusted *P*-values of 7.05E−06, 3.40E−06, and 7.05E−06 in the mixed linear model for different tissue types. Black bars below box plots indicate statistical significance from adjusted*P*-values from pairwise Student *t*-test; *P ≤ 0.05, **P ≤ 0.01, ***P ≤ 0.001. Significant differences were observed between roots (green) and stems (purple). CRP,CNP, and LR denote the crown root peel, crown with no peel, and lateral roots, respectively. SP and SNP denote stem peels and stems with no peels, respectively. These plots exclude the whole stem and crown sections (SWP and CWP). The Δ denote the crown root , the lateral roots are denoted by Ο and the ▄ denote the stems.

We performed PCA ordination on 15 samples from 5 tissues and considered the results at the structural location (red arrow, Fig. 1) and tissue level (blue arrow, Fig. 1). At the structural location level, principal component analysis (PCA) revealed complete separation of stems from both crown roots and LR for GC-MS (Fig. 3A) and LC-MS positive (Fig. 3B) and negative mode (Fig. 3C). In addition, we observed separation between crown roots and LR for PCA1 of the LC-MS negative (Repository File R10-1), and PCA7 of the LC-MS positive datasets (Repository File R10-2)[22], although as we might expect, this separation was not as strong as the separation between the root types and stems and was not observed for all spectrometry methods.

At the tissue level, PCA1 for GC-MS differentiates CNP, CRP, and LR from SNP, whereas LC-MS negative differentiates the CNP, CRP, and LR from the SP tissues (Repository File R10-3) (Student *t*-test). PCA1 from LC-MS negative also discriminates CNP from LR (Repository File R10-4) (Student *t*-test). These results demonstrate that there are subtle differences in metabolite profiles between tissues that are detectable by our methods.

#### Mixed linear model shows that crown and lateral roots differ little from each other in metabolic profile

While the PCA data (Fig. 3A–C) gives insights into the overall structure of the dataset, it does not allow the distribution of individual metabolites to be determined. Therefore, for the data from GC-MS, which is the only quantitative spectrometry method we used, we built an initial series of linear models for each metabolite with a fixed term for structural location (with levels “crown root,” “lateral root,” and “stem”) and plant number as a random effect. Using a false discovery rate (FDR) adjusted threshold of *P* <= 0.05, the “structural locations” term showed significant associations with 21 of the 28 metabolites (Supplementary Table S4). Pairwise testing via Student *t*-test of each metabolite with a *P* < 0.05 Benjamini-Hochberg–corrected *P*-value revealed that the majority of the significant differences were between the “stem” and the 2 root types (Repository File R10-5), with only 5 of the 43 pairwise significant tests between “crown root” and “lateral roots[22].” The metabolites that were significantly different between the root types were flavokavain C, pinostrobin, hedycaryol, δ-cadinol, and bornyl cinnamate (Repository File R10-5, denoted by pound sign)[22].

These “structural locations” data give a broad picture of the metabolites in different parts of the plant. To develop a more refined understanding, we built a second series of mixed linear models with a term for tissue type (with levels “LR,” “CRP,” “CNP,” “SP,” and “SNP”) and plant as a random effect. These "tissue type" models showed significant associations for 24 of the 29 metabolites for the tissue type term (Supplementary Table S4). Of the 354 pairwise tests, 104 were significant (Repository File R10-6). However, only 6 of these significant tests were between LR and crown root tissues, and they were all CNP vs LR. In addition to squalene, the same 5 metabolites were found significant in the structural location model (Repository File R10-6, denoted by pound sign). None of the significant metabolites different between CNP and LR are kavalactones, which indicates that the LR and crown roots have very few differences in presence of kavalactones, but that they are both different from the stem tissues in terms of kavalactone profiles.

#### Pairwise analysis shows kavain, desmethoxyyangonin, and dihydromethysticin elevated in roots compared with stems

Of all the kavalactones for which we built statistical models, kavain, desmethoxyyangonin, and dihydromethysticin are especially of interest due to their well-known neurological activities amongst other kavalactones in the plant [10, 11, 23]. Consistent with the traditional use of roots in folk medicine, concentrations of all 3 of these metabolites were significantly lower in both stem tissue types than all of the root samples based on FDR-adjusted Student *t*-tests for all 29 tested metabolites (Fig. 3D–F) and quantitative GC-MS analysis (Table 1) [17]. All 3 metabolites seem to have a higher concentration in the LR than the crown roots; however, our statistical analysis was unable to significantly differentiate these metabolites in the isolated tissues.

### Lateral roots have morphological characteristics suitable for highest content of kavalactone synthesis

**Figure 4:**
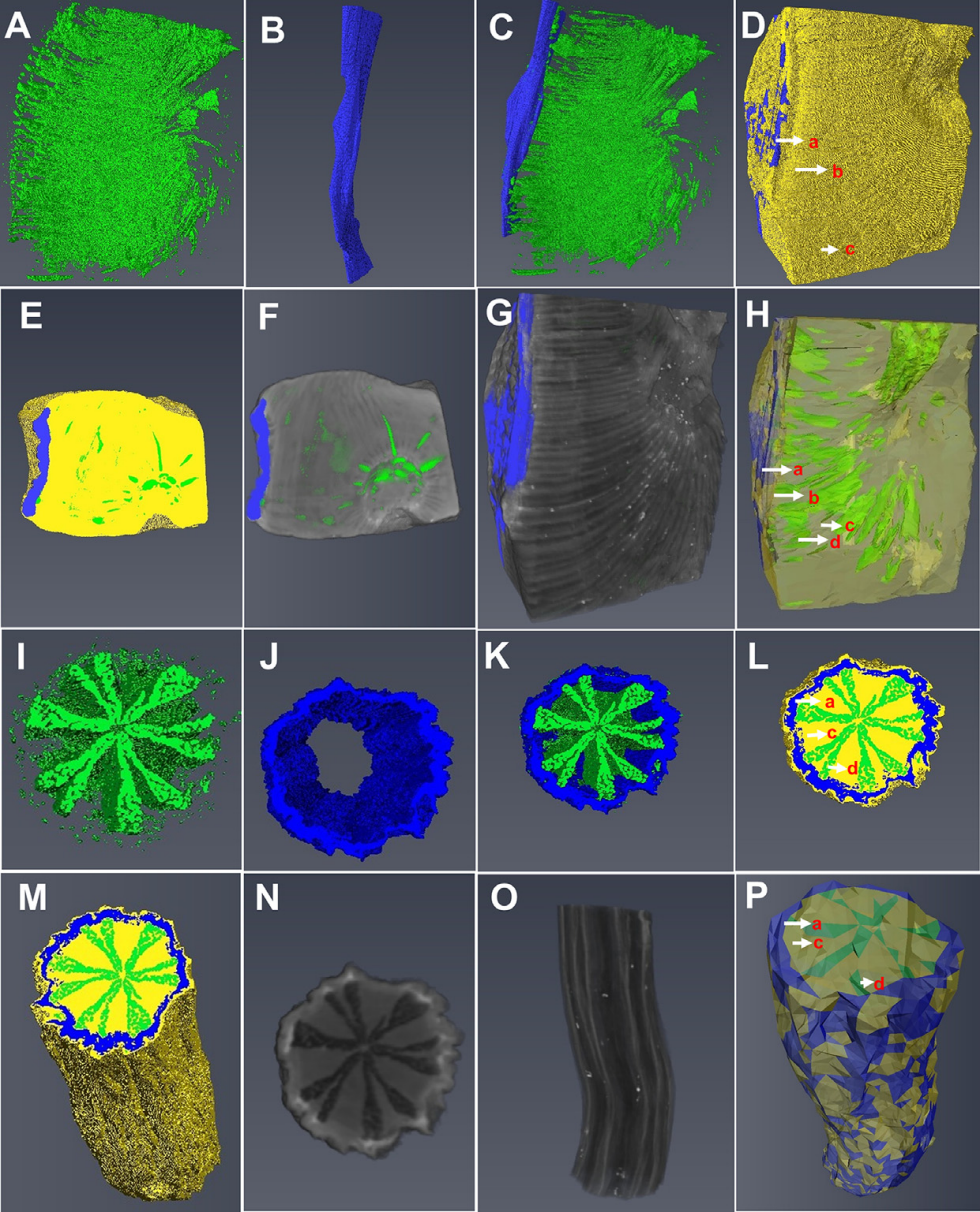
X-ray μCT images of crown and lateral roots of *P. methysticum*. **(A–H)** and **(I–P)** denote images of 3D surface reconstructions based on µCT data of crown and lateral roots, respectively. **(A)** and **(B)** are the segmented air-filled spaces in parenchyma and the cork (peel). **(C)** represents the overlay of the cork and air-filled regions in parenchyma of the crown roots. **(D)** 3D rendering image of the whole section of crown root. **(E)** and **(F)** represent the transverse view images of crown roots in 3D rendering and grey scale, respectively. **(G)** and **(H)** represent the longitudinal view images of crown roots in grey scale and 3D rendering, respectively. **(I)** and **(J)** are the segmented tissues of lateral roots, comprising air-filled spaces in parenchyma and the cork (peel), respectively. **(K)** shows an overlay of the segmented regions of lateral roots including cork and air-filled regions in parenchyma. **(L)** 3D rendering image of the whole section of lateral roots. **(M)** and **(N)** represent the transverse view of lateral roots in 3D rendering and grey scale. **(O)** and **(P)** represent the longitudinal view images of lateral roots in grey scale and 3D rendering. The sections a, b, c, and d represent cork, cortex, parenchyma, and air-filled spaces, respectively.

While kava roots serve as a major source of kavalactones, study of their morphological features remains an unexplored area. We therefore investigated the 3D topological structures of lateral and crown roots with X-ray computed microtomography (μCT) (Fig. 4, Supplementary Fig. S1, and Repository Fig. R7). The 3D images provide visualization of internal tissue structure and insights into morphology and function relationships. Owing to limited sample size (n = 2 individual plants), rigorous statistical analysis could not be applied to these images. However, to explore the differences in tissue properties that can be correlated to differences in metabolite synthesis, we calculated various geometrical descriptors on the available image datasets [24, 25]. Void shape factor and Feret diameter_max_ showed a fold difference >4.0 and >1.6, respectively, suggesting that LR and crown roots are clearly distinguishable in the morphological characteristics that affect their gas exchange properties. We found that crown roots have a higher Feret diameter_max_ and the air-filled spaces appear to be more unstructured, wide, and merged compared to LR, where the air-filled spaces are more structured and in the radial direction along the medullary rays (Supplementary Table S5 and Repository video files S1 and S2) [26, 27].

The volume of intercellular spaces, sphericity of voids, and percent porosity were comparatively higher in LR than the crown roots, indicating a more intricate and highly connected air space network (Supplementary Table S5) [28–30]. Anisotropy, which affects morphogenesis of plant organs [31], had values that were higher for the crown roots compared to LR, indicating higher morphogenesis in crown roots [32]. The void shape factor values of crown roots were found to be higher than those of LR (Table S5) and may have a correlation with the correspondingly high anisotropy values [33]. Based on the results of morphometric parameters and geometrical descriptors found in this study, we suggest that the LR exhibit characteristics for gas exchange and metabolism that can be considered to be better than those of crown roots. These findings are in agreement with the results of quantitative analysis by GC-MS, where LR were found to have the highest kavalactone content. While future work with a larger sample size will be required to determine the statistical significance of these associations, these data do suggest that LR have tissue structures for better gas exchange and metabolite synthesis compared with crown roots.

### On-tissue mass imaging reveals kavain has a higher *in situ* abundance in all tissues of crown roots

While spectrometry analyses used in this study are informative, they required disruption of tissue structures for metabolite extraction. To visualize the *in planta* distribution of kavalactones in the stems, LR, and crown roots, prior to any processing or extraction, we used Infrared matrix-assisted laser desorption electrospray ionization (IR-MALDESI) analysis.

**Figure 5:**
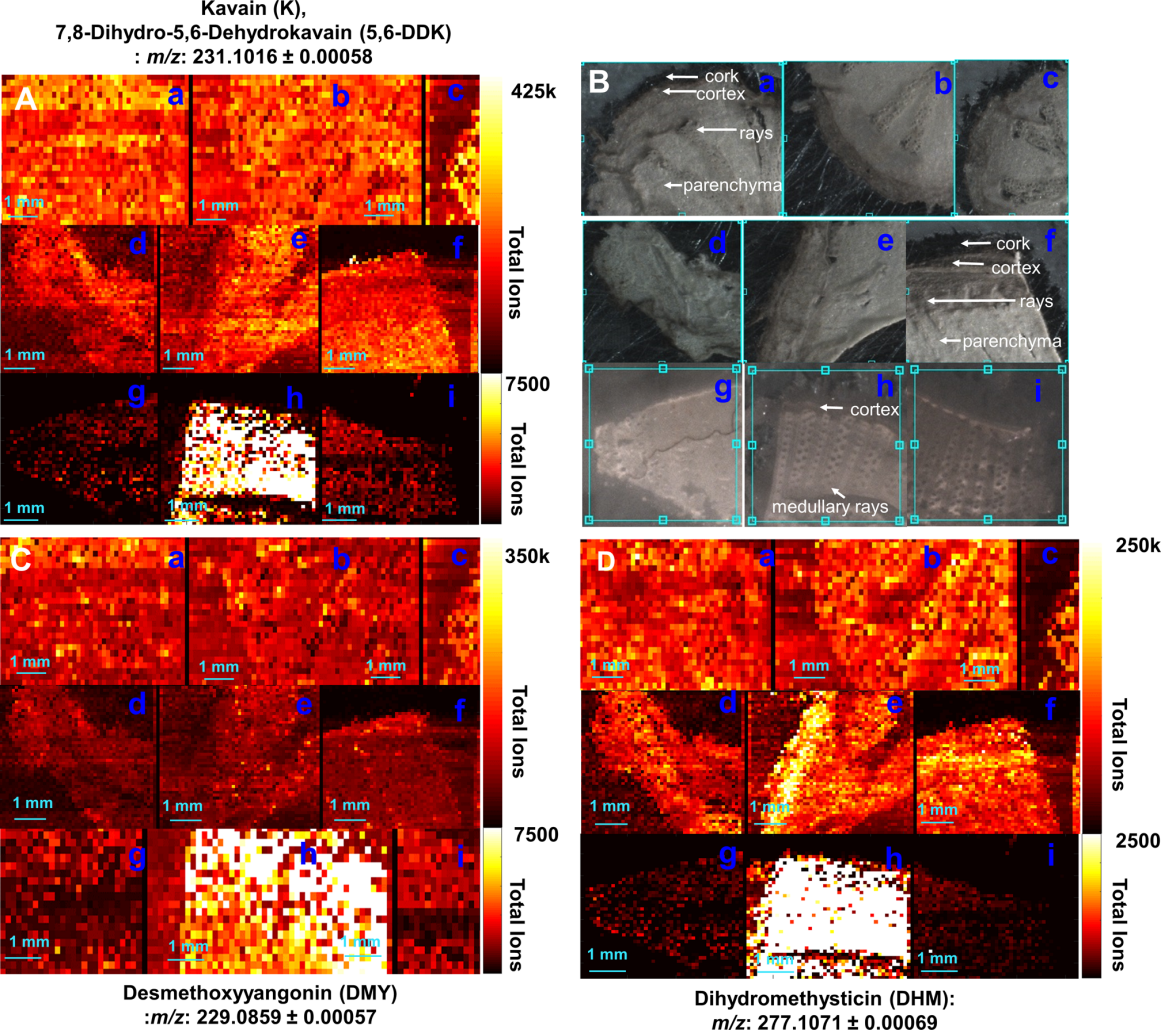
IR-MALDESI ion abundance heat maps for various constituents in different parts of *P. methysticum*. (**A**) Distribution of kavain (K), 7,8-Dihydro-5,6-dehydrokavain (5,6-DDK): *m/z* 231.1016. (**B**) Optical images of various parts. (**C**) Distribution of desmethoxyyangonin (DMY): *m/z* 229.0859. (**D**) Distribution of dihydromethysticin (DHM) *m/z* 277.107. Images in **a–c, d–f**, and **g–i** indicate lateral roots, crown roots, and stem sample images, respectively.

Of the 6 kava lactones analysed, kavain (*m/z*: 231.1016), dihydrokavain (*m/z*: 233.1172), and yangonin (*m/z*: 259.0965) had a relatively higher abundance compared with dihydromethysticin (*m/z*: 277.1071), methysticin (*m/z*: 275.0914), and desmethoxyyangonin (*m/z*: 229.0859) (Fig. 5). In the LR, kavain is found to be abundant in the parenchyma, whereas in crown roots it is found in all 3 tissues (parenchyma, cork, and cortex). Dihydromethysticin, methysticin, dihydrokavain, and yangonin were found to be the most abundant in the parenchyma, with a lower abundance in the cork and cortex region of both the types of roots (Supplementary Figs S2–S4). The stem tissues show lower abundance of all kavalactones tested except desmethoxyyangonin when compared with the lateral and crown roots. Abundance of desmethoxyyangonin was found to be uniform through all root and stem samples.

Identifications from IR-MALDESI are based on high resolution and accurate mass. However, because plant tissues have a complex secondary metabolite profile, we also validated these identifications by measuring spectral accuracy in addition to mass measurement. Isotope count heat maps generated by carbon counting based on spectral accuracy for ^12^C, ^13^C-1 revealed that the on-tissue signals for all the ions identified were highly similar to those identified in abundance heat maps, confirming the robustness of our identifications across methods (Supplementary Fig. S5) [34–36].

**Figure 6:**
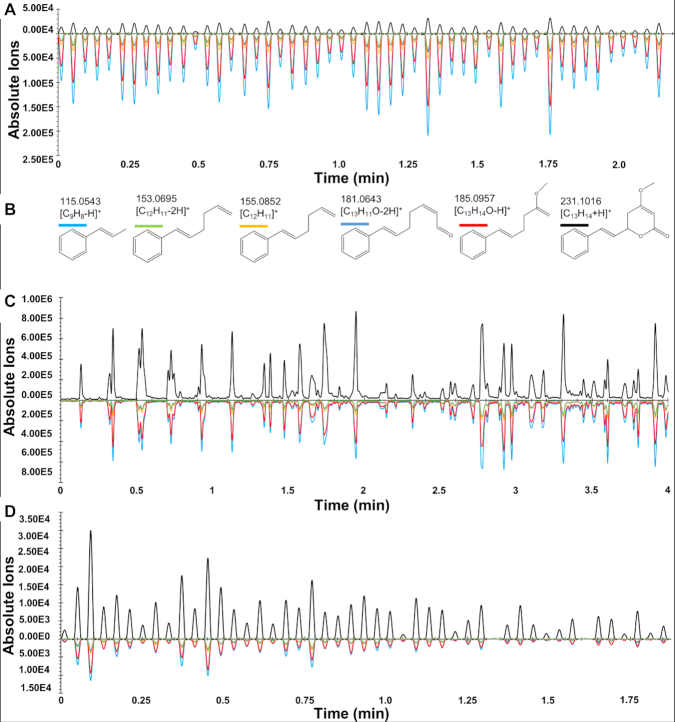
MS/MS overlay chromatograms and fragmentation pattern of kavain obtained in parallel reaction monitoring (PRM) MALDESI. **(A)** represents the overlay chromatograms of standard compound, **(B)** represents the fragmentation pattern of kavain, and **(C)** and **(D)** represent the overlay chromatograms of kavain on crown root and stem tissues, respectively. The y-axis is abundance of the precursor (top) and MS/MS fragments (bottom) of the kavain (*m*/*z*: 231.1016). The x-axis indicates time (min).

Validation of the identification of target kavalactones in plant tissues was carried out by tandem mass spectroscopy (MS/MS) fragmentation of standards, and comparison of the generated fragment ions and overlay of spectra from standards and the plant tissues (Fig. 6A and Repository Figs R8 and R9). The ratios of fragments of standard kavain matched the ratios of fragments obtained from root and stem tissues. This demonstrates that the identification of kavain in crown root and stem samples is valid and confirmed by MS/MS analysis.

## Discussion

Kava has a wide presence in the global herbal market for its calming and recreational uses, with an unregulated product range consisting of beverages, herbal drugs, and dietary supplements. There is an increasing need to validate through scientific investigation the traditional practice of using only peeled kava roots for preparation of beverages. With each kavalactone having a complex array of neurological effects (psychotic, anxiolytic, and mood stabilizing), it is also important to characterize the tissue-specific presence and contents in kava roots [37–39]. The present study represents an important initial step towards this goal.

In this study, the quantitative tissue-specific analysis by GC-MS revealed that, among the separated tissues, the CRP had the highest concentration of kavain and desmethoxyyangonin. Desmethoxyyangonin and kavain are reported to be absorbed faster than other kavalactones and cause a sudden euphoric “high” [2, 40]. The practice of using only peeled roots may avoid undesired effects of a sudden euphoric high in consumers of kava beverages made with peels. The findings of this study provide scientific evidence that agrees with the traditional practice of using only peeled roots.

The concentrations of unregulated beverages served in kava bars (*Nakamals*) are reported to be 150 times the therapeutic dose, often leading to acute intoxication, cognitive impairment, and dissociative (hallucinogenic) effects [41, 42]. The quantitative analysis in this study reveals that the lateral roots had the highest concentration of all 3 target kavalactones, kavain, dihydromethysticin, and desmethoxyyangonin.

Our study discloses the important correlation between tissue-specific secondary metabolite synthesis in kava roots, their traditional use, and the resultant pharmacological effects. We used state-of-the-art non-invasive imaging techniques, together with analysis of the unprocessed plant tissues, to overcome the drawbacks of the previously published studies by mapping *in situ* metabolite profiles of kava. By investigating metabolites biosynthesized in specific tissues of the kava plant, the study provides future avenues for harvesting medicinally important kavalactones, relevant to discovery of anti-epileptic and sedative hypnotic drugs from natural sources. The morphological and mass spectrometry–based identification of kava provides data that can aid in distinguishing adulterants and undesired plant material in raw material used for preparing kava products. The findings of this study are important for kava product manufacturers, food regulatory authorities, and consumers for safe selection of kava plant parts for product formulation and consumption. The significance of this study lies in addressing the basic but critical issues that lie in the current unregulated and unstandardized use of kava, which has become a globally widespread tranquility-promoting recreational alternative to neuroactive drugs.

## Methods

### LC-MS analysis

Roots of the noble kava variety named “Loa Leka” were collected in the last week of May 2017 from Taveuni, Fiji. The samples were >3 years of maturity and provided as gift samples from Haridaya Enterprises Ltd., Fiji. Approximately 6 kg of the root samples were collected, with 3 samples each for selected parts of the plant. At the time of harvest the stump portion was left attached to the root. Samples of roots and stems of *P. methysticum* were analysed by an Agilent 7890A GC system, coupled to an Agilent 5975C electron ionization (EI) mass selective detector (MSD) and a UPLC-QTOF MS system (Acquity UPLC-SYNAPT MS, Waters Corp., Milford, MA). For LC-MS, samples were analysed after extraction of powdered plant material in ethanol (0.5 mg/mL) with ultrasonication (Elma, Elmasonic P30H) at room temperature for 30 mins. For untargeted profiling of the prepared extracts with LC-MS analysis, a UPLC-QTOF MS system was used. It was equipped with an ACQUITY BEH UPLC C_18_ analytical column (inner diameter 1.7 μm, dimensions 2.1 × 100 mm, Waters). The analyses were performed in both positive and negative electrospray ionization (ESI) to obtain comprehensive coverage in profiling. In positive ESI mode, the mobile phase comprised 0.1% formic acid in water (solvent A) and 0.1% formic acid in acetonitrile (solvent B). In negative ESI mode, 1 mM ammonium fluoride in water (solvent A) and acetonitrile (solvent B) constituted the mobile phase. The gradient used in positive mode ESI was 0–1 min (1–15% B), 1–3 min (15–50% B), 3–8 min (50–85% B), 8–10 min (85–100% B), 10–11 min (100% B), 11–11.5 min (100–1% B), 11.5–13 min (1% B). For negative mode ESI the gradient used was 0–1 min (1–20% B), 1–3 min (20–60% B), 3–6 min (60–85% B), 6–8 min (85–100% B), 8–11 min (100% B), 11–11.5 min (100–1% B), 11.5–13 min (1% B). Leucine enkephalin was used as a lock mass standard in both positive and negative modes ([M+H]^+^ 556.2771 Da and [M-H]^−^ 554.2615 Da). An internal standard method was used for normalization. To each sample, 2 μg of para-chloro-phenylalanine was added as an internal standard, prior to analysis. The flow rate was set to 0.4 mL/min with capillary voltages of 3.2 and 3.5 in positive and negative ESI modes, respectively. The desolvation temperature was set to 350°C and the mass range used was 50–1,000 Da. The raw data files obtained from LC-MS analysis were processed using Progenesis QI software (Waters Corp., Milford, MA). The resulting dataset was organized in a matrix including sample information, labels for all the detected peaks (retention time mass pairs), and an intensity determination for each detected peak. The accurate mass information was used for identification of compounds. METLIN metabolite search and PubChem databases were used for verifying the identity of all the metabolites, by comparison of their specific molecular ions and masses.

### GC-MS analysis

For GC-MS, powdered plant material was extracted in acetone (0.25 g/mL) with the same conditions as LC-MS samples. Each of these samples were further diluted with 2 mL of acetone. All the samples were centrifuged at 12,000 rpm for 10 min (Centrifuge 5427R, Eppendorf, Enfield, CT, USA). The extracted supernatants were stored at 4°C until analysis. To each sample, 20 μL of docosanoic acid methyl ester was added as an internal standard (200 μg/mL). The samples were dried under nitrogen gas flow, derivatized with Trimethylsilyl (TMS) (Fisher Scientific, Waltham, MA, USA), and incubated at 70°C for 60 min prior to analysis.

The 3 standards kavain, dihydromethysticin, and desmethoxyyangonin were purchased from Avachem Scientific, San Antonio, TX, USA. All solvents used for analysis were of mass spectrometry grade. The quantitative and qualitative analyses were performed on 3 samples of each selected experimental group.

A DB-5MS capillary column (30 m length, 250 μm internal diameter, 0.25 μm film thickness) was used with helium as the carrier gas with a flow rate of 1 mL min^−1^. Splitless injection mode was used with oven program set to 50°C initial temperature for 1 min, and then ramped up to 280°C at the rate of 50°C for 5 min. The transfer interface temperature was set to 280°C, injection volume 1 μL, electron energy −70 V, and MS source temperature at 230°C. Scan mode was used for acquisition of characteristic ions and recording their retention times (mass range *m/z* 45–600). The raw data were processed by baseline smoothing and peak picking. Baseline smoothing, peak picking, automated and manual peak identification, and peak integration were performed using LECO ChromaTOF software (version 4.51.6.0). As part of the method development process, a data processing method to integrate specific ion masses at specific retention times was developed to quantify the data. Peak identifications were performed manually and through the automated output. All identifications were manually interrogated and corrected, as necessary. National Institute of Standards and Technology library search and PubChem databases were used for metabolite annotation. For quantitative analysis, calibration curves of standard compounds of kavain, desmethoxyyangonin, and dihydromethysticin were constructed.

For quantitative analysis using GC-MS analysis, the raw data were processed using Agilent Chemstation, exported in .aia format, and processed using LECO ChromaTOF software (4.51.6.0, Leco Corporation, St.Joseph,MI). Calibration curves of standards were constructed for quantitative analysis of the kavain, dihydromethysticin, and desmethoxyyangonin in all the selected samples. Normalization with internal standard was carried out prior to use of data for statistical analysis.

### Methods applied for development of statistical visualization models

#### Principal component analysis

The metabolite data were transformed using the R-stats function prcomp for multi-dimensional scaling (PCA) using Euclidean distance. In brief, this function produces a multi-dimensional matrix, which is then divided by axis where variance is highest. The function then returns a matrix of the 1D axis in order of highest variance to lowest. For most analyses presented, only the top 3 axes were used.

#### Mixed-effects linear model

From the nlme library in R (version 3.1–144), a mixed-effects linear model was used to investigate the fixed effects of the metadata data categories and random effects of plant number on the metabolite data (metabolite ∼ metadata + plant number) and on the results of the PCA (PCA ∼ metadata + plant number). Significance was determined with the ANOVA function in R.

#### One-way ANOVA

Using R's Base ANOVA function, a 1-way ANOVA was used to evaluate the significance of the metabolite data and the PCA data of the metabolites, as grouped by the metadata categories (tissue type, plant part, root vs stem, whole section vs part of section, only peel vs not only peel, peel present vs peel not present, and plant number). *P*-values were adjusted using the Benjamini-Hochberg method. *P* < 0.05 was arbitrarily set as the significance threshold.

#### Student t-test

The Student *t*-test was used to test pairwise comparisons using R's base t.test function. *P*-values were adjusted using the Benjamini-Hochberg method. *P* < 0.05 was arbitrarily set as the significance threshold.

### Cryo-sectioning of samples

The samples for cryo-sectioning were prepared with a modified method published in one of our previous studies [43]. Kava roots and stem samples were wrapped in non-cellulose paper moistened with ultrapure water for 12 hours at room temperature. The samples were then kept overnight under vacuo at 635 mmHg, prior to cryo-sectioning to facilitate softening of tissues by infiltration of moisture. The samples were cut into sections (∼1.5–2 cm in diameter) to enable mounting on the cryostat block. The embedding of the cut sections on cryostat blocks was accomplished by use of Surgipath Cryo-Gel matrix (Leica Microsystems, Wetzlar, Germany). Cryo-Gel is a water-soluble viscous liquid that forms a polymeric gel upon freezing [44]. Cryo-sectioning of tissues was carried out with a Leica CM1950 Cryostat (Buffalo Grove, IL, USA) at −20°C. Sections with thickness of 25 μm were prepared and carefully thaw mounted on pre-cleaned glass microscope slides.

### IR-MALDESI analysis

The slides with sections were mounted on water-cooled Peltier stage with XY motion control, housed within the custom MALDESI enclosure. The enclosure was purged with nitrogen gas until a relative humidity of <10% was reached, at which point the Peltier stage was cooled to −10°C. Allowing some time for temperature equilibration, the enclosure was opened, and the sample was exposed to ambient relative humidity. This resulted in a thin ice layer forming over the sample. The enclosure was closed again, and the relative humidity kept constant to ∼8–12% throughout analyses. A mid-IR tunable laser (IR Opolette 2371, OPOTEK, Carlsbad, CA, USA) tuned to 2.94 μm was used to fire at the tissue, resulting in desorption of neutrals from the tissue. This occurred by resonance excitation of the O—H stretching mode of water endogenously present in the sample tissues and the created ice layer. The desorbed neutrals were encountered with an orthogonal electrospray plume that ionized them in an ESI-like manner. Ions from each desorption event were synchronously analysed in a Q Exactive Plus (Thermo Fisher Scientific, Bremen, Germany) with the automatic gain control (AGC) turned off to match the pulsed nature of IR-MALDESI. Instead of AGC, a fixed injection time (IT) was used to accumulate ions resulting from the laser pulses firing at 20 Hz. Over the *m/z* range of 100–400, the achieved resolving power was 140,000 (FWHM, *m/*z = 200). The mass accuracy was parts per million (ppm), and lock mass calibrants (source 47 in polarity switching) were used for calibration.

Tissue sample ablation was performed with a 150-μm beam profile and images were captured at 100-μm step size to ensure complete tissue ablation due to oversampling. Imaging was performed at 100-μm step size, and it was found adequate for visualization of the structural features of the root and stem samples. A positive ion mode was used for analysis with 100–400 *m/z* low mass-to-charge range. Initially ions were identified by the monoisotopic, [M+H^+^]^+^*m/z* of each molecule. Followed by this step, the spectral accuracy was determined to ensure that the identified *m/z* value had the appropriate ^13^C_1_ isotope ratio for the respective naturally occurring compound. Isotope Count Heatmap compares the A+1 peak to the A peak as a certain percentage. This percentage is divided by the percentage of carbon that is naturally ^13^C (ranging between 0.96 and 1.15%). In this study a percentage of ∼1.12% was used, and each pixel was plotted by how many estimated carbons away it was from the original compound's *m/z* value. Images were then constructed with each voxel correlating to the appropriate desorption event and instrumental analysis. Carbon counting based on spectral accuracy for ^12^C, ^13^C_1_ was carried out for selected kavalactones to characterize the samples [34, 35]. The “Isotope Count Heatmap” function in MSiReader was used to plot the estimated carbons for each of the target kava lactones [45].

MS/MS analysis in parallel reaction monitoring (PRM) mode, with the same instrument settings, was used to determine the presence of kavain, desmethoxyyangonin, and dihydromethysticin. PRM mode was used for fragmentation of the precursor ion and the other fragments generated. Each generated fragment was identified using predictive software (metfrag) and compared against published reports in the literature [46, 47]. Fragments of the target compounds from tissues were compared with standard compounds and literature reports [48]. In PRM mode in Orbitrap-based Thermo MS, every nth scan is dedicated to a full MS scan across the entire *m*/*z* range of interest, or one or another of select *m*/*z* of interest. In this particular case the first scan was a full MS scan from 120 to 480 *m*/*z*, the next was an MS/MS scan of 231.1016 with an isolation window of 1.5 *m*/*z* fragmented with normalized collision energy (NCE) = 30, the third was an MS/MS scan of 277.1071 with NCE = 20, and the fourth was an MS/MS scan of 229.0859 with NCE = 35; then the cycle repeated. Each NCE value was optimized in unpublished experiments (Fig. 6).

MSiReader (v1.01k), a freely available software program developed in house specifically for mass spectrometry imaging, was used to ensure the identity of ions of interest visualized at given *m/z* [49].

### X-ray microtomography analysis

The μCT analyses were performed using a mobile compact table-top system developed at Fraunhofer Development Center X-Ray Technology EZRT (beam energy 50 kV). Two samples each, from lateral and crown root, which best represented the morphological features and were devoid of any morphological damage, were used for analysis. Two image datasets of each, the crown and LR, were used for data analysis. A reference capillary of known diameter was used to measure resolutions from the images. The samples were mounted vertically on the rotary stage and their fixation was confirmed, prior to exposure to synchrotron radiation light. Beam energy of 50 kV was used with isotropic nominal resolution of 38.1 and 35.3 μm/pixel for crown roots and 17.3 and 17.2 μm/pixel for LR, respectively. The exposure time for each sample during scanning was 400 ms. The distances between the scanner and samples for each of the crown roots were 183.4 and 169.9 mm, and for the LR the distances were 83.3 and 82.8 mm, respectively. A total of 3,200 projections were recorded with 360° rotation steps.

### Image processing of synchrotron radiation–µCT data

The postprocessing of the acquired µCT projections was performed using pyXIT software [50]. From the μCT data of crown and lateral roots, 3D surface reconstructions were rendered using the Avizo Fire software 9.3.0 (FEI, Hillsboro,OR, USA) and subsequently analysed using Avizo Fire again. We carried out 3D rendering and segmentation of various parts of the roots by applying a project protocol developed for visualization and segmentation. The protocol steps included creation of ortho slices, labels for segmentation of various root parts, resampling, surface generation, and viewing. Images were labelled and segmented into regions including the exterior, the whole root, intercellular air spaces, and the epidermis. Resampling of the labelled fields was carried out prior to generation of surface to shrink the dimensions of the grid and facilitate ease in surface generation. Surface view function was used for 3D rendering and visualization of the μCT images of the root samples. The porosity of the samples analysed was calculated by applying the ASBMR module. Geometrical descriptors of tissue structures that correlate to their gas exchange functions were calculated to identify the correlation between structure and secondary metabolite profiles of the lateral and crown roots. Feret diameters, 3D volumes, anisotropy, and so forth were calculated by applying arithmetic and label analysis module. Details of all parameters used in image analysis are provided in Repository data Fig. R7 in [22].

## Availability of Supporting Data and Materials

For statistical analysis, R studio version, 1.0.143, and R 3.5.1 (Feather Spray) were used for all computations and data manipulations. Metabolomics data have been deposited to the EMBL-EBI MetaboLights database [51] with the identifier MTBLS1485. Codes for all tests can be found at [52]. Additional repository data files are located in [22]. Snapshots of our code and other supporting data can be found in the *GigaScience* repository, GigaDB [53].

## Additional Files

**Supplementary Table S1**. Metabolites identified in roots of *P. methysticum* by GC-MS analysis

**Supplementary Table S2**. Metabolites identified in common in various parts of *P. methysticum* by LC-MS analysis

**Supplementary Table S3**. Secondary metabolites in various tissues of *P. methysticum* identified by LC/MS analysis

**Supplementary Table S4**. *P*-values of mixed linear model with GC-MS data

**Supplementary Table S5**. 3D morphological and geometric descriptors of roots of *P. methysticum*

**Supplementary Figure S1**. Pictorial representations of plant parts of *P. methysticum* and their μCT imaging and sectional views

**Supplementary Figure S2**. IR-MALDESI ion abundance heat maps for various constituents in different parts of *P. methysticum*

**Supplementary Figure S3**. Isotope Count Heatmap for kavalactones in various tissues of *P. methysticum*

giaa096_GIGA-D-20-00163_Original_Submission

giaa096_GIGA-D-20-00163_Revision_1

giaa096_Response_to_Reviewer_Comments_Original_Submission

giaa096_Reviewer_1_Report_Original_Submission Tomas Pluskal -- 7/6/2020 Reviewed

giaa096_Reviewer_2_Report_Original_Submission Purva Kulkarni, Ph.D. -- 7/20/2020 Reviewed

giaa096_Reviewer_3_Report_Original_Submission Dusan Velickovic -- 7/21/2020 Reviewed

giaa096_Supplemental_File

## Abbreviations

AGC: automatic gain control; ANOVA: analysis of variance; CNP: crown roots with no peels; CRP: crown root peels; CWP: crown roots with peels; EI: electron ionization; EMBL-EBI: European Molecular Biology Laboratory European Bioinformatics Institute; ESI: electrospray ionization; FAO: Food and Agriculture Organization; FDR: false discovery rate; FWHM: full width at half-maximum; GC-MS: gas chromatography–mass spectrometry; IR-MALDESI: infrared matrix-assisted laser desorption electrospray ionization; IT: injection time; LC-MS: liquid chromatography–mass spectrometry; LR: lateral roots; MSD: mass selective detector; MS/MS: tandem mass spectroscopy; NCE: normalized collision energy; PCA: principal component analysis; PRM: parallel reaction monitoring; pyXIT: Python X-Ray Imaging Tool; SNP: stems with no peels; SP: stem peels; SWP: stems with peels; TMS: trimethylsilyl; UPLC-QTOF MS: ultra-high performance liquid chromatography-quadrupole time-of-flight mass spectrometry; WHO: World Health Organization; μCT: X-ray micro-computed tomography.

## Competing Interests

The authors declare that they have no competing interests.

## Funding

D.W. and D.H. acknowledge financial support by the Bavarian Ministry of Economic Affairs, Regional Development and Energy. This study received financial assistance from the National Institutes of Health grants R01GM087964 and T32 Biotechnology Traineeship T32GM008776 (M.C.B).

## Authors' Contributions

Y.S.J. and L.L.W. designed and initiated the study. Y.S.J., D.W., D.H., M.C.B, and M.E. performed the research. A.F., D.C.M., D.H., and L.L.W. provided assistance and expert opinions in design, analysis, and execution of the experiments. Y.S.J., A.M.Y., M.C.B., and A.F. wrote the manuscript and analysed the data. A.M.Y. and A.F. wrote the Python and R scripts for statistical analysis of data and generating visualization plots.
