# Supplementary material for: 3D Imaging and metabolomic profiling reveal higher neuroactive kavalactone contents in lateral roots and crown root peels of Piper methysticum (kava)
Source: Gigascience. 2020 Sep 22;9(9):giaa096. doi: 10.1093/gigascience/giaa096 (PMC7507772; doi:10.1093/gigascience/giaa096)
Supplement: giaa096_GIGA-D-20-00163_Original_Submission [file giaa096_giga-d-20-00163_original_submission.pdf]

GigaScience

3D imaging and metabolomic profiling reveal higher neuroactive kavalactone contents in lateral roots and crown root peels of Piper methysticum (Kava)

--Manuscript Draft--

**Manuscript Number:**	GIGA-D-20-00163
**Full Title:**	3D imaging and metabolomic profiling reveal higher neuroactive kavalactone contents in lateral roots and crown root peels of Piper methysticum (Kava)
**Article Type:**	Research
**Funding Information:**	Bavarian Ministry of Economic Affairs, Regional Development and Energy — Dr. Daniel Haddad<br>National Institutes of Health (R01GM087964) — Dr. David C. Muddiman<br>T32 Biotechnology Traineeship — Mr. Michael C. Bagley
**Abstract:**	Kava is an important neuro-active medicinal plant. While kava has a large global consumer footprint for its clinical and recreational utilization, factors related to its use lack standardization and the tissue-specific metabolite profile of its neuroactive constituents is not well understood. Here we characterized the metabolomic profile and spatio-temporal characteristics of tissues from the roots and stems using cross-platform metabolomics and 3D imaging approach. GC-MS and LC-MS revealed the highest content of kavalactones in crown root peels and lateral roots. IR-MALDESI imaging revealed a unique tissue-specific presence of each target kavalactone. µCT analysis demonstrated that lateral roots have morphological characteristics suitable for synthesis of the highest content of kavalactones. These results provide mechanistic insights into the social and clinical practice of the use of only peeled roots by linking specific tissue characteristics to concentrations of neuroactive compounds.
**Corresponding Author:**	Leonard L. Williams<br>Center for Excellence in Post-Harvest Technologies<br>Kannapolis, NC UNITED STATES
**Corresponding Author Secondary Information:**	
**Corresponding Author's Institution:**	Center for Excellence in Post-Harvest Technologies
**Corresponding Author's Secondary Institution:**	
**First Author:**	Yogini S. Jaiswal
**First Author Secondary Information:**	
**Order of Authors:**	Yogini S. Jaiswal
	Aaron Yerke
	Michael C. Bagley
	Måns Ekelöf
	Daniel Weber
	Daniel Haddad
	Anthony Fodor
	David C. Muddiman
	Leonard L. Williams
**Order of Authors Secondary Information:**	
**Additional Information:**	
**Question**	**Response**

Powered by Editorial Manager® and ProduXion Manager® from Aries Systems Corporation

Are you submitting this manuscript to a special series or article collection?	No
**Experimental design and statistics**<br><br>Full details of the experimental design and statistical methods used should be given in the Methods section, as detailed in our Minimum Standards Reporting Checklist. Information essential to interpreting the data presented should be made available in the figure legends.<br><br>Have you included all the information requested in your manuscript?	Yes
**Resources**<br><br>A description of all resources used, including antibodies, cell lines, animals and software tools, with enough information to allow them to be uniquely identified, should be included in the Methods section. Authors are strongly encouraged to cite Research Resource Identifiers (RRIDs) for antibodies, model organisms and tools, where possible.<br><br>Have you included the information requested as detailed in our Minimum Standards Reporting Checklist?	Yes
**Availability of data and materials**<br><br>All datasets and code on which the conclusions of the paper rely must be either included in your submission or deposited in publicly available repositories (where available and ethically appropriate), referencing such data using a unique identifier in the references and in the "Availability of Data and Materials" section of your manuscript.<br><br>Have you have met the above requirement as detailed in our Minimum	Yes

Standards Reporting Checklist?	

Powered by Editorial Manager® and ProduXion Manager® from Aries Systems Corporation

Manuscript Click here to download Manuscript Manuscript.docx

3D imaging and metabolomic profiling reveal higher neuroactive kavalactone contents in lateral roots and crown root peels of *Piper methysticum* (Kava)

Yogini S. Jaiswal*[1,7], Aaron M. Yerke[2,7], Michael C. Bagley[3], Måns Ekelöf[3], Daniel Weber[4], Daniel Haddad[4,6], Anthony Fodor[2,6], David C. Muddiman[3,5,6], Leonard L. Williams*[1]

[1]*Center for Excellence in Post-Harvest Technologies, North Carolina Agricultural and Technical State University, The North Carolina Research Campus, 500 Laureate Way, Kannapolis, NC-28081, USA.* ***Emails:*** *llw@ncat.edu, yoginijaiswal@gmail.com*

[2]*Department of Bioinformatics and Genomics, University of North Carolina at Charlotte, Charlotte, North Carolina-28223, USA.* ***Emails:*** *afodor@uncc.edu, amyerke@uncc.edu*

[3]*Department of Chemistry, North Carolina State University, Raleigh, NC-27695, USA.* ***Emails:*** *mcbagley@ncsu.edu, moekeloe@ncsu.edu, dcmuddim@ncsu.edu*

[4] *Fraunhofer Development Centre X-Ray Technology EZRT, Division of Fraunhofer Institute for Integrated Circuits IIS, Department Magnetic Resonance and X-Ray Imaging MRB, Am Hubland D-97074 Wurzburg, Germany.* ***Emails:*** *Daniel.Haddad@physik.uni-wuerzburg.de, weber@mr-bavaria.de*

[5]*Molecular Education, Technology and Research Innovation Center (METRIC), North Carolina State University, Raleigh, NC-27695, USA.* ***Email:*** *dcmuddim@ncsu.edu*

[6]Co-Senior authors

[7]These authors contributed equally to this article

***Correspondence:** llw@ncat.edu, yoginijaiswal@gmail.com

Running title: Imaging and metabolomic profiling of Kava

Abstract

Kava is an important neuro-active medicinal plant. While kava has a large global consumer footprint for its clinical and recreational utilization, factors related to its use lack standardization and the tissue-specific metabolite profile of its neuroactive constituents is not well understood. Here we characterized the metabolomic profile and spatio-temporal characteristics of tissues from the roots and stems using cross-platform metabolomics and 3D imaging approach. GC-MS and LC-MS revealed the highest content of kavalactones in crown root peels and lateral roots. IR-MALDESI imaging revealed a unique tissue-specific presence of each target kavalactone. μCT analysis demonstrated that lateral roots have morphological characteristics suitable for synthesis of the highest content of kavalactones. These results provide mechanistic insights into the social and clinical practice of the use of only peeled roots by linking specific tissue characteristics to concentrations of neuroactive compounds.

Key words

Kava, kavalactones, metabolomics, 3D imaging, Mass Spectrometry imaging

Introduction

Piper methysticum Forster f. is a plant native to the Pacific region, and its roots and products are commonly known as "Kava" [1-3]. Kava is a high in demand medicinal plant famously known for its anxiolytic, sedative, psychoactive, and calming properties when used as a recreational beverage, herbal medicine, or as a dietary supplement [2, 4, 5]. Kava is an official medicine listed in many Pharmacopoeias and is used in folk medicine in the Pacific Islands [3, 5-9]. For over two decades, Kava cultivators and its market existence have continued to face the challenges of legislative and industrial disputes [4].

The bioactive neuroactive compounds from kava are the "kavalactones", and these are predominantly present in the roots. Pathways of enzymes that affect the biosynthesis of these compounds have been identified and reported by Qui et.al [10]. Novel dimeric kavalactones, namley diyangonins (A-C) have also reported to be isolated from kava roots [10-15]. The Kavalactone profiles genetically vary among varieties. Based on the chemotypes, kava varieties are classified as *noble*, *medicinal* or *Two-Day* varieties. The Kava act of 2002 declares "noble" varieties of kava as the only legally cultivated varieties in Vanuatu, and very little information in literature is known for the non-noble varieties [16]. Irrespective of the variety, the kavalactone contents can vary in different organs of the plant. Thus, it is of vital importance to establish tissue-specific chemical profiles that can aid in selection of appropriate starting raw material.

Traditionally, only peeled roots have were used for preparation of beverages [2]. However, in kava bars, the plant parts (peeled or unpeeled root or stems), the varieties (noble or adulterant non-noble type), and the concentrations used, remain unregulated. The raw material sold in

markets is in the form of pre-cut pieces with no identification of plant parts used. Stems and stem peels are cheap adulterants used by vendors to substitute highly priced kava roots, and these are unsuitable for consumption. The stems contain high content of pipermethystine and is reported to be hepatotoxic, whereas the suitable plant parts (roots) do not have high contents of pipermethystine [17]. Thus, it is crucial to control the plant parts and varieties, which are of foremost importance among a multitude of factors that affect the resultant kavalactone content, and pharmacological effects of kava [18, 19]. There have been no studies published till date, that report tissue-specific kavalactone contents and profiles of other secondary metabolites.

In this study, we for the first time systematically explore the metabolites found in different parts of the kava plant. We expand on previous analytical work which used lower sensitivity instruments and did not discriminate between different tissues [2, 20-25]. We carefully control for the variety of the plant used and the tissues selected and use a combination of metabolomics and imaging technology to generate the most detailed picture to date of how metabolites differ in different specific tissues of the plant. This work represents an initial view of how social customs such as the use of the peeled roots of the plant can be linked to measurable metabolite concentrations of the neuroactive compounds.

Results

Mass spectrometry based profiling reveals unique tissue-specific metabolite profiles

In this study, we analysed peeled and unpeeled roots and stems for their kavalactone content to establish quantitative and qualitative tissue-specific metabolite profiles. Kava stems and roots of the “noble” variety with over three years of maturity were used. The tissues of the roots and

stems were selected for identification of the secondary metabolites by LC-MS, and quantitation of kavalactones by GC-MS analysis for three separate individual plants (**Figure 1**). Crown roots peels (**CRP)**, crown roots with no peels (**CNP**), crown roots with peels (**CWP**), lateral roots (**LR**), stem peels (**SP**), stems with no peels (**SNP**) and stems with peels (**SWP**) were the tissues selected for analysis. Quantitative GC-MS analysis of kavain, dihydromethysticin and desmethoxyyangonin revealed that the highest contents of these kavalactones were found in the lateral roots followed by crown roots and stems (lateral roots > crown roots > stems). This pattern was observed whether separated tissues groups were considered individually or not (**Table 1**).

Different constituents had different orders of concentration in separated tissues and whole plant parts. For example, in whole roots and stems content of dihydromethysticin was higher compared to kavain and desmethoxyyangonin. And in the separated tissues, content of dihydromethysticin was highest in peeled crown roots in contrast to kavain with highest content in peels of crown root.

In addition to quantitative analysis, we also performed untargeted metabolite profiling by GC-MS, which revealed the presence of 7 kavalactones, 3 dihydrochalcones and 19 non-kava lactone compounds **(Figure 2**, **Supplementary Table S1,** and **Repository Figures R1** and **R2**). These profiles revealed that δ-Cadinol and α-epi-7-epi-5-Eudesmol are distinctly present in all tissue parts of the lateral and crown roots. Pipermethystine and benzenepropanal were found in the whole stem and hydrocinnamic acid was found only in the stem peels. Overall, it was found that the crown roots and lateral roots have a higher number of constituents compared to the stems.

Except for the differences in tissue specific occurrence of some metabolites discussed above, all other metabolites were found in common among the crown roots, lateral roots and stems.

Untargeted LC-MS analysis in both positive and negative mode was also performed and 14 kavalactones, 3 dihydrochalcones and 19 non-kava lactone compounds were putatively identified **(Supplementary Table S2, Repository Figures R3-R6)**. Qualitative LC-MS shows minor differences between the presence of secondary metabolites in LC-MS positive and LC-MS negative modes. In the LC-MS positive mode, there were 14 metabolites found that were not found in the LC-MS negative mode. There were 12 compounds found in LC-MS negative mode that were not seen in the LC-MS positive mode (**Supplementary Table S3**). Between positive and negative mode ionisation of LC-MS analysis, 14 kavalactones, 3 dihydrochalcones, and 19 non-kavalactones were found in common (**Supplementary Table S2**). The common metabolites identified in GC-MS and LC-MS were 6 kavalactones (kavain, dihydromethysticin, dihydro-5,6-dehydrokawain, dihydrokavain, desmethoxyyangonin and yangonin), 3 dihydrochalcones (flavokawains A-C) and 2 non-kavalactones (bornyl cinnamate and pipermethystine).

Statistical modelling reveals that crown roots and lateral roots are similar in metabolite signatures but have a large difference from stems

PCA 1 separates both roots from the stem samples

We performed PCA ordination on 15 samples from 5 tissues and considered the results at the structural location (red arrow, **Figure 1**) and tissue level (blue arrow, **Figure 1**). At the structural location level, PCA analysis revealed complete separation of stems from both crown roots and lateral roots for GC-MS (**Figure 3A**) and LC-MS positive (**Figure 3B**) and negative

mode (**Figure 3C**). In addition, we observed separation between crown roots and lateral roots for PCA1 of the LC-MS negative (**Repository File R10-1**), and PCA7 of the LC-MS positive datasets (**Repository File R10-2**), although as we might expect, this separation was not as strong as the separation between the root types and stems and was not observed for all spectrometry methods.

At the tissue level, PCA1 for GC-MS differentiates CNP, CRP, and LR from SNP, whereas LC-MS negative differentiates the CNP, CRP, and LR from the SP tissues (**Repository File R10-3**) (Student's t-test). PCA1 from LC-MS negative also discriminates CNP from LR (**Repository File R10-4**) (Student's t-test). These results demonstrate that there are subtle differences in metabolite profiles between tissues that are detectable by our methods.

Mixed linear model shows that crown and lateral roots differ little from each other in metabolic profile

While the PCA data **(Figure 3A-3C)** gives insights into the overall structure of the dataset, it does not allow for determining the distribution of individual metabolites. Therefore, for the GC-MS data, which is the only quantitative spectrometry method we used, we built an initial series of linear models for each metabolite with a fixed term for structural location (with levels "crown root", "lateral root", and "stem") and plant number as a random effect. Using a False Discovery Rate (FDR) adjusted threshold of $p <= 0.05$, the "structural locations" term showed significant associations with 21 of the 28 metabolites (**Supplementary Table S4**). Pairwise testing via Student's t-test of each metabolite with a $p < 0.05$ Benjamini-Hochberg corrected p-value revealed that the majority of the significant differences were between the "stem" and the two

root types (**Repository File R10-5**) with only 5 of the 43 pairwise significant tests between "crown root" and "lateral roots". The metabolites that were significantly different between the root types were flavokavain C, pinostrobin, hedycaryol, δ-cadinol, and bornyl cinnamate (**Repository File R10-5**, denoted by "#").

These "structural locations" data give a broad picture of the metabolites in different parts of the plant. In order to develop a more refined understanding, we built a second series of mixed linear models with a term for tissue type (with levels: "LR", "CRP", "CNP", "SP", and "SNP") and plant as a random effect. These 'tissue type" models showed significant associations for 24 of the 29 metabolites for the tissue type term (**Supplementary Table S4**). Of the 354 pairwise tests, 104 were significant (**Repository File R10-6**). However, only 6 of these significant tests were between lateral root and crown root tissues, and they were all CNP vs LR. In addition to squalene, the same 5 metabolites were found significant in the "structural location" model (**Repository File R10-6**, denoted by "#"). None of the significant metabolites different between CNP and LR are kavalactones, which indicates that the lateral roots and crown roots have very few differences in presence of kavalactones, but that they are both different from the stem tissues in terms of kavalactone profiles.

Pairwise analysis shows that kavain, desmethoxyyangonin, and dihydromethysticin are elevated in roots compared to stems

Of all the kavalactones for which we built statistical models, kavain, desmethoxyyangonin, and dihydromethysticin are especially of interest due to their well-known neurological activities amongst other kavalactones in the plant [13-15]. Consistent with the traditional use of roots in

folk medicine concentrations of all three of these metabolites were significantly lower in both stem tissue types than all of the root samples based on FDR adjusted Student's t-tests for all 29 tested metabolites (**Figure 3D-3F**) and quantitative GC-MS analysis (**Table 1**) [21]. All 3 metabolites appear to have a higher concentration in the lateral roots than the crown roots, however, our statistical analysis was unable to significantly differentiate these metabolites in the isolated tissues.

Lateral roots have morphological characteristics suitable for highest content of kavalactone synthesis

While kava roots serve as a major source of kavalactones, study of their morphological features remains an unexplored area . We therefore investigated the 3D topological structures of lateral and crown roots with X-ray computed microtomography using (μCT) **(Figure 4, Supplementary Figure S1 and Repository Figure R7)**. The 3D images provide visualization of internal tissue structure and insights into morphology and function relationship. Due to limited sample size (n=2 individual plants), application of rigorous statistical analysis was not possible for these images. However, to explore the differences in tissue properties that can be correlated to differences in metabolite synthesis, we calculated various geometrical descriptors on the available image datasets [26, 27]. Void shape factor and Feret diameter_{max} showed a fold difference greater than 4.0 and 1.6, respectively, suggesting that lateral roots and crown roots are clearly distinguishable in the morphological characteristics that affect their gas exchange properties. We found that crown roots have a higher Feret diameter_{max} and the air-filled spaces appear to be more unstructured, wide and merged compared to lateral roots where the air-filled

spaces are more structured and in the radial direction along the medullary rays **(Supplementary Table S5** and **Repository video files S1** and **S2)** [28, 29].

The volume of intercellular spaces, sphericity of voids and % porosity, were comparatively higher in lateral roots than the crown roots indicating a more intricate and highly connected air space network **(Supplementary Table S5)** [30-32]. Anisotropy, which affects morphogenesis of plant organs [33] had values that were higher for the crown roots compared to lateral roots indicating higher morphogenesis in crown roots [34]. The void shape factor values of crown roots were found to be higher than lateral roots (Table S5) and may have a correlation with the correspondingly high anisotropy values [35]. Based on the results of morphometric parameters and geometrical descriptors found in this study, we suggest that the lateral roots exhibit characteristics for gas-exchange and metabolism, that can be considered to be better than crown roots. These findings are in agreement with the results of quantitative analysis by GC-MS, where lateral roots were found to have the highest content of kavalactones. While future work with a larger sample size will be required to determine the statistical significance of these associations, these data do suggest that lateral roots have tissue structures for better gas exchange and metabolite synthesis compared to crown roots.

On-tissue mass imaging reveals kavain has a higher *in-situ* abundance in all tissues of crown roots

While spectrometry analyses used in this study are informative, they required disruption of tissue structures for metabolite extraction. In order to visualize the *in planta* distribution of

kavalactones in the stems, lateral and crown roots, prior to any processing or extraction, we used IR-MALDESI analysis.

Of the six kava lactones analysed, kavain (*m/z*: 231.1016), dihydrokavain (*m/z*: 233.1172) and yangonin (*m/z*: 259.0965) had a relatively higher abundance compared to dihydromethysticin (*m/z*: 277.1071), methysticin (*m/z*: 275.0914) and desmethoxyyangonin (*m/z*: 229.0859) (**Figure 5**). In the lateral roots, kavain is found to be abundant in the parenchyma, whereas in crown roots it is found in all three tissues (parenchyma, cork and cortex). Dihydromethysticin, methysticin, dihydrokavain and yangonin were found to be the most abundant in the parenchyma with a lower abundance in the cork and cortex region of both the types of roots (**Supplementary Figure S2-S4**). The stem tissues show lower abundance of all kavalactones tested, except desmethoxyyangonin when compared to the lateral and crown roots. Abundance of desmethoxyyangonin was found to be uniform through all roots and stems samples.

Identifications from IR-MALDESI are based on high resolution and accurate mass. However, as plant tissues have a complex secondary metabolite profile, we also validated these identifications by measuring spectral accuracy in addition to mass measurement. Isotope count heat maps generated by carbon counting based on spectral accuracy for ^{12}C, ^{13}C-1 revealed that, the on-tissue signals for all the ions identified were highly similar as the ones identified in abundance heat maps, confirming the robustness of our identifications across methods **(Supplementary Figure S5)** [36-38]**.**

Validation of the identification of target kavalactones in plant tissues was carried out by MS/MS fragmentation of standards, and comparison of the generated fragment ions and overlay of

spectra from standards and the plant tissues (**Figure 6A** and **Repository Figures R8** and **R9)**. The ratios of fragments of standard kavain, matched with the ratios of fragments obtained from root and stem tissues. This demonstrates that the identification of kavain in crown root and stem samples is valid and confirmed by MS/MS analysis.

Discussion

Kava has a wide presence in the global herbal market for its calming and recreational uses, with an unregulated product range consisting of beverages, herbal drugs and dietary supplements. There is a rising need to validate through scientific investigation the traditional practice of using 'only peeled' kava roots for preparation of beverages. With each kavalactone having a complex array of neurological effects (psychotic, anxiolytic and mood stabilizing), it is also important to characterize the tissue-specific presence and contents in kava roots [12, 39, 40]. This study represents an important initial step towards this goal.

In this study, the quantitative tissue-specific analysis by GC-MS revealed that, among the separated tissues, the crown root peels had the highest concentration of kavain and desmethoxyyangonin. Desmethoxyyangonin and kavain are reported to be absorbed faster than other kavalactones and cause a sudden euphoric "high" [2, 41]. The practice of using only peeled roots may avoid undesired effects of sudden euphoric "high" in consumers of kava beverages made with peels. The findings of this study provide scientific evidence, that agrees with the traditional practice of using only 'peeled' roots.

The concentration of unregulated beverages served in kava bars (*Nakamals*) are reported to be 150 times of the therapeutic dose, often leading to acute intoxication, cognitive impairment and

dissociative (hallucinogenic) [42, 43]. The quantitative analysis in this study reveal that, the lateral roots had the highest concentration of all three target kavalactones, kavain, dihydromethysticin and desmethoxyyangonin.

Our study discloses the important correlation between, tissue-specific secondary metabolite synthesis in kava roots, their traditional use and the resultant pharmacological effects. The state-of-the-art non-invasive imaging techniques, and analysis of the unprocessed plant tissues overcomes the drawbacks of the previously published studies by mapping *in-situ* metabolite profiles of kava. By investigation of metabolites biosynthesized in specific tissues of kava plant, the study provides future avenues for harvesting medicinally important kavalactones, in discovery of anti-epileptic and sedative hypnotic drugs from natural sources. The morphological and mass spectrometry-based identification of kava, provide data that can aid in distinguishing adulterants and undesired plant material in raw material used for preparing kava products. The findings of this study are important for kava product manufacturers, food regulatory authorities and consumers, for safe selection of kava plant parts for product formulation and consumption. The significance of this study lies in addressing the basic, but very critical issues that lie in the current unregulated and unstandardized use of kava, that has become a globally widespread tranquility recreational alternative to neuroactive drugs.

Methods

LC-MS analysis

Roots of noble kava variety named "Loa Leka" were collected in the last week of May 2017 from Taveuni, Fiji. The samples were more than three years of maturity and provided as gift samples

from Haridaya Enterprises Ltd., Fiji. About 6 kgs of the root samples were collected, with three samples each for selected parts of the plant. At the time of harvest the stump portion was left attached to the root. Samples of roots and stems of *P.methysticum* were analysed by an Agilent 7890A GC system, coupled to an Agilent 5975C electron ionization (EI) mass selective detector (MSD) and a UPLC-QTOF MS system (Acquity UPLC-SYNAPT MS, Waters Corp., Milford, MA). For liquid chromatography–mass spectrometry (LC-MS), samples were analysed after extraction of powdered plant material in ethanol (0.5 mg/ml) with ultrasonication (Elma, Elmasonic P30H) at room temperature for 30 mins. For untargeted profiling of the prepared extracts with LC-MS analysis, a UPLC-QTOF MS system was used. It was equipped with an ACQUITY BEH UPLC C_{18} analytical column (i.d. 1.7 µm, dimensions 2.1 × 100 mm, Waters, MA). The analyses were performed in both positive and negative electrospray ionization (ESI) to obtain comprehensive coverage in profiling. In positive ESI mode, the mobile phase comprised of 0.1% formic acid in water (solvent A) and 0.1% formic acid in acetonitrile (solvent B). In negative ESI mode, 1mM ammonium fluoride in water (solvent A) and acetonitrile (solvent B) constituted the mobile phase. The gradient used in positive mode ESI was: 0-1 min (1-15% B), 1-3 min (15-50% B), 3-8 min (50-85% B), 8-10 min (85-100% B), 10-11 min (100% B), 11-11.5 min (100-1% B), 11.5-13 min (1% B). For negative mode ESI the gradient used was: 0-1 min (1-20% B), 1-3 min (20-60% B), 3-6 min (60-85% B), 6-8 min (85-100% B), 8-11 min (100% B), 11-11.5 min (100-1% B), 11.5-13 min (1% B). Leucine enkephalin was used as a lock mass standard in both positive and negative modes ($[M+H]^+$ 556.2771 Da and $[M-H]^-$ 554.2615 Da). An internal standard method was used for normalisation. To each sample, 2 µg of para-chloro-phenylalaine was added as an internal standard, prior to analysis. The flow rate was set to

0.4ml/min with capillary voltages of 3.2 and 3.5 in positive and negative ESI modes, respectively. The desolvation temperature was set to 350 ºC and the mass range used was 50-1000 Da. The raw data files obtained from LC-MS analysis were processed using Progenesis QI software (Waters Corp., Milford, MA).

GC-MS analysis

For gas chromatography–mass spectrometry (GC-MS), powdered plant material were extracted in acetone (0.25g/ml) with the same conditions as LC-MS samples. Each of these samples were further diluted with 2ml of acetone. All the samples were centrifuged at 12000 rpm for 10 min (Centrifuge 5427R, Eppendorf). The extracted supernatants were stored at 4 ºC until analysis. To each sample, 20 µl of Docosanoic acid methyl ester was added as an internal standard (200 µg/ml). The samples were dried under nitrogen gas flow, derivatized with Trimethylsilyl (TMS) (Fisher Scientific, USA), and incubated at 70 ºC for 60 min prior to analysis.

The three standards kavain, dihydromethysticin and desmethoxyyangonin were purchased from Avachem Scientific, San Antonio, USA. All solvents used for analysis were of mass spectrometry grade. The quantitative and qualitative analyses were performed on three samples of each selected experimental group.

A DB-5MS capillary column (30 m length, 250 µm i.d., 0.25 µm film thickness) was used with helium as the carrier gas with a flow rate of 1 mL min^{-1}. Splitless injection mode was used with oven program set to 50 ºC initial temperature for 1 min, and then ramped up to 280 ºC at the rate of 50 ºC for 5 min. The transfer interface temperature was set to 280 ºC, injection volume 1µl, electron energy -70V and MS source temperature at 230 ºC. Scan mode was used for acquisition

of characteristic ions and recording their retention times (mass range *m/z* 45-600). The raw data was processed by baseline smoothing and peak picking. Baseline smoothing, peak picking, automated and manual peak identification and peak integration were performed using LECO ChromaTOF software (version 4.51.6.0). As part of the method development process, a data processing method to integrate specific ion masses at specific retention times was developed to quantify the data. Peak identifications were performed manually and through the automated output. All identifications were manually interrogated and corrected, as necessary. For quantitative analysis, calibration curves of standard compounds of kavain, desmethoxyyangonin and dihydromethysticin were constructed.

For quantitative analysis using GC-MS analysis, the raw data were processed using Agilent Chemstation, exported in .aia format and processed using LECO ChromaTOF software (4.51.6.0, Leco Corporation, MI). Calibration curves of standards were constructed for quantitative analysis of the kavain, dihydromethysticin and desmethoxyyangonin in all the selected samples. METLIN metabolite search and PubChem databases were used for verifying the identity of all the metabolites, by comparison of their specific molecular ions and masses. Normalisation with internal standard was carried out prior to use of data for statistical analysis.

Methods applied for development of statistical visualization models

Principal Component Analysis (PCA): The metabolite data were transformed using the R-stats function prcomp for multi-dimensional scaling (PCA) using Euclidean distance. In brief, this function produces a multi-dimensional matrix, which is then divided by axis where variance is

highest. The function then returns a matrix of the 1D axis in order of highest variance to lowest. For most analyses presented, only the top three axes were used.

Mixed Effects Linear Model: From the nlme library in R (version 3.1-144), a mixed effects linear model was used to investigate the fixed effects of the metadata data categories and random effects of plant number on the metabolite data (metabolite ~ metadata + plant number) and on the results of the PCA (PCA ~ metadata + plant number). Significance was determined with the ANOVA function in R.

One-way ANOVA: Using R's Base ANOVA function, a one-way ANOVA was used to evaluate the significance of the metabolites data and the PCA data of the metabolites, as grouped by the metadata categories (tissue type, plant part, root vs. stem, whole section vs part of section, only peel vs not only peel, peel present vs peel not present, and plant number). P-values were adjusted using the Benjamini–Hochberg method. $P < 0.05$ was arbitrarily set as the significance threshold.

Student's t-test: Student's t-test was used to test pairwise comparisons using R's base t.test function. P-values were adjusted using the Benjamini–Hochberg method. $P < 0.05$ was arbitrarily set as the significance threshold.

Cryo-sectioning of samples

Kava roots and stem samples were wrapped in non-cellulose paper and soaked with ultrapure water to facilitate softening of tissues by infiltration of moisture. The samples were then kept overnight under vacuo at 25 inHg, prior to cryo-sectioning. The samples were cut into sections (about 1.5-2 cm in diameter) to enable mounting on the cryostat block with cryogel matrix (Leica

microsystems, Germany). Cryo-sectioning of tissues was carried out with a Leica CM1950 Cryostat (Buffalo Grove, IL, USA) at -20°C. Sections with thickness of 25 μm were prepared and carefully thaw mounted on pre-cleaned glass microscope slides.

IR-MALDESI analysis

The slides with sections were mounted on water-cooled Peltier stage with XY motion control, housed within the custom MALDESI enclosure. The enclosure was purged with N_2 gas until a relative humidity of <10% was reached, at which point the Peltier stage was cooled to -10°C. Allowing some time for temperature equilibration, the enclosure was opened, and the sample was exposed to ambient relative humidity. This resulted in formation of a thin ice layer forming over the sample. The enclosure was closed again, and the relative humidity kept constant to ~8-12% throughout analyses. A mid-IR tunable laser (IR Opolette 2371, OPOTEK, Carlsbad, CA, USA) tuned to 2.94 μm was used to fire at the tissue, resulting in desorption of neutrals from the tissue. This occurred by resonance excitation of the O—H stretching mode of water endogenously present in the sample tissues and the created ice layer. The desorbed neutrals were encountered with an orthogonal electrospray plume that ionized them in an ESI-like manner. Ions from each desorption event were synchronously analysed in a Q Exactive Plus (Thermo Fisher Scientific, Bremen, Germany) with the automatic gain control (AGC) turned off to match the pulsed nature of IR-MALDESI. Instead of AGC, a fixed injection time (IT) was used to accumulate ions resulting from the laser pulses firing at 20 Hz. Over the *m/z* range of 100-400, the achieved resolving power was 140,000 (FWHM, *m/*z=200). The mass accuracy was parts per

million (ppm), and lock mass calibrants (source 47 in polarity switching) were used for calibration.

Tissue sample ablation was performed with a 150um beam profile and images were captured at 100 um step size, to ensure complete tissue ablation due to oversampling. Imaging was performed at 100 um step size, and it was found adequate for visualisation of the structural features of the roots and stems samples. A positive ion mode was used for analysis with 100-400 *m/z* low mass-to-charge range. Initially ions were identified by the monoisotopic, $[M+H+]^+$ *m/z* of each molecule. Followed by this step, the spectral accuracy (SA) was determined to ensure that the identified m/z value had the appropriate 13C1 isotope ratio, for the respective naturally occurring compound. Isotope Count Heatmap compares the A+1 peak to the A peak as a certain percentage. This percentage is divided by the percentage of carbon that is naturally 13C (ranging between 0.96-1.15%). In this study a ~1.12% was used, and each pixel was plotted by how many estimated carbons away it was from the original compound's *m/z* value. Images were then constructed with each voxel correlating to the appropriate desorption event and instrumental analysis. Carbon counting based on spectral accuracy for 12C, 13C-1 was carried out for selected kavalactones, to characterise the samples [36, 37]. The 'Isotope Count Heatmap' function in MSi reader was used to plot the estimated carbons for each of the target kava lactones [44].

Tandem-MS analysis in parallel reaction monitoring (PRM) mode with the same instrument settings, was used to determine presence of kavain (K), desmethoxyyangonin (DMY) and dihydromethysticin (DHM). Parallel Reaction Monitoring (PRM) mode was employed for fragmentation of the precursor ion and the other fragments generated. Each generated fragment

was identified using predictive software (metfrag) and compared against published reports in literature [45, 46]. Fragments of the target compounds from tissues were compared with standard compounds and literature reports [47].

MSiReader (v1.01k), a freely available software developed in house, specifically for mass spectrometry imaging, was employed to ensure the identity of ions of interest visualized at given *m/z* [48].

X-ray microtomography analysis

The µCT analyses were performed using a mobile compact table-top system, developed at Fraunhofer Development Center X-Ray Technology EZRT (beam energy 50 kV). Two samples each, from lateral and crown root which showed the best representation of morphological features and were devoid of any morphological damage were used for analysis. Two image data sets of each, the crown and lateral roots were used for data analysis. A reference capillary of known diameter was used to measure resolutions from the images. The samples were mounted vertically on the rotary stage and their fixation was confirmed, prior to exposure to SR light. Beam energy of 50 kV was used with isotropic nominal resolution of 38.1 and 35.3 µm/pixel for crown roots and 17.3 and 17.2 µm/pixel for lateral roots, respectively. The exposure time for each sample during scanning was 400 ms. The distances between the scanner and samples for each of the crown roots were 183.4 mm and 169.9 mm, and for the lateral roots the distances were 83.3 mm and 82.8 mm, respectively. A total of 3200 projections were recorded with 360° rotation steps.

Image processing of SR-µCT data

The postprocessing of the acquired μCT projections was performed using the "Python X-Ray Imaging Tool" (pyXIT) software (Maximilian Ullherr, Department of X-ray Microscopy, University of Wuerzburg, Germany, see https://www.physik.uni-wuerzburg.de/lrm/forschung/software/). From the μCT data of crown and lateral roots, 3D surface reconstructions were rendered using the Avizo Fire software 9.3.0 (FEI, Oregon, US) and subsequently analysed using Avizo Fire again. 3D rendering and segmentation of various parts of the roots were carried out by applying a project protocol developed for visualization and segmentation. The protocol steps included creation of ortho slices, labels for segmentation of various root parts, resampling, surface generation and viewing. Images were labelled and segmented into regions including the exterior, the whole root, intercellular air spaces and the epidermis. Resampling of the labelled fields was carried out prior to generation of surface, to shrink the dimensions of the grid and facilitate ease in surface generation. Surface view function was used for 3D rendering and visualization of the μCT images of the root samples. The porosity of the samples analysed was calculated by applying the ASBMR module. Geometrical descriptors of tissue structures that correlate to their gas-exchange functions were calculated, to identify the correlation between structure and secondary metabolite profiles of the lateral and crown roots. Feret's diameters, 3D volumes, anisotropy etc. were calculated by applying arithmetic and label analysis module. Details of all parameters used in image analysis are provided in **Repository data Figure R7** in https://github.com/palomnyk/kava_3D_imaging_and_metabolomics.

Availability of supporting data and materials

For statistical analysis, R studio version, 1.0.143 and R 3.5.1 (Feather Spray) were used for all computations and data manipulations. Codes for all tests can be found at https://github.com/palomnyk/root_stem_crown_comparison. Additional repository data files are located in https://github.com/palomnyk/kava_3D_imaging_and_metabolomics

Additional files

Supplementary Table S1. Metabolites identified in roots of *P.methysticum* by GC-MS analysis.

Supplementary Table S2. Metabolites identified in common in various parts of *P.methysticum* by LC-MS analysis.

Supplementary Table S3. Secondary metabolites in various tissues of *P.methysticum* identified by LC/MS analysis

Supplementary Table S4. P-values of mixed linear model with GC/MS data

Supplementary Table S5. 3D morphological and geometric descriptors of roots of *P. methysticum.*

Supplementary Figure S1. Pictorial representations of plant parts of *P.methysticum* and their μCT imaging and sectional views.

Supplementary Figure S2. IR-MALDESI ion abundance heatmaps for various constituents in different parts of *P. methysticum*.

Supplementary Figure S3. Isotope Count Heatmap for kavalactones on various tissues of *P.methysticum*.

Abbreviations

3D: three dimensional; GC-MS: gas chromatography-mass spectrometry; LC-MS: Liquid chromatography-mass spectrometry; IR-MALDESI: infrared matrix-assisted laser desorption electrospray ionization; μCT: X-ray micro-computed tomography; GAD: generalized anxiety disorder; WHO: world health organization; FAO: food and agriculture organization; CRP: crown roots peels; CNP: crown roots with no peels; CWP: crown roots with peels; LR: lateral roots; SP: stem peels; SNP: stems with no peels; SWP: stems with peels; PCA: principal component analysis; FDR: false discovery rate; EI: electron ionization; MSD: mass selective detector; UPLC-QTOF MS: ultra-high performance liquid chromatography-quadrupole time-of-flight mass spectrometry; ESI: electrospray ionization; TMS: trimethylsilyl; 1D: one dimensional; ANOVA: analysis of variance; AGC: automatic gain control; IT: injection time; K: kavain; DMY: desmethoxyyangonin; DHM: dihydromethysticin; PRM: parallel reaction monitoring; pyXIT: python X-Ray Imaging tool

Consent for publication

Not applicable

Funding

D.W. and D.H. acknowledge financial support by the Bavarian Ministry of Economic Affairs, Regional Development and Energy. This study received financial assistance from the National

Institutes of Health grants R01GM087964 and T32 Biotechnology Traineeship T32GM008776 (M.C.B).

Author contributions

Y.S.J. and L.L.W designed and initiated the study. Y.S.J., D.W., D.H., M.C.B, M.E., performed the research. A.F., D.C.M., D.H. and L.L.W. provided assistance and expert opinions in design, analysis and execution of the experiments. Y.S.J., A.M.Y., M.C.B., and A.F., wrote the manuscript and analysed the data. A.M.Y. and A.F., wrote the Python and R scripts for statistical analysis of data and generating visualization plots.

Acknowledgments

The Center for Excellence in Post-Harvest Technologies appreciates the support of Mr. Abhishek Sapra of Haridaya Enterprises Ltd., Fiji, for the in-kind donation of kava samples and arranging the logistics for plant sample collection. No conflict of interest declared.

References

1. WHO. Piperis methystici rhizoma. WHO Monographs on Selected Medicinal Plants. Geneva: World Health Organization, 2002;2:231.

2. Lebot V. An overview of kava production in the Pacific Islands: what we do know and what we don’t know. J. South Pacific Agric. 1997;4:55-62.

3. Purdel C. Assessment report on Piper methysticum G. Forst., rhizoma. Committee on Herbal Medicinal Products (HMPC), London, UK, 2017.

4. FSANZ. Kava A Human Health Risk Assessment. Wellington, New Zealand: Food Standards Australia New Zealand, 2004:1-26.

5. Singh YN. Kava: an overview. J. Ethnopharmacol. 1992;37:13-45.

6. SPC. Pacific Kava-A producer’s guide: Secretariat of the Pacific Community (SPC), Hawaiian Kava Center, Fiji Islands 2001.

7. Goldberg AB, Mark. Herbal Medicine. Austin, TX: American Botanical Council; 2000.

8. BPC. Kavae Rhizoma. British Pharmaceutical Codex: Authority of the Council of the Pharmaceutical Society of Great Britain; 1907. p. 566.

9. Blumenthal M, Busse WR. The complete German Commission E monographs : therapeutic guide to herbal medicines. In: Blumenthal M, editor. Boston, MA: American Botanical Council 1998;xxii:685.

10. Lehmann E, Kinzler, E., and Friedemann, J. Efficacy of a special Kava extract (Piper methysticum) in patients with states of anxiety, tension and excitedness of non-mental origin - A double-blind placebo-controlled study of four weeks treatment. Phytomedicine. 1996;3:113-9.

11. Volz HP, Kieser M. Kava-kava extract WS 1490 versus placebo in anxiety disorders-a randomized placebo-controlled 25-week outpatient trial. Pharmacopsychiatry. 1997;30:1-5.

12. Baum SS, Hill R, Rommelspacher H. Effect of kava extract and individual kavapyrones on neurotransmitter levels in the nucleus accumbens of rats. Prog. Neuro-Psychopharmacol. Biolog. Psychiatry. 1998;22:1105-20.

13. Pluskal T, Torrens-Spence MP, Fallon TR, De Abreu A, Shi CH, Weng JK. The biosynthetic origin of psychoactive kavalactones in kava. Nat Plants. 2019;5:867-78.

14. Yang JX. A New Kavalactone Dimer from Piper methysticum. Chemistry of Natural Compounds. 2019;55:606-9.

15. Yuan Y, Yang JX, Nie LH, Li BL, Qin XB, Wu JW, Qiu SX. Three new kavalactone dimers from Piper methysticum (kava). J Asian Nat Prod Res. 2018;20:837-43.

16. FAO. Kava Act 2002: An Act to regulate the cultivation, sale and export of kava and kava products. Vanuatu Legislation: Republic of Vanuatu, 2002;7.

17. Nerurkar PV, Dragull K, Tang CS. In vitro toxicity of kava alkaloid, pipermethystine, in HepG2 cells compared to kavalactones. Toxicol Sci. 2004;79:106-11.

18. WHO. Assessment of the Risk of Hepatotoxicity with Kava Products. Geneva: World Health Organization, 2007:6-25.

19. CAC. Discussion Paper on the Development of a Standard for Kava Products. Madang, Papua New Guinea: Joint FAO/WHO Food Standards Programme, 2012.

20. Lasme P, Davrieux, F., Montet, D., Lebot, V. Quantification of kavalactones and determination of kava (Piper methysticum) chemotypes using near-infrared reflectance spectroscopy for quality control in Vanuatu. J. Agric. Food Chem. 2008;56:4976-81.

21. Lebot V, Levesque, J. Genetic control of kavalactone chemotypes in Piper methysticum cultivars. Phytochemistry. 1996;43:397-403.

22. Lebot V, Johnston, E., Zheng, Q.Y., McKern, D., and McKenna, D.J. . Morphological, phytochemical, and genetic variation in Hawaiian cultivars of ’awa (Kava, Piper methysticum, Piperaceae). Econ. Bot. 1999;53:407-18.

23. Siméoni PL, V. Identification of factors determining kavalactone content and chemotype in Kava (Piper methysticum Forst. f.). Biochem. Syst. Ecol. 2002;30:413-24.

24. Teschke R, Lebot V. Proposal for a kava quality standardization code. Food Chem Toxicol. 2011;49:2503-16.

25. Rowe A, Zhang LY, Ramzan I. Toxicokinetics of kava. Adv Pharmacol Sci. 2011;2011:326724.

26. Ho QT, Verboven P, Verlinden BE, Herremans E, Wevers M, Carmeliet J, Nicolai BM. A three-dimensional multiscale model for gas exchange in fruit. Plant Physiol. 2011;155:1158-68.

27. Walton WH. Feret's statistical diameter as a measure of particle size. Nature. 1948;162:329-30.
28. Schmitt M, Halisch M, Muller C, Peres Fernandes C. Classification and quantification of pore shapes in sandstone reservoir rocks with 3-D X-ray micro-computed tomography. Solid Earth. 2016;7:285-300.
29. Patterson B, Escobedo-Diaz, J., Dennis-Koller, D., Cerreta, E. . Dimensional Quantification of Embedded Voids or Objects in Three Dimensions Using X-Ray Tomography. Microsc. Microanal. 2012;18:390-8.
30. Jaiswal Y, Weber D, Yerke A, Xue Y, Lehman D, Williams T, Xiao T, et al. A substitute variety for agronomically and medicinally important Serenoa repens (saw palmetto). Sci. Rep. 2019;9:4709.
31. Van Noordwijk M, Brouwer G. Quantification of air-filled root porosity: A comparison of two methods. Plant and Soil. 1988;111:255-8.
32. Wongs-Aree C, Noichinda S. Glycolysis Fermentative By-Products and Secondary Metabolites Involved in Plant Adaptation under Hypoxia during Pre- and Postharvest. In: Kusal K. D.; Biradar MS, editor. Hypoxia and Anoxia. London, UK: Intechopen; 2018. p. 59-72.
33. Piekarska-Stachowiak A, Szymanowska-Pulka J, Potocka I, Lipowczan M. Topological traits of a cellular pattern versus growth rate anisotropy in radish roots. Protoplasma. 2019;256:1037-49.
34. Atkinson JA, Rasmussen A, Traini R, Voss U, Sturrock C, Mooney SJ, Wells DM, et al. Branching out in roots: uncovering form, function, and regulation. Plant Physiol. 2014;166:538-50.
35. Herremans E, Verboven, P., Verlinden, B.E., Cantre, D., Abera, M., Wevers, M., Nicolaï, B.M. Automatic analysis of the 3-D microstructure of fruit parenchyma tissue using X-ray micro-CT explains differences in aeration. BMC Plant Biol. 2015;15:1-14.
36. Barry JA, Groseclose MR, Robichaud G, Castellino S, Muddiman DC. Assessing drug and metabolite detection in liver tissue by UV-MALDI and IR-MALDESI mass spectrometry imaging coupled to FT-ICR MS. Int. J. Mass Spectrom. 2015;377:448-155.

37. Rosen EP, Bokhart MT, Nazari M, Muddiman DC. Influence of C-Trap Ion Accumulation Time on the Detectability of Analytes in IR-MALDESI MSI. Anal. Chem. 2015;87:10483-90.

38. Nazari M, Ekelof M, Khodjaniyazova S, Elsen NL, Williams JD, Muddiman DC. Direct screening of enzyme activity using infrared matrix-assisted laser desorption electrospray ionization. Rapid Commun. Mass Spectrom. 2017;31:1868-74.

39. Walden J, Von Wegerer J, Winter U, Berger M, Grunze H. Effects of kawain and dihydromethysticin on field potential changes in the hippocampus. Prog. Neuro-Psychopharmacol. Biol. Psychiatry. 1997;21:697-706.

40. Smith KK, Dharmaratne HR, Feltenstein MW, Broom SL, Roach JT, Nanayakkara NP, Khan IA, et al. Anxiolytic effects of kava extract and kavalactones in the chick social separation-stress paradigm. Psychopharmacology (Berl). 2001;155:86-90.

41. Lèvesque VLaJ. The origin and distribution of kava (Piper methysticum Forst. f., Piperaceae): a phytochemical approach. Allertonia. 1989;5:223-81.

42. Cairney S, Maruff P, Clough AR, Collie A, Currie J, Currie BJ. Saccade and cognitive impairment associated with kava intoxication. Hum. Psychopharmacol. 2003;18:525-33.

43. Pollock NJ. Sustainability of the kava trade. Contemp. Pacific. 2009;21:265-97.

44. Khodjaniyazova S, Nazari M, Garrard KP, Matos MPV, Jackson GP, Muddiman DC. Characterization of the Spectral Accuracy of an Orbitrap Mass Analyzer Using Isotope Ratio Mass Spectrometry. Anal. Chem. 2018;90:1897-906.

45. Tarbah F, Mahler H, Kardel B, Weinmann W, Hafner D, Daldrup T. Kinetics of kavain and its metabolites after oral application. J. Chromatogr. B. 2003;5:115-30.

46. Wang Y, Eans SO, Stacy HM, Narayanapillai SC, Sharma A, Fujioka N, Haddad L, et al. A stable isotope dilution tandem mass spectrometry method of major kavalactones and its applications. PLoS One. 2018;13:e0197940.

47. Smith RM, Thakrar H, Arowolo TA, Shafi AA. High-performance liquid chromatography of kava lactones from piper methysticum. J. Chromatogr. A. 1984;283:303-8.

48. Bokhart MT, Nazari M, Garrard KP, Muddiman DC. MSiReader v1.0: Evolving Open-Source Mass Spectrometry Imaging Software for Targeted and Untargeted Analyses. J Am Soc Mass Spectrom. 2018;29:8-16.

Figure legends

Figure 1. Specific kava roots and stems tissues selected for the study, and the applied statistical models. Two mixed linear models were made based on the selection of the various parts of *P. methysticum*. **CRP, CNP,** and **LR** denote the crown root peel, crown with no peel, and lateral roots. **SP** and **SNP** denote stem peels and stems with no peels, respectively. The plant parts are divided into stem, crown roots and lateral roots, thus there was a model for these structural locations with levels: stem, lateral root, and crown root. Finally the stems and crown roots were divided into "peel" or "no peel" and we used these, with the lateral roots, as the levels for the model with a term for tissue (Stems and crown roots "with peels" samples were excluded from the mixed linear model).

Figure 2. GC-MS Total Ion chromatograms of various parts of *P. methysticum* roots. **A**, peel of crown roots (**CRP**), **B**, crown root with no peel (**CNP**), **C**, crown root with peel (**CWP**), and **D**, lateral roots (**LR**). Some representative kavalactones and other compounds found in the extracts are denoted as δ-Cadinol (**c**), dihydromethysticin (**d**), α-epi-7-epi-5-Eudesmol (**e**), kavain (**k**) and desmethoxyyangonin (**y**).

Figure 3. PCA and Box plots for GC-MS and LC-MS analysis of kava roots and stems. PCA plots of **(A)** GC-MS, **(B)** LC-MS positive and **(C)** LC-MS negative mode indicate a clear distinction between roots (green) and stems (purple) for each dataset. PCA1 separated the crown roots, lateral roots and stems with adjusted p-values of the pairwise Student's t-test as [3.43E-05, 0.0003458] in **(A)**, [0.000475, 0.00257] in **(B)**, and [0.000585, 0.00274] in **(C)**, respectively. PCA1 also separates both crown roots (triangles) and lateral roots (circles) from stem (squares). The mixed linear model of the adjusted p-values for PCA1 and PCA2 of GC-MS, LC-MS positive, LC-MS negative mode are 2.037E-06, 0.00206, and 2.137E-05, respectively. The axes of each plot show the principal components and the percentage of the variance that they explain rounded to the nearest percent.

(D) kavain, **(E)** desmethoxyyangonin and **(F)** dihydromethysticin indicated in the boxplots, have adjusted p-values of 7.05E-06, 3.40E-06, and 7.05E-06 in the mixed linear model for different tissue types. Black bars below box plots indicate statistical significance from adjusted p-values from pairwise Student's t-test where '*' indicates $P \leq 0.05$, '**' indicates $P \leq 0.0$, and '***' indicates $P \leq 0.001$. Significant differences were observed between roots (green) and stems

(purple). **CRP, CNP,** and **LR** denote the crown root peel, crown with no peel, and lateral roots. **SP** and **SNP** denote stem peels and stems with no peels, respectively. These plots exclude the whole stem and crown sections (**SWP** and **CWP**).

Figure 4. X-ray µCT images of crown and lateral roots of *P. methysticum*.
(A-H) and **(I-P)** denote images of 3D surface reconstructions based on µCT data of crown and lateral roots, respectively.
(A) and **(B)** are the segmented air-filled spaces in parenchyma and the cork (peel). **(C)** represents the overlay of the cork and air-filled regions in parenchyma of the crown roots. **(D)** 3D rendering image of the whole section of crown root. **(E)** and **(F)** represent the transverse view images of crown roots in 3D rendering and grey scale, respectively. **(G)** and **(H)** represent the longitudinal view images of crown roots in grey scale and 3D rendering, respectively.
(I) and **(J)** are the segmented tissues of lateral roots, comprising of air-filled spaces in parenchyma and the cork (peel), respectively. **(K)** an overlay of the segmented regions of lateral roots including cork and air-filled regions in parenchyma. **(L)** 3D rendering image of the whole section of lateral roots. **(M)** and **(N)** represent the transverse view of lateral roots in 3D rendering and grey scale. **(O)** and **(P)** represent the longitudinal view images of lateral roots in grey scale and 3D rendering. The sections **a**, **b**, **c** and **d** represent cork, cortex, parenchyma and air-filled spaces, respectively.

Figure 5. IR-MALDESI ion abundance heatmaps for various constituents in different parts of *P. methysticum*. (**A**) Distribution of kavain (K), 7,8-Dihydro-5,6-dehydrokawain (5,6-Ddk), 7,8-dihydro-5-hydroxykavain (5-Dhk) *m/z* 231.1016, (**B**) Optical images of various parts (**C**) Distribution of desmethoxyyangonin (DMY), p-Hydroxykavain (p-Hk) *m/z* 229.0859 (**D**) Distribution of dihydromethysticin (DHM) *m/z* 277.107. Images in **a-c**, **d-f** and **g-i**, indicate lateral roots, crown roots and stem sample images, respectively.

Figure 6. MS-MS overlay spectra and fragmentation pattern of kavain obtained in Parallel Reaction Monitoring (PRM) MALDESI. (A) represent the MS-MS spectra of standard compound, **B** represents the fragmentation pattern of kavain, **C** and **D** represent the MS-MS spectra of kavain on crown root and stem tissues, respectively.

Tables

Table 1. Results of quantitative analysis of selected kava lactones by GC-MS in various plant parts and separated tissues of *P. methysticum*

Sample	Kavain (mg/g ± SD)	Dihydromethysticin (mg/g ± SD)	Desmethoxyyangonin (mg/g ± SD)
CWP	0.425 ± 0.235	1.689 ± 0.862	0.355 ± 0.138
LR	2.003 ± 0.615	2.842 ± 0.748	0.901 ± 0.182
SWP	0.042 ±0.012	0.290 ±0.110	0.080 ±0.015
CRP	0.987 ± 0.235	1.875 ± 0.467	0.660 ± 0.146
CNP	0.922 ± 0.187	2.055 ± 0.445	0.516 ± 0.088
SP	0.069 ± 0.021	0.500 ± 0.204	0.161 ± 0.044
SNP	0.128 ± 0.027	0.356 ± 0.067	0.165 ± 0.029

CWP, **LR** and **SWP** denote the whole crown roots, lateral roots and stems of *P. methysticum*, respectively. **CRP, CNP, SP** and **SNP denote** the crown root peel, crown root with no peel, stem peels and stems with no peels, respectively. The concentrations of kavalactones in selected plant parts were calculated on a dry weight basis.

Figure 1 Click here to download Figure Figure 1.tif

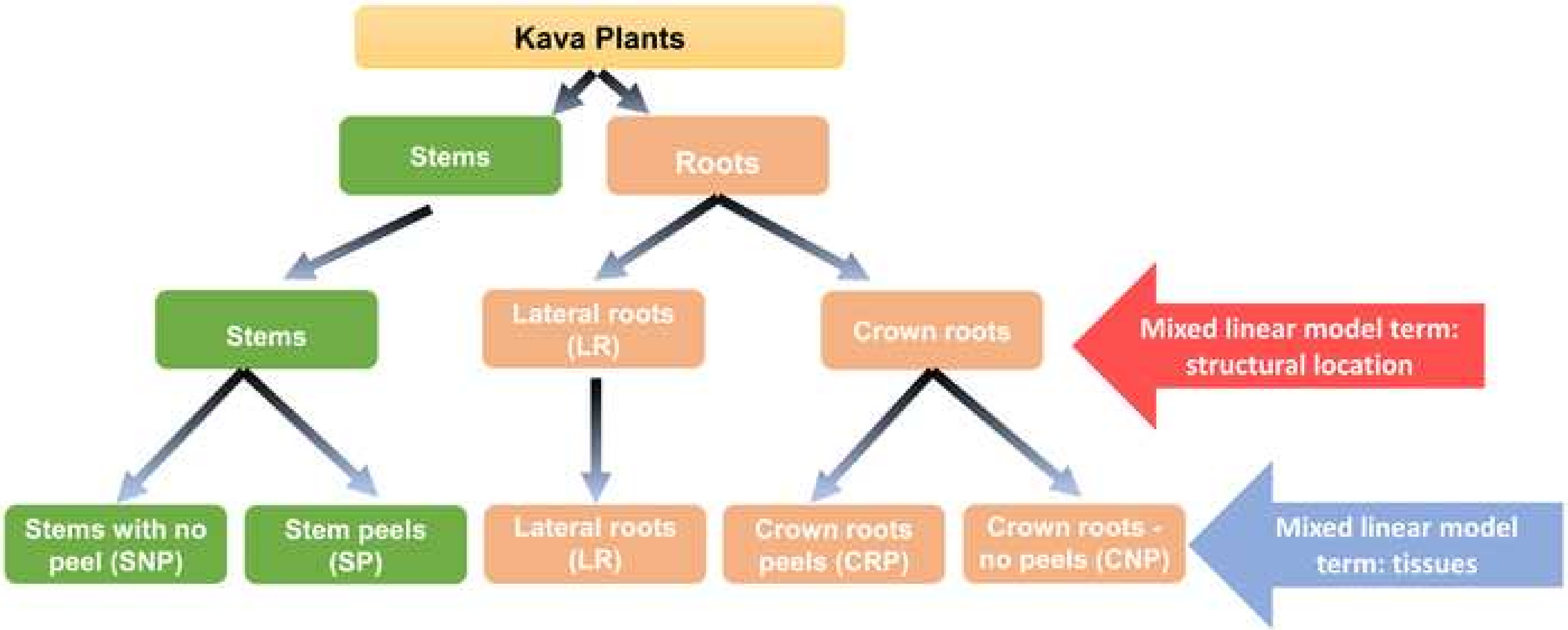

Figure 2

Click here to download Figure Figure 2.tif

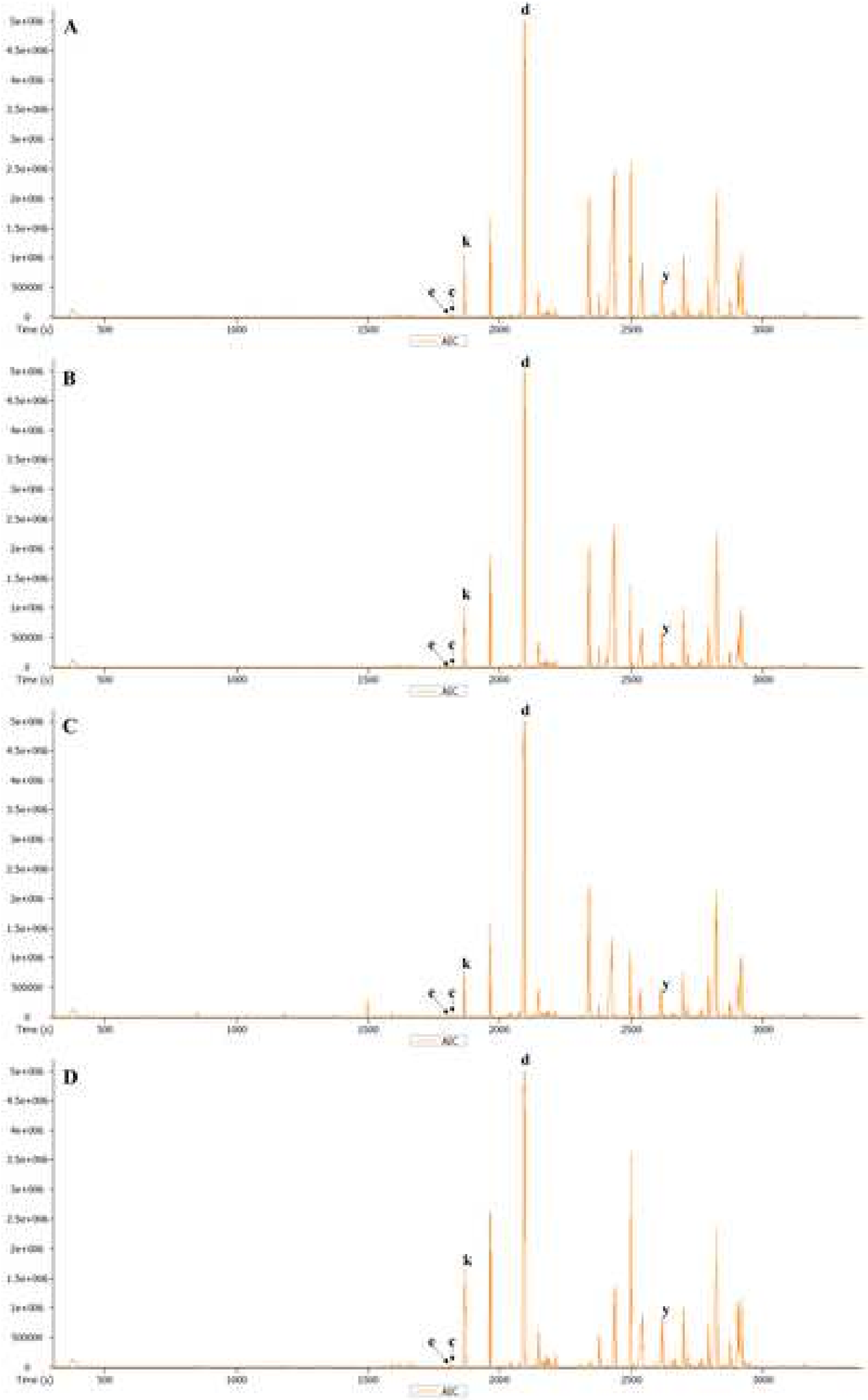

Figure 3 Click here to download Figure Figure 3.tif

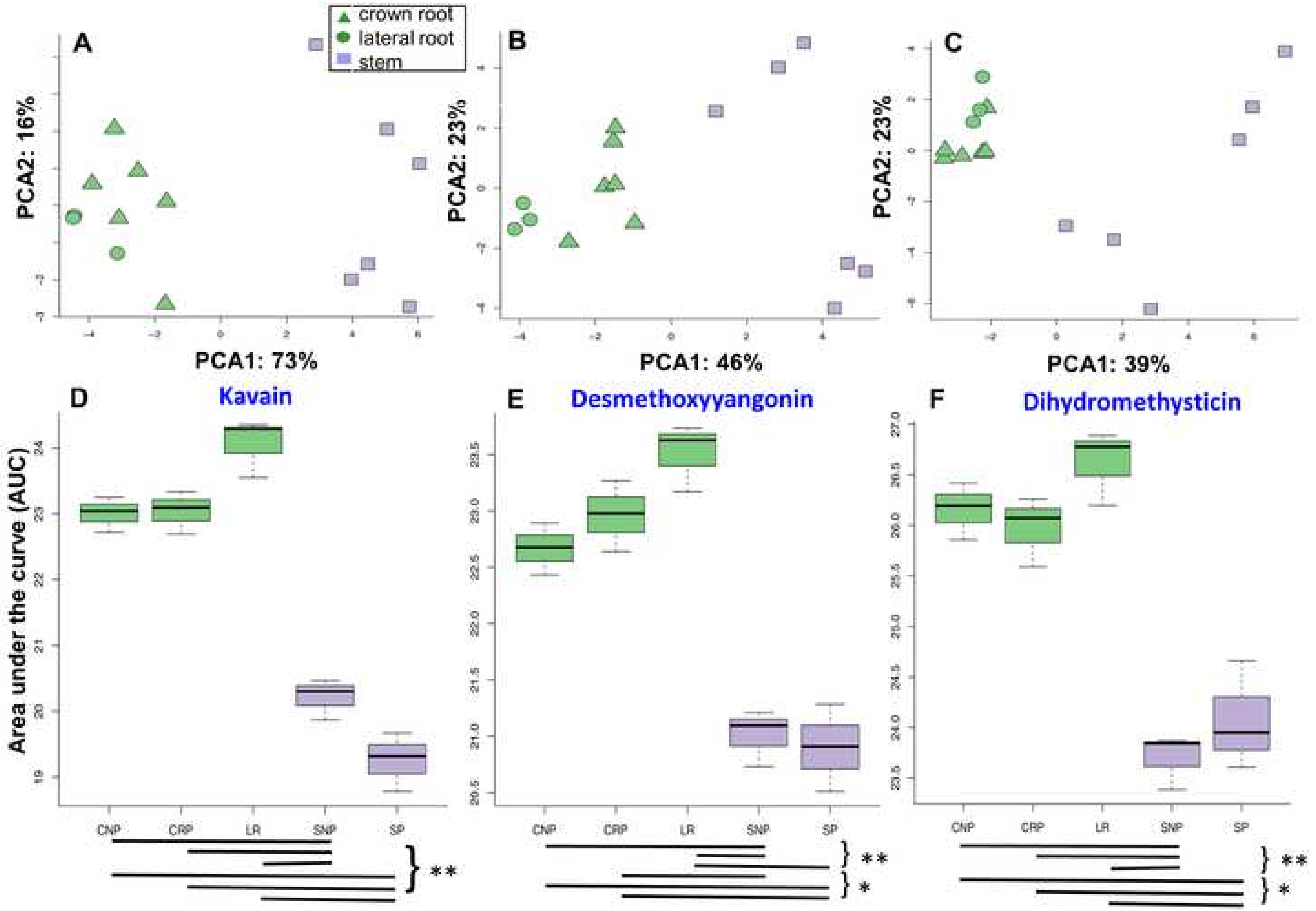

Figure 4

Click here to download Figure Figure 4.tif

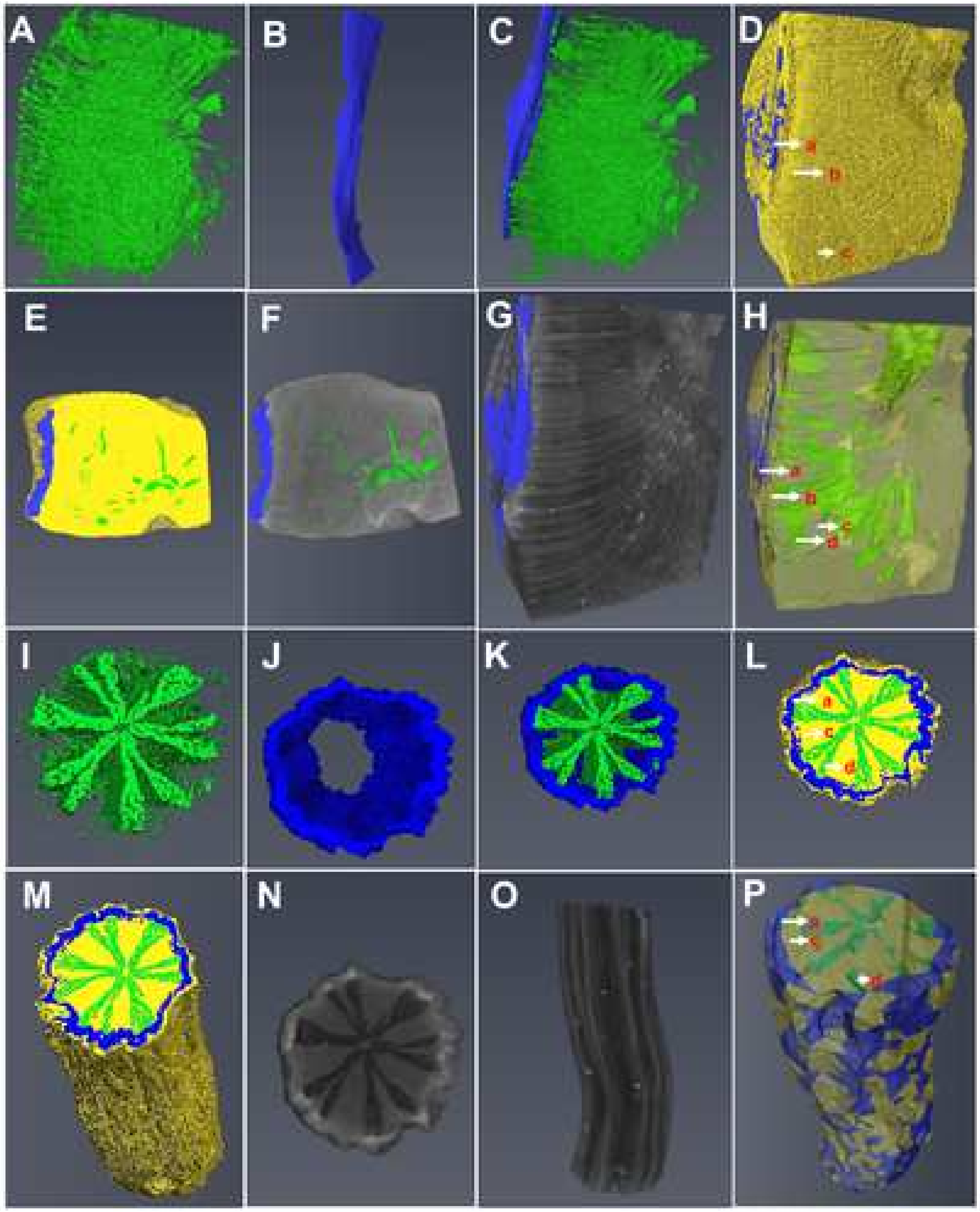

Figure 5

Click here to download Figure Figure 5.tif

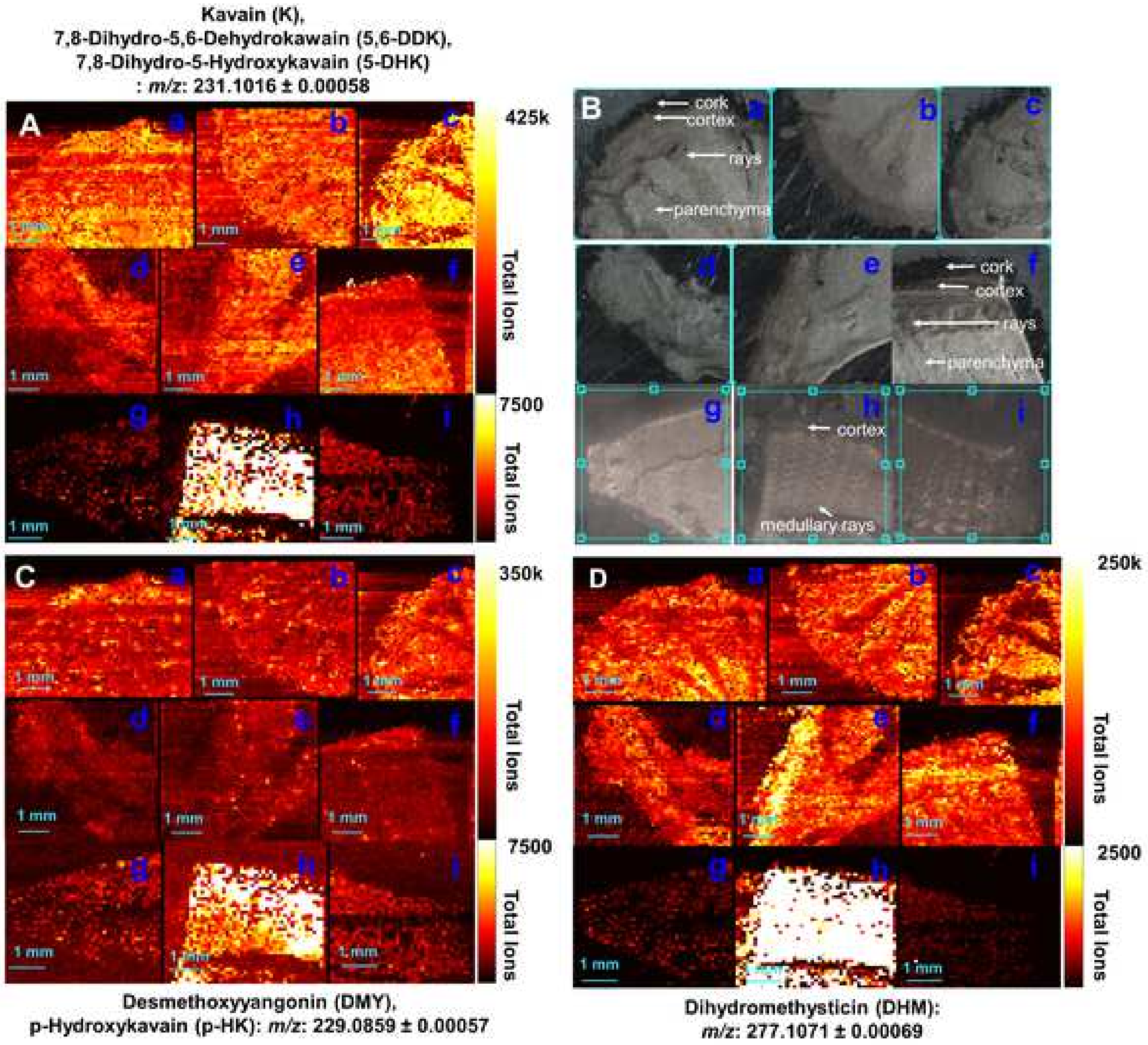

Figure 6 Click here to download Figure Figure 6.tif

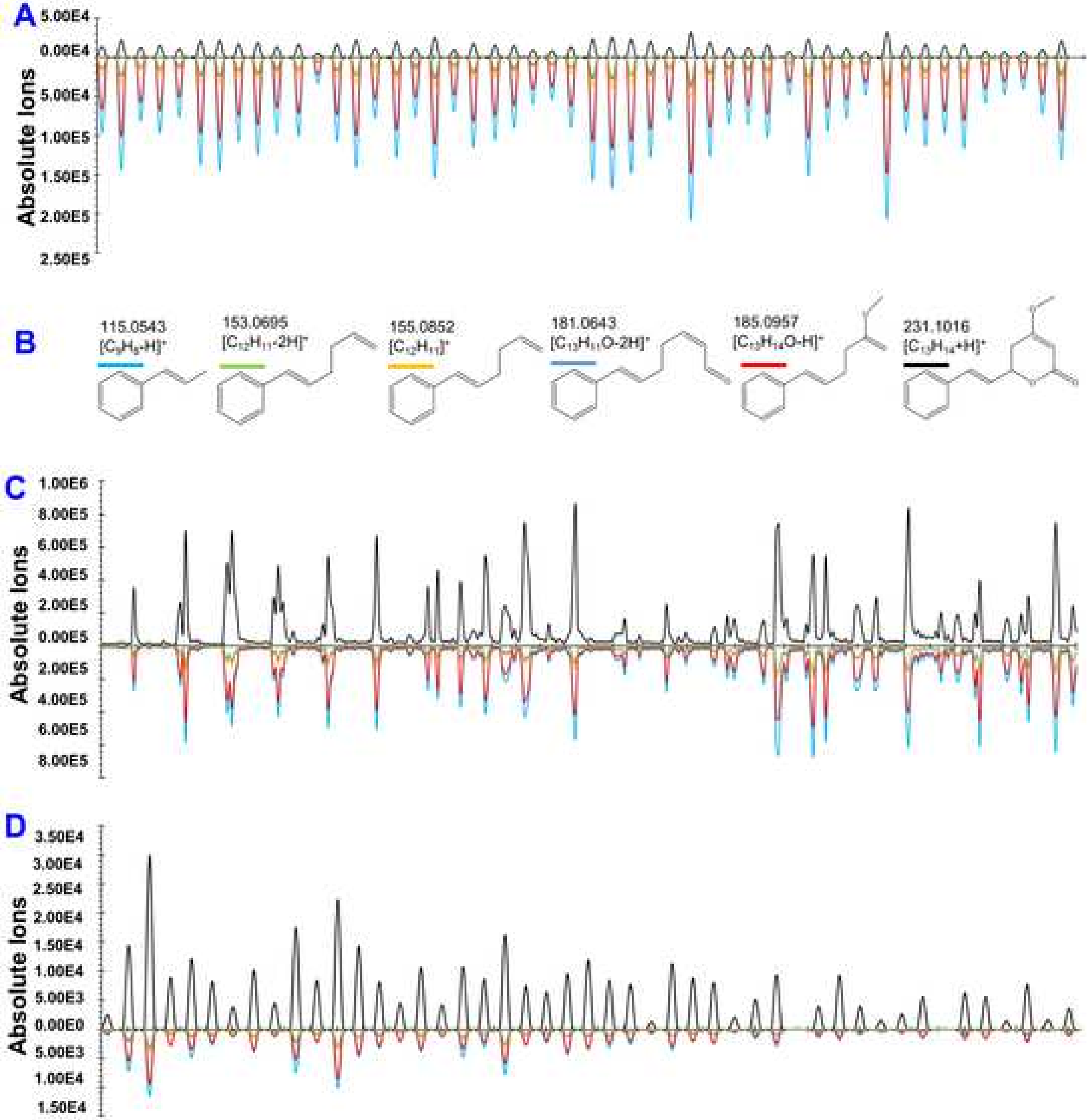

Click here to access/download
Supplementary Material
Additional Files.docx

Cover and response letter

Click here to download Personal Cover Cover letter- Giga Science - Dr. Williams.pdf

Date: 06.01.2020

The Editor
GigaScience, Oxford Academic Press

Sub: Revised article submission (MS. Ref.# GIGA-D-20-00029)

Title: 3D imaging and metabolomic profiling reveal higher neuroactive kavalactone contents in lateral roots and crown root peels of *Piper methysticum* (Kava)

Dear Editor,

Please find enclosed our revised research manuscript for consideration of publication in *GigaScience.* We sincerely thank the editor for the interest in our manuscript, and for this opportunity to resubmit it in revised form.

We appreciate the constructive critiques and the interest of the reviewers, and the editor for careful reading and consideration of our article. The insightful comments of reviewers have helped us improve our manuscript.

The reviewers' suggestions were adopted, and the manuscript has been suitably revised. Specifically, (i) additional data analysis were performed (ii) introduction and discussion sections were revised and, (iii) modifications to figures were carried out. A detailed point-to point response letter to the reviewers' comments, follows this cover letter. The changes mentioned above in the manuscript are marked in red font. The manuscript is suitably improved, and we hope it will be found satisfactory for further evaluation.

The findings of this study remain unchanged: The study investigates tissue specific secondary metabolite synthesis of *Piper methysticum* (Kava) by a combination of metabolomics, bioinformatics and imaging techniques. It reveals important correlation between tissue-specific secondary metabolite synthesis of kava, and the traditional practice of using only "peeled" kava roots for safe preparation of beverages. The results indicate that, whole lateral roots and peels of crown roots have the highest concentration of bioactive kavalactones that are responsible for its psychoactive properties, compared to the crown roots and stems. The findings of this study are of significance to scientists, herbal drug manufacturers, consumers, and health regulatory authorities, worldwide.

Publication in *GigaScience* will provide an effective impact on the wide range of readership, for supportive action towards quality control and safe use of this globally used therapeutically important medicinal plant.

Thank you again for consideration of our revised article.

Best regards,

Leonard L. Williams, PhD, MBA
Center for Excellence in Post Harvest Technologies
NC A&T State University, 500 Laureate Way,
Kannapolis, NC 28081, USA.
Phone: (704) 250-5700 ext. 5703, Fax: (704) 250-5709
Email: llw@ncat.edu

Response letter for Manuscript ref. no: GIGA-D-20-00029

The authors thank the reviewers for their valuable time and contributions in providing constructive and thoughtful critiques, and the editor for careful reading and consideration of our manuscript. We sincerely appreciate the inputs provided, that have improved our manuscript significantly.

In the revised manuscript, all the comments made by the reviewers have been suitably addressed. Specifically, (i) additional data analysis was performed for identification of more metabolites (ii) descriptions in results and discussion sections were improved, and (iii) modifications to the main and figures text, as recommended by reviewers were carried out.

A detailed point-to-point response for each of reviewers' comments and indications of following it, are detailed below. We hope that the reviewers will find the revised manuscript satisfactory for further evaluation.

Queries/critiques are numbered and in blue Times Roman font.

Responses follow in black Times Roman.

Revised text from the manuscript is indicated in red Times Roman.

Detailed responses to Reviewers' comments:

Reviewer reports:

Reviewer #1: This very interesting Research Article reports on imaging and metabolomic profiling of Kava. Tissue-specific kavalone distribution was observed in lateral roots and crown root peels of Piper methysticum (Kava). Matrix-assisted laser desorption electrospray ionization (IR-MALDESI) imaging was used to identify tissue-specific distribution of target kavalactones on cryosectioned material. Of note, IR-MALDESI imaging was performed at 100 micron resolution, which was sufficient for identifying tissue compartments in crown root and lateral root samples. Using this approach, the authors were able to demonstrate that lateral roots have a higher content of kavalactones than crown roots. In addition, microCT imaging was used to explore morphological characteristics associated with kavalactone synthesis. Void shape factor, Feret's diameters, 3D volumes, and anisotropy were calculated on the 3D image data and suggest that lateral roots are more suited to gasexchange and metabolism than crown roots. The manuscript is well written, and the scripts that were used in the analysis are all made publicly available on GitHub where they have been ascribed a GPLv3 license.

Response: We thank the reviewer for appreciating the interesting aspects and the significance of this study. We agree with the suggestions made by the reviewer and appreciate their time in pointing out the errors. These suggestions have improved our manuscript significantly.

Minor comments

1. In the manuscript the authors refer to the Python X-Ray Imaging Tool (pyXIT), a software tool for processing measured data in x-ray imaging. pyXIT is licensed as free software under the BSD license. Would it be possible for GigaScience to take a copy of this software?

Response: Our co-author is in process of discussing with the Institute, about the possibility of obtaining license. Due to the current pandemic situation, we will need to wait to obtain a final decision about this request.

2. The authors have submitted the 3D image data (3D TIFF format) for some of the crown root and lateral root samples outlined in the study. However, one of the image files - PCT_20170718_Kava_lateral_root_40_Piper_methysticum.tif - is not a 3D TIFF image. Rather it is a 2D 16-bit TIFF image. I invite the authors to submit the 3D image data for this lateral root specimen.

Response: Thank you for this suggestion. A 3D TIFF image will be uploaded in the GIGADB database, as recommended.

3. I cannot find Supplementary File S11 that details all parameters used in the image analysis. I invite the authors to submit this missing file.

Response: The authors thank the reviewer for pointing this mistake. We had incorrectly listed the file as S11. The file is now correctly mentioned referred to **Repository data Figure R7.** The repository is described in the manuscript.

- **Section:** Repository Index and Figure numbers R1-R9**.**
  Please refer: Line numbers 41-67, page 8-13.
  https://github.com/palomnyk/kava_3D_imaging_and_metabolomics

- **Section: Manuscript**
  Please refer: Line nos. 445-447, page 21
  Text: "Details of ……https://github.com/palomnyk/kava_3D_imaging_and_metabolomics".

4. The text on Figures 4D, 4H, 4L, and 4P obscures what the arrows are attempting to highlight. I invite the authors to amend this figure so that the text does not obscure the images.

Response: Thank you for this important observation. We have modified the labelling of Figure 4, as per your recommendation.

5. Additional data files are hosted on a second GitHub archive entitled 'kava_3D_imaging_and_metabolomics'. However, there is no license attributed to the latter GitHub archive. I invite the authors to ascribe an OSI-approved license to this archive to encourage reuse.

Response: We thank the reviewer for this suggestion. We have now added an OSI-approved license to the GitHub archive.

6. I wish to highlight to the authors that there is the additional option of utilising the GigaScience 3D viewer to enable interactive visualisation of 3D models generate by microCT. If the authors wish to use this facility, they should submit surfacerendered 3D images (STL format) for all of the crown root and lateral root samples used in the study.

Response: We appreciate this option highlighted by the reviewer. It introduced us to various software for converting 3D images to STL format. As suggested, we have converted the 3D micro-CT images of crown and lateral roots to STL format for interactive visualization. These files will be uploaded with the manuscript files.

The authors appreciate the contributions of the reviewer in providing these vital suggestions and helping us improve the quality of our manuscript.

Reviewer #2:
Jaiswal et al. have performed imaging and mass spectrometry analyses of kava (Piper methysticum) to characterize the distribution of kavalactones in various plant tissues. The work is rather descriptive and it is not very clear what problem are the authors actually trying to address. However, the produced datasets may be useful for researchers working on kava. My main concern is that the manuscript in its current form falls short of its goal to map the kavalactone distribution of kava.

Major comments:
1. The authors focus all their quantitative analyses on three kavalactones: kavain, desmethoxyyangonin and dihydromethysticin. However, there are at least 20 different kavalactone molecules in kava and 6 among them are considered "major kavalactones" (see Supplementary Figure 1 in doi:10.1038/s41477-019-0474-0). Some kavalactones have the same chemical formula and therefore mass (e.g., kavain and 7,8-dihydro-5,6-dehydrokavain), therefore the imaging results (Figure 5) that focus on 231.1016 m/z correspond to a superposition of these molecules. This aspect should be carefully considered by the authors. Furthermore, recently new kavalactone dimers have been reported (see doi:10.1080/10286020.2017.1367768 and doi:10.1007/s10600-019-02759-8), which might also be worth mentioning. The authors should definitely expand the scope of the analysis to all kavalactones, or clearly explain the reasoning why they only limit the analysis on the selected 3 molecules (which would, however, dramatically reduce the value of the paper).

Responses: The authors thank the reviewer for these important suggestions that have helped us improve our manuscript.

- As suggested, the indicated references have been referenced and cited in the manuscript.
  Section: Manuscript - Introduction
  Please refer: Line numbers 52-55, page 3 - Reference nos. 13-15
  Text: "The bioactive neuroactive compounds ………. reported to be isolated from kava roots [10-15]".

- As per the reviewer's suggestion, the recently identified dimers of kavalactones have been mentioned in the manuscript.
  Section: Manuscript - Introduction
  Please refer: Line numbers 54-55, page 3 - Reference nos. 13-15
  Text: "Novel dimeric kavalactones, ………….. to be isolated from kava roots [10-15]"

- Based on the recommendations of the reviewers, all the 20 kavalactones mentioned in the suggested article were analyzed. The results of analysis and occurrence of these metabolites in crown roots and lateral roots are indicated **Supplementary Figures S3** and **S4**.
  Please refer:
  Additional files – Supplementary data (Line numbers 102-111, page 56 – 58):
  Supplementary Figures S3.1, S3.2 and S4.

2. There are many cultivars of kava in the Pacific islands that exhibit different chemotypes (kavalactone content and/or distribution). See Lebot and Levesque, Phytochemistry 1996 (already referenced in the manuscript) and other publications by those authors (Lebot and Levesque, Allertonia 1989 or doi:10.1016/j.jfca.2016.01.009), which demonstrate the dramatic differences in kavalactone distribution among the different cultivars. That means the results obtained by Jaiswal et al. have a
limited scope based on which cultivar was analyzed. The authors say "We carefully control for the variety of the plant used and the tissues selected" (l.88), however I could not find any information in the manuscript regarding the kava cultivar that was used, the number of plants that were harvested and their cultivation conditions. The only description provided is "noble" variety (l. 97), which is still very vague.

Response: We thank the reviewer for pointing out this important aspect. As per the reviewer's suggestions the requested information about the kava samples collected, is provided.

Section: Manuscript - Methods
Please refer: Line numbers 275-279, page 13-14
Text: "Roots of noble kava variety ………… attached to the root."

Minor comments:

3. Thomson (Th) was a proposed unit for m/z values that was never accepted by IUPAC and has since been abandoned. Please just use "m/z" instead of "Th". See https://en.wikipedia.org/wiki/Thomson_(unit)

Response: We thank the reviewer for this important suggestion. All the occurrences of "Th" in the manuscript (including figures and text) have been corrected.

4. The value of Figure 2 showing total ion chromatograms with no annotation is close to zero.

Response: Thank you for this important suggestion. We have made the suggested corrections in Figure 2 and provided annotations of representative compounds.

5. Does it make sense to produce 3 different PCA analyses (Figure 3) based on the analytical technique? Why not merge the features found by the different techniques into a single dataset and perform the PCA analysis on the merged dataset?

Response: We thank the reviewer for this suggestion. However, merging the data from these different techniques would lead to problems with normalization and an overly sparse dataset. Hence, the data from different techniques cannot be merged.

6. The color scheme of Figure 4 is unclear.

Response: Thank you for this suggestion. It has helped us improve the clarity in the figure. We have provided annotations to each tissue type for enhanced clarity. Each tissue of the roots has

an autogenerated color from 3D segmentation. Each of the segments are described in figure legends.
Please refer: Line numbers 667, page 30
Section: **Figure 4:** "The sections **a**, **b**, **c** and **d** represent ……………. respectively".

7. Figure 6 is confusing. Where is the m/z axis of the MS/MS spectra? Why are there two different intensity axes (above and below 0)?

Response: We appreciate the reviewer's query about **Figure 6**. However, we wish to point out that, there is no *m/z* axis. The y-axis is abundance of the precursor (top) and MS/MS fragments (bottom) of the kavain *m/z* (231.1016). We feel confident to identify kavain vs other kava lactones with the same *m/z* due to the matching MS/MS fragmentation pattern when compared against a kavain standard. The two different intensity axes are to separate the fragments from the precursor and demonstrate that we analyze them at the same time as the precursor, leading to a positive identification of kavain.

8. The authors should deposit their raw mass spectrometry data into a public repository and the collected MS/MS spectra into a spectral database (e.g., GNPS or MoNA).

Response: Thank you for this important suggestion. As per the journal's policy we have submitted the raw mass spectrometry data and MS/MS spectra to their suggested "METABOLIGHTS" repository.

Once again, we thank the reviewer for their time in reviewing our manuscript and providing important suggestions that have improved our manuscript.

Reviewer #3:

Major comments:

1. Introduction: 4 paragraphs were spent on medical benefits of kava, authors should be more concise, there are too many unnecessary information. This would be fine if in manuscript Authors perform some pharmacological tests, but manuscript is entirely analytical MS oriented, so I suggest that Authors focus more in introduction on analytical approaches in studyng kava active principles and/or their spatial distribution (as described in references 27-32).

Response: We thank the reviewer for their insightful suggestion that has helped us improve the presentation of our manuscript. As per the reviewer's suggestions we have suitably modified the "Introduction" section of our manuscript. The introduction also contains newly added content as requested by other reviewers.

Section: Introduction
Please refer: Line numbers: 46-72, page: 3-4
Text: "Kava is a high in demand ………… kavalactone contents and profiles of other secondary metabolites."

2. Figure 5: Are the signals out of the tissue consequence of signal leakage, measurement artefact, normalization issues or something else? In metabolite imaging it is very important to don't have any signal out of tissue as consequence of molecules diffusion.

Response: Thank you for this query. The concentration of constituents in the tissues was very high. Thus, the out of tissue signals result from carryover from previous ionization shots. The signal carryover, however, is low enough that it does not obscure the spatial distributions of the endogenous kava lactones. Images can be provided with normalization to background peaks that more clearly define the boundaries of the roots.

3. Discussion Line 247-278: Authors should discuss results presented in the manuscript not again Kava pharmacological properties. Authors should relate these pharmacological properties with their results so that they relate pharmacological features, compounds and morphological features where detected compounds where found enriched or depleted. In the current form it looks unrelated and confusing to readers.

Response: We thank the reviewer for this important suggestion. It has helped us rewrite the discussion section in a concise way. The stated sections of discussion have been modified as recommended.

Section: Discussion
Please refer: Line numbers: 240-260, page: 12-13.
Text: "Kava has a wide presence in the global herbal……………. kava roots, their traditional use and the resultant pharmacological effects."

Minor comments:

4. Figure 2- Authors should annotate major peaks (or at least these one discussed in lines 115-117) directly in the Figure, otherwise it is hard to follow the Figure 2 and I need to go to supplementary file to search for specific peak/annotation.

Response: As per the reviewer's recommendation, Figure 2 has been annotated for the major peaks.

Please refer: Line numbers: 631-635, page: 29 (Figure 2).

5. Line 127-Authors should add what was overlap between pos and neg ionization mode in LC-MS and what was overlap between GC-MS and LC-MS

Response: As per the reviewer's recommendation, the suggested information was added to the manuscript.
Section: Manuscript (Results)
Please refer: Line numbers: 115-120 (previously line 128), page: 6
Modified text: Between positive and negative mode ………………… 2 non-kavalactones (bornyl cinnamate and pipermethystine).

6. Line 151- Figure 3A-3C doesn't show linear models for each metabolite.

Response: We thank the reviewer for their observation. The annotation has been altered such that it does not indicate the linear models for each metabolite analyzed.

7. Figure 3D-3F: Authors should put name of 3 metabolites on the graph to be easier to interpret.

Response: As per the reviewer's suggestion, the names of 3 metabolites have been added to Figure 3D-3F.

8. Figure 5: I see only ion images for 3 kava lactones, and Authors reported 5 in the manuscript. It would be nice to see all 5 lactones in the Figure in the manuscript, not as Supplementary figure. Also, instead of abbreviations, I think it is more comprehensive to put full name of lactones, names are not too long, and Authors have enough space in Fig for doing that. Fig5B annotations of morphological regions are not easy to read. White letters would be probably better choice.

Response: The suggested changes have been made to Figure 5 of the manuscript.

9. Line 288-291: Elaborate more significance of the study.
Response: We thank the reviewer for this suggestion. The significance of this study has been elaborated.
Section: Manuscript (Discussion)
Please refer: Line numbers: 265-272, page: 13
Modified text: The morphological …….recreational alternative to neuroactive drugs.

10. Line 398: instead of "resolution" use "step size", laser beam used is 150um, so it is disputable if resolution is 100um.

Response: We thank the reviewer for this observation. As suggested, the manuscript text has been modified.
Section: Manuscript (Methods)
Please refer: Line numbers: 388-389, page: 19
Modified text: **"**Imaging was performed at 100 um step size……roots and stems samples".

11. Line 400: I am not sure if this Journal accept use of Th as non-IUPAC or SI unit.

Response: Thank you for the suggestion. We have replaced Th to *m/z*.
Section: Manuscript (Methods)
Please refer: Line numbers: 390-391, page: 19
Modified text: "A positive ion mode was used for analysis with 100-400 *m/z* low … range".

Once again, we sincerely appreciate the time and effort invested by the reviewer in reviewing our manuscript, and providing vital suggestions.

Reviewer #4:

The authors in this interesting work have studied kava plant which is considered to be an important neuroactive medicinal plant. They use a combination of GC-MS, LC-MS and IR-MALDESI to characterize and compare the qualitative as well as quantitative metabolic profile and spatial properties of kavalactones present in the roots and stems of the kava plant. They also study and compare the morphometric parameters and geometric descriptors using X-ray computed microtomography. Based on their study the authors present several scientific findings and provide conclusions on why only peeled roots of the plant are used in traditional social practice. This works provides an intersting use case of application of cross-platform techniques to answer a biological question and has the potential to be publishable but will need major revisions to make it complete.

Response: We thank the reviewer for highlighting the importance of our study and for providing important suggestions that have helped us improve our manuscript further.

1. Some of my main concerns regarding this work are listed below:
- The authors already mention one of the short-coming of the study which is the very small sample size (n=2 individual plants). This is also my biggest concern. In order to make meaningful statistically significant conclusions a larger population size is needed (n>10). Larger the sampling population, distinct biological trends will be revealed, that may affect the current results presented in the paper.

Response: We thank the reviewer for indicating this point. We would like to mention that n=2 was used only for "qualitative" micro-CT imaging and not the "entire study" discussed in this manuscript. Line 193 (now line no 183, page 9) in previously submitted manuscript clearly indicates this. Micro-CT study was only used for qualitative imaging to gain insights into morphology of Kava roots. Imaging studies with use of techniques such as micro-CT or MRI are expensive, time consuming and scarcely available. Qualitative studies are seldom performed on sample numbers such as 10, although "quantitative" studies may use samples more than 2. Please refer to the following references as examples: doi: 10.1186/s13007-018-0367-7, doi: 10.1186/s13007-017-0256-5, https://doi.org/10.1038/s41598-018-35324-4

2. In the abstract as well as in the manuscript text, the authors term this work as an integrated multi-omics approach. However, they use all the mentioned techniques/platforms for metabolite profiling and metabolite imaging. It would be more appropriate to use the term cross-platform metabolomics in this case.

Response: As suggested by the reviewer, the term "integrated multi-omics approach" has been replaced by "cross-platform metabolomics" in the 'Abstract' section.
No other occurrences of this term were found elsewhere in the manuscript.
Section: Manuscript- Abstract
Please refer: Line 31-32, page 2
Modified text: "Here we characterized the metabolomic ….. cross-platform metabolomics and 3D imaging approach."

3. P4 line 84 - The authors mention that selection of appropriate plant parts and using the correct variety of kava plant are two important factors that determine the concentration of kavalactones and its pharmacological effects. The results of this study shed light on the first part wherein they present their findings on the concetration of kavalactones in different parts of the suitable variety of the plant (peeled and unpeeled stem, lateral root and crown roots). However, the study provides no insights or comparison on the metabolic profile of suitable versus non-suitable variety of kava plant. Adding this comparison would make the story complete and would add to the scientific evidence of using only a specific variety of the plant for medicinal purposes.

Response: We thank the reviewer for this important comment. The “noble” (suitable) varieties are the varieties which are recommended to be sold legally. The non-suitable (non-noble adulterant) varieties are considered illegal varieties to be sold, and hence are difficult to obtain for any purposes, including scientific studies.

As per the reviewer’s suggestion, based on the literature we have added insights on comparison of suitable and non-suitable varieties.

Section: Introduction
Please refer: Line 57-72, page 3-4.
Modified text: The Kava act of 2002 declares “noble” varieties of kava as the only legally cultivated ………. resultant kavalactone content, and pharmacological effects of kava.

4. It is quite difficult to read the manuscript while navigating between the main figures/tables, supplementary tables and the multiple repository figures and files. A reader can easily get lost doing this. It is not entirely clear why the authors selected to divide the additional supporting data (figures, tables, sheets) as supplementary and some in the repository. Important figures in the repository document can directly be put in a single supplementary file for ease while reading or everything entirely can be placed in the github repository. Quite some re-organization is important here.

Response: We thank the reviewer for this suggestion. We would like to mention that, we have organized the files in repository and supplementary data separately, due to the file size and nature of the data. For example, imaging datasets have large file size that cannot be accommodated as additional files and are better suited for upload on a publicly accessible database repository such as GigaDB, as recommended by the journal.
We comply with the file size and page restrictions for figures and tables in the main manuscript. Moreover, data provided in the repository also contains codes that cannot be made publicly available in a suitable format in a single file. Hence, the files are divided as supplementary and repository files based on the journal’s guidelines.
The data provided in the repository are extensive associated data and difficult to be accommodated as supplementary files.

5. None of the peaks shown in the GC/MS and LC/MS total ion chromatograms have been labeled/annotated making it very difficult to visualize them based on if they are kavalactones, dihydrochalcones or non-kava lactone compounds (example: Figure 2)

Response: We thank the reviewer for this important suggestion. All GC-MS and LC-MS chromatograms have been annotated, as suggested.
Please refer: Figure 2 and Repository Figures R1, R3-R6.

6. Repository files R10-1 to R10-6 are .number files. A NUMBERS file is a spreadsheet created by Apple Numbers, a spreadsheet program bundled with macOS. These files do not open with excel or notepad. The authors should provide a more generic format that is not OS specific.

Response: We thank the reviewer for this recommendation. We have uploaded the .CSV files which will be accessible for non-macOS users.

7. It looks like the GC-MS data was normalized using an internal standard (as mentioned on P16 line 322), whereas the LC-MS data has not been normalized in any way. Is there a specific reason for not normalizing the LC-MS data?

Response: We thank the reviewer for pointing out this. The LC-MS data was also normalized by internal standard method. We have provided the details in the revised text.

Section: Manuscript – Methods
Please refer: Line no. 297-298, page 14
Modified text: "An internal standard method was …………… prior to analysis".

Minor issues:

1. P5 line 94 - The title should either be - "Mass spectrometry based profiling reveals unique tissue-specific metabolite profiles" OR "Mass spectrometry reveals unique tissue-specific metabolite profiles"

Response: As suggested by the reviewer, the title has been modified.
Section: Manuscript – Results
Please see: Line no. 82, page 4.
Modified text: "Mass spectrometry based profiling ……………metabolite profiles".

2. Figure 5 (a,c,d) and Supplementary Figure S2 - For the IR-MALDESI reconstructed ion maps displayed in these figures it would be better have a uniform scale for the color gradients representing total ions for a better visual comparison.

Response: Thank you for the comment. We would like to mention that, a uniform scale for color gradients is not appropriate due to the wide range (several orders of magnitude) of abundances across metabolites in different parts of the kava root and stem system. If the scale is set to the high side, images with low abundance will not appear to be present, shown as black voxels. On

the other hand, if the scale is set to the low side, images would provide little visual spatial information due to highly abundant voxels, shown as white. These ion maps have been specifically chosen to illustrate the best possible spatial information, while also giving appropriate ion abundances.
Hence, a uniform scale for the color gradients representing total ions is not advisable.

3. P16 line 335 - It is important to provide the details on how the baseline smoothing and peak picking was performed. Which software/ algorithm or script was used for these preprocessing steps?

Response: Thank you for the suggestion. We have added the requested information in the below mentioned section of the manuscript.

Section: Manuscript - methods
Please refer: Line nos. 322-326, page 16.
Modified text: "Baseline smoothing, peak picking, …………… to quantify the data".

4. P17 line 353 - It would be nice to mention the version of the nlme package used.

Response: The version of nlme package has been added.
Section: Manuscript - methods
Please refer: Line no: 344, page 17

5. Figure 5 description (P32 line 723) - By grey scale images do the authors mean a photograph of the sections that have been imaged? In that case, these should be termed as "Optical images". Also, in this description text, for easier reading, m/z values and the acronyms should be mentioned together with the name. Example: (A) Distribution of kavain (K), m/z 231.101, (C)...Same suggestion applies for Supplementary figure S2

Response: We thank the reviewer for this important suggestion. We have suitably modified Figure 5 and Supplementary Figure S2.

Please refer: Manuscript - Figure 5 (Line no. 670-673, page 30) and Supplementary Figure S2- (Line no. 89-90, page 54)

Once again, we thank the reviewer for their time and important inputs in the review of this manuscript. The suggestions have helped us enhance the quality of our paper.
