# Supplementary material for: 3D Imaging and metabolomic profiling reveal higher neuroactive kavalactone contents in lateral roots and crown root peels of Piper methysticum (kava)
Source: Gigascience. 2020 Sep 22;9(9):giaa096. doi: 10.1093/gigascience/giaa096 (PMC7507772; doi:10.1093/gigascience/giaa096)
Supplement: giaa096_GIGA-D-20-00163_Revision_1 [file giaa096_giga-d-20-00163_revision_1.pdf]

GigaScience

3D imaging and metabolomic profiling reveal higher neuroactive kavalactone contents in lateral roots and crown root peels of Piper methysticum (Kava)

--Manuscript Draft--

**Manuscript Number:**	GIGA-D-20-00163R1
**Full Title:**	3D imaging and metabolomic profiling reveal higher neuroactive kavalactone contents in lateral roots and crown root peels of Piper methysticum (Kava)
**Article Type:**	Research
**Funding Information:**	Bavarian Ministry of Economic Affairs, Regional Development and Energy — Dr. Daniel Haddad<br>National Institutes of Health (R01GM087964) — Dr. David C. Muddiman<br>T32 Biotechnology Traineeship — Mr. M. Caleb Bagley
**Abstract:**	Kava is an important neuro-active medicinal plant. While kava has a large global consumer footprint for its clinical and recreational utilization, factors related to its use lack standardization and the tissue-specific metabolite profile of its neuroactive constituents is not well understood. Here we characterized the metabolomic profile and spatio-temporal characteristics of tissues from the roots and stems using cross-platform metabolomics and 3D imaging approach. GC-MS and LC-MS revealed the highest content of kavalactones in crown root peels and lateral roots. IR-MALDESI imaging revealed a unique tissue-specific presence of each target kavalactone. µCT analysis demonstrated that lateral roots have morphological characteristics suitable for synthesis of the highest content of kavalactones. These results provide mechanistic insights into the social and clinical practice of the use of only peeled roots by linking specific tissue characteristics to concentrations of neuroactive compounds.
**Corresponding Author:**	Leonard L. Williams<br>Center for Excellence in Post-Harvest Technologies<br>Kannapolis, NC UNITED STATES
**Corresponding Author Secondary Information:**	
**Corresponding Author's Institution:**	Center for Excellence in Post-Harvest Technologies
**Corresponding Author's Secondary Institution:**	
**First Author:**	Yogini S. Jaiswal
**First Author Secondary Information:**	
**Order of Authors:**	Yogini S. Jaiswal<br>Aaron Yerke<br>M. Caleb Bagley<br>Måns Ekelöf<br>Daniel Weber<br>Daniel Haddad<br>Anthony Fodor<br>David C. Muddiman<br>Leonard L. Williams
**Order of Authors Secondary Information:**	
**Response to Reviewers:**	Response letter for Manuscript ref. no: GIGA-D-20-00163<br><br>The authors sincerely appreciate the time and effort invested by the reviewers in

Powered by Editorial Manager® and ProduXion Manager® from Aries Systems Corporation

providing their valuable inputs. These suggestions have improved our manuscript significantly.
In the revised manuscript, all the comments made by the reviewers have been suitably addressed. Specifically, (i) Figures and figure legends have been corrected (ii) tables have been modified, and (iii) certain references have been deleted, as recommended by the reviewers.
A detailed point-to-point response for each of reviewers' comments and indications of following it, are detailed below. We hope that the reviewers will find the revised manuscript satisfactory for further evaluation.

Queries/critiques are numbered and in blue Times Roman font.
Responses follow in black Times Roman.
Revised text from the manuscript is indicated in red Times Roman.

Reviewer reports:
Reviewer #1: The authors addressed most of my concerns. However, I found some issues in the revised manuscript that should be fixed prior to publication.

1.The authors added additional references to introduction, but the referencing is quite confusing. Specifically, this sentence: "Novel dimeric kavalactones, namley diyangonins (A-C) have also reported to be isolated from kava roots [10-15]." References 10-13 have nothing to do with dimeric kavalactones.
Response: The authors thank the reviewer for pointing out this error. As per the recommendations, the non-relevant references have been deleted from the specified sentence.

Section: Manuscript - Introduction
Please refer: Line numbers 54-55, page 3 - Reference nos. 10, 11
Text: "Novel dimeric kavalactones, ………….. to be isolated from kava roots [10, 11]"

2.The authors claim they deposited their raw data into the Metabolights repository, but no such information (or deposition ID) is mentioned in the "Availability of Data and Materials" section.
Response: As per the reviewer's suggestion, the Metabolights repository/deposition ID is mentioned in the "Availability of supporting data and materials" section.
Section: Manuscript: Availability of supporting data and materials
Please refer: Line numbers 464-466, page 22
Text: "Metabolomics data have been deposited ………..with the identifier MTBLS1485".

3.Fig.5: The mass of 7,8-dihydro-5-hydroxykavain (5-DHK) ion is not 231.1016 m/z. Furthermore, p-hydroxykavain is a known metabolite of kavain in liver (doi:10.1016/S1570-0232(03)00046-1), but has it ever been reported in the plant?

Response: We thank the reviewer for pointing out these important errors. The necessary modifications have been carried out in Fig.5, and these two compounds have been removed from the figure.
Please refer: Figure 5 legend, Page 31, Line nos: 685-689
Text: Legend for Figure 5 – "IR-MALDESI ion abundance …….. (A) Distribution of kavain (K), 7,8-Dihydro-5,6-dehydrokawain (5,6-DDK): m/z 231.1016".

4.I do not understand Figure 6 at all. The legend says it contains MS/MS spectra, but it clearly doesn't contain any spectra (mass spectrum = m/z vs intensity). There is no label or scale for X axis! In their response the authors explained that "The yaxis is abundance of the precursor (top) and MS/MS fragments (bottom) of the kavain m/z (231.1016)." but they didn't add this description into the legend. I am missing clear description how the MS/MS experiments were performed (precursor selection, collision energy?).
Response: We thank the reviewer for pointing out this important error.
Our apologies for the confusion. During revisions we meant to change 'spectra' into 'chromatograms'. The X-axis in (A) is an arbitrary length of time that the standards were analyzed. In (C) and (D) the time scale is just from the beginning of the experiment to the end across tissue.
In Parallel Reaction Monitoring (PRM) mode in Orbitrap-based Thermo MS, every nth scan is dedicated to a full MS scan across the entire m/z range of interest, or one or

Powered by Editorial Manager® and ProduXion Manager® from Aries Systems Corporation

	another of select m/z of interest. In this particular case the 1st scan was a full MS scan from 120-480 m/z, the next was an MS/MS scan of 231.1016 with an isolation window of 1.5 m/z fragmented with NCE (normalized collision energy) = 30, the 3rd was an MS/MS scan of 277.1071 with NCE = 20, and the 4th was an MS/MS scan of 229.0859 with NCE = 35, then the cycle repeated. Each NCE value was optimized in unpublished experiments. We have attached the updated figure to make this more clear.<br>As per the reviewer's suggestions, the requested description is now added in the manuscript's "Materials and Methods" section and the figure legend is now modified. Please refer: Page 20, Line nos: 416-422 and Figure 6.<br><br>Text: In Parallel Reaction Monitoring (PRM) mode in …………………….was optimized in unpublished experiments (Figure. 6).<br><br>5.(a) Supplementary figures S2 to S5 need more careful preparation. The legend of Fig. S2 points<br>to images a-c, d-f, and g-i,which are not indicated in the figure.<br>(b) It is unclear why Fig S2 shows 3 kavalactones, Fig. S3.1 shows 15 kavalactones, Fig.S3.2 shows 10 kavalactones, and Fig. S4 shows 20 kavalactones.<br>(c) Dihydrokawain is labeled as 233.1172 m/z in Fig. S3.1 but 215.1066 m/z in Fig. S3.2, why? (d)The inclusion of p-hydroxykavain is questionable (see above).<br>(e) Fig. S5 suffers again from the superposition of some signals (231.1016 m/z is not only<br>kavain).<br><br>Responses: We thank the reviewer for queries. Following are the specific responses:<br>(a) We apologize for the oversights in Supplementary Figure S2 and the confusion they have caused. The legend and figure of Supplementary Figure S2 have been revised, as per the reviewer's sugegstions.<br>Please refer: Additional files: Page no-53-56, Supplementary Figure S2.<br>(b) The focus of the study has been much more oriented towards the 3 kavalactones shown in Figure S2, hence its own figure here. Figure S3.1 and S3.2 go together but were split up for clearer presentation as a Figure with them 5 sub-figure wide would make the images too small. Their purposes were to show that we analyzed more kavalactones than just the ones of focus shown in the main bodies and figures of the paper in the crown roots, and then S4 has the same purpose, but for lateral roots. Supplementary Figures S3.1 and S3.2 show constituents from crown roots and Supplementary Figure S4 shows constituents from lateral roots.<br>(c) Dihydrokawain was identified both times but the 233.1172 m/z was its proton adduct peak, and the 215.1066 is the proton adduct with water loss. The water loss proton adduct is a commonly identifiable peak in many of our studies.<br>(d) As suggested by the reviewers, all occurrences of p-hydroxykavain in figures and text have been deleted.<br>(e) Yes, not all the images we provide in the supplementary images are as high quality as others. Since we had performed MS/MS on kava roots and stems and compared it to the standard, the identification of 231.1016 m/z as kavain is a solid one. For other kava lactones putatively identified without MS/MS, we have to rely on a combination of biological and spectral verification. Supplementary Figures S2-S4 have been suitably revised.<br><br>6. The methods section needs a clear explanation how the metabolites in Sup. Tables S1 and S2 were identified, and confidence of the identification should be noted in the tables, see Blaženović, I., Kind, T., Ji, J. & Fiehn, O. Software Tools and Approaches for Compound Identification of LC-MS/MS Data in Metabolomics. Metabolites 8, 31 (2018).<br>Response: A clear explanation of how metabolites were identified in Table S1 and S2 is provided in the methods section. The confidence of identification are now mentioned in Tables S1 and S2.<br>(a) Please refer: Manuscript: Methodology, Page 16, Line nos. 327-332<br>Text: "Baseline smoothing, peak picking, automated ……. were used for metabolite annotation."<br><br>(b) Please refer: Manuscript: Methodology, Page 15, Line nos. 302-306<br>Text: "The resulting data …………..specific molecular ions and masses".

Powered by Editorial Manager® and ProduXion Manager® from Aries Systems Corporation

	(c) Please refer: Supplementary Tables S1 and S2 (Additional files: page 35-41).<br><br>We thank the reviewer once again for their efforts in providing us these important suggestions.<br><br>Reviewer #2: The authors have made substantial changes in the manuscript and have addressed all the issues that were raised for the previous version. The manuscript now reads well and there are no further comments or questions for the latest version.<br><br>Responses: The authors thank the reviewer for considering out manuscript favourably, and for the time and effort in providing us valuable suggestions to improve it.<br><br>Reviewer #3: I am satisfied how authors addressed all my questions with enough supporting data.<br>I have one minor comment that can significantly improve citation of your manuscript:<br><br>1. Please provide more detailed information on sectioning of kava roots. What was duration of soaking? Have you used some polymeric material to embed roots? There is a big challenge to section roots of woody plants without disturbing their morphology (without chemical fixation) and your optical images shows that you achieved that!<br><br>Response: We sincerely thank the reviewer for a positive approval of our manuscript and appreciating our work.<br>As per your recommendations, we have provided the requested information in the manuscript.<br><br>Please refer: Manuscript: Methods, Page: 18, Line nos. 364-371<br>Text: “The samples for cryo-sectioning were prepared ……..a polymeric gel upon freezing [43]”.<br><br>We thank the reviewer once again, for the efforts in providing us constructive suggestions and helping us improve our manuscript further.
**Additional Information:**	
**Question**	**Response**
Are you submitting this manuscript to a special series or article collection?	No
**Experimental design and statistics**<br><br>Full details of the experimental design and statistical methods used should be given in the Methods section, as detailed in our Minimum Standards Reporting Checklist. Information essential to interpreting the data presented should be made available in the figure legends.<br><br>Have you included all the information requested in your manuscript?	Yes
**Resources**	Yes

Powered by Editorial Manager® and ProduXion Manager® from Aries Systems Corporation

A description of all resources used, including antibodies, cell lines, animals and software tools, with enough information to allow them to be uniquely identified, should be included in the Methods section. Authors are strongly encouraged to cite Research Resource Identifiers (RRIDs) for antibodies, model organisms and tools, where possible.<br><br>Have you included the information requested as detailed in our Minimum Standards Reporting Checklist?	
**Availability of data and materials**<br><br>All datasets and code on which the conclusions of the paper rely must be either included in your submission or deposited in publicly available repositories (where available and ethically appropriate), referencing such data using a unique identifier in the references and in the "Availability of Data and Materials" section of your manuscript.<br><br>Have you have met the above requirement as detailed in our Minimum Standards Reporting Checklist?	Yes

Powered by Editorial Manager® and ProduXion Manager® from Aries Systems Corporation

Manuscript with DOI

Click here to access/download;Manuscript;Manuscript with DOI.docx

3D imaging and metabolomic profiling reveal higher neuroactive kavalactone contents in lateral roots and crown root peels of *Piper methysticum* (Kava)

Yogini S. Jaiswal*[1,7], Aaron M. Yerke[2,7], M. Caleb Bagley[3], Måns Ekelöf[3], Daniel Weber[4], Daniel Haddad[4,6], Anthony Fodor[2,6], David C. Muddiman[3,5,6], Leonard L. Williams*[1]

[1]*Center for Excellence in Post-Harvest Technologies, North Carolina Agricultural and Technical State University, The North Carolina Research Campus, 500 Laureate Way, Kannapolis, NC-28081, USA.* ***Emails:*** *llw@ncat.edu, yoginijaiswal@gmail.com*

[2]*Department of Bioinformatics and Genomics, University of North Carolina at Charlotte, Charlotte, North Carolina-28223, USA.* ***Emails:*** *afodor@uncc.edu, amyerke@uncc.edu*

[3]*Department of Chemistry, North Carolina State University, Raleigh, NC-27695, USA.* ***Emails:*** *mcbagley@ncsu.edu, moekeloe@ncsu.edu, dcmuddim@ncsu.edu*

[4] *Fraunhofer Development Centre X-Ray Technology EZRT, Division of Fraunhofer Institute for Integrated Circuits IIS, Department Magnetic Resonance and X-Ray Imaging MRB, Am Hubland D-97074 Wurzburg, Germany.* ***Emails:*** *Daniel.Haddad@physik.uni-wuerzburg.de, weber@mr-bavaria.de*

[5]*Molecular Education, Technology and Research Innovation Center (METRIC), North Carolina State University, Raleigh, NC-27695, USA.* ***Email:*** *dcmuddim@ncsu.edu*

[6]Co-Senior authors

[7]These authors contributed equally to this article

***Correspondence:** llw@ncat.edu, yoginijaiswal@gmail.com

Running title: Imaging and metabolomic profiling of Kava

Abstract

Kava is an important neuro-active medicinal plant. While kava has a large global consumer footprint for its clinical and recreational utilization, factors related to its use lack standardization and the tissue-specific metabolite profile of its neuroactive constituents is not well understood. Here we characterized the metabolomic profile and spatio-temporal characteristics of tissues from the roots and stems using cross-platform metabolomics and 3D imaging approach. GC-MS and LC-MS revealed the highest content of kavalactones in crown root peels and lateral roots. IR-MALDESI imaging revealed a unique tissue-specific presence of each target kavalactone. μCT analysis demonstrated that lateral roots have morphological characteristics suitable for synthesis of the highest content of kavalactones. These results provide mechanistic insights into the social and clinical practice of the use of only peeled roots by linking specific tissue characteristics to concentrations of neuroactive compounds.

Key words

Kava, kavalactones, metabolomics, 3D imaging, Mass Spectrometry imaging

Introduction

Piper methysticum Forster f. is a plant native to the Pacific region, and its roots and products are commonly known as "Kava" [1-3]. Kava is a high in demand medicinal plant famously known for its anxiolytic, sedative, psychoactive, and calming properties when used as a recreational beverage, herbal medicine, or as a dietary supplement [2, 4, 5]. Kava is an official medicine listed in many Pharmacopoeias and is used in folk medicine in the Pacific Islands [3, 5-9]. For over two decades, Kava cultivators and its market existence have continued to face the challenges of legislative and industrial disputes [4].

The bioactive neuroactive compounds from kava are the "kavalactones", and these are predominantly present in the roots. Pathways of enzymes that affect the biosynthesis of these compounds have been identified and reported by Qui et.al [10]. Novel dimeric kavalactones, namely diyangonins (A-C) have also reported to be isolated from kava roots [10, 11]. The Kavalactone profiles genetically vary among varieties. Based on the chemotypes, kava varieties are classified as *noble*, *medicinal* or *Two-Day* varieties. The Kava act of 2002 declares "noble" varieties of kava as the only legally cultivated varieties in Vanuatu, and very little information in literature is known for the non-noble varieties [12]. Irrespective of the variety, the kavalactone contents can vary in different organs of the plant. Thus, it is of vital importance to establish tissue-specific chemical profiles that can aid in selection of appropriate starting raw material.

Traditionally, only peeled roots have were used for preparation of beverages [2]. However, in kava bars, the plant parts (peeled or unpeeled root or stems), the varieties (noble or adulterant non-noble type), and the concentrations used, remain unregulated. The raw material sold in

markets is in the form of pre-cut pieces with no identification of plant parts used. Stems and stem peels are cheap adulterants used by vendors to substitute highly priced kava roots, and these are unsuitable for consumption. The stems contain high content of pipermethystine and is reported to be hepatotoxic, whereas the suitable plant parts (roots) do not have high contents of pipermethystine [13]. Thus, it is crucial to control the plant parts and varieties, which are of foremost importance among a multitude of factors that affect the resultant kavalactone content, and pharmacological effects of kava [14, 15]. There have been no studies published till date, that report tissue-specific kavalactone contents and profiles of other secondary metabolites.

In this study, we for the first time systematically explore the metabolites found in different parts of the kava plant. We expand on previous analytical work which used lower sensitivity instruments and did not discriminate between different tissues [2, 16-21]. We carefully control for the variety of the plant used and the tissues selected and use a combination of metabolomics and imaging technology to generate the most detailed picture to date of how metabolites differ in different specific tissues of the plant. This work represents an initial view of how social customs such as the use of the peeled roots of the plant can be linked to measurable metabolite concentrations of the neuroactive compounds.

Results

Mass spectrometry based profiling reveals unique tissue-specific metabolite profiles

In this study, we analysed peeled and unpeeled roots and stems for their kavalactone content to establish quantitative and qualitative tissue-specific metabolite profiles. Kava stems and roots of the “noble” variety with over three years of maturity were used. The tissues of the roots and

stems were selected for identification of the secondary metabolites by LC-MS, and quantitation of kavalactones by GC-MS analysis for three separate individual plants (**Figure 1**). Crown roots peels (**CRP)**, crown roots with no peels (**CNP**), crown roots with peels (**CWP**), lateral roots (**LR**), stem peels (**SP**), stems with no peels (**SNP**) and stems with peels (**SWP**) were the tissues selected for analysis. Quantitative GC-MS analysis of kavain, dihydromethysticin and desmethoxyyangonin revealed that the highest contents of these kavalactones were found in the lateral roots followed by crown roots and stems (lateral roots > crown roots > stems). This pattern was observed whether separated tissues groups were considered individually or not (**Table 1**).

Different constituents had different orders of concentration in separated tissues and whole plant parts. For example, in whole roots and stems content of dihydromethysticin was higher compared to kavain and desmethoxyyangonin. And in the separated tissues, content of dihydromethysticin was highest in peeled crown roots in contrast to kavain with highest content in peels of crown root.

In addition to quantitative analysis, we also performed untargeted metabolite profiling by GC-MS, which revealed the presence of 7 kavalactones, 3 dihydrochalcones and 19 non-kava lactone compounds **(Figure 2**, **Supplementary Table S1,** and **Repository Figures R1** and **R2**). These profiles revealed that δ-Cadinol and α-epi-7-epi-5-Eudesmol are distinctly present in all tissue parts of the lateral and crown roots. Pipermethystine and benzenepropanal were found in the whole stem and hydrocinnamic acid was found only in the stem peels. Overall, it was found that the crown roots and lateral roots have a higher number of constituents compared to the stems.

Except for the differences in tissue specific occurrence of some metabolites discussed above, all other metabolites were found in common among the crown roots, lateral roots and stems.

Untargeted LC-MS analysis in both positive and negative mode was also performed and 14 kavalactones, 3 dihydrochalcones and 19 non-kava lactone compounds were putatively identified **(Supplementary Table S2, Repository Figures R3-R6)**. Qualitative LC-MS shows minor differences between the presence of secondary metabolites in LC-MS positive and LC-MS negative modes. In the LC-MS positive mode, there were 14 metabolites found that were not found in the LC-MS negative mode. There were 12 compounds found in LC-MS negative mode that were not seen in the LC-MS positive mode (**Supplementary Table S3**). Between positive and negative mode ionisation of LC-MS analysis, 14 kavalactones, 3 dihydrochalcones, and 19 non-kavalactones were found in common (**Supplementary Table S2**). The common metabolites identified in GC-MS and LC-MS were 6 kavalactones (kavain, dihydromethysticin, dihydro-5,6-dehydrokawain, dihydrokavain, desmethoxyyangonin and yangonin), 3 dihydrochalcones (flavokawains A-C) and 2 non-kavalactones (bornyl cinnamate and pipermethystine).

Statistical modelling reveals that crown roots and lateral roots are similar in metabolite signatures but have a large difference from stems

PCA 1 separates both roots from the stem samples

We performed PCA ordination on 15 samples from 5 tissues and considered the results at the structural location (red arrow, **Figure 1**) and tissue level (blue arrow, **Figure 1**). At the structural location level, PCA analysis revealed complete separation of stems from both crown roots and lateral roots for GC-MS (**Figure 3A**) and LC-MS positive (**Figure 3B**) and negative

mode (**Figure 3C**). In addition, we observed separation between crown roots and lateral roots for PCA1 of the LC-MS negative (**Repository File R10-1**), and PCA7 of the LC-MS positive datasets (**Repository File R10-2**), although as we might expect, this separation was not as strong as the separation between the root types and stems and was not observed for all spectrometry methods.

At the tissue level, PCA1 for GC-MS differentiates CNP, CRP, and LR from SNP, whereas LC-MS negative differentiates the CNP, CRP, and LR from the SP tissues (**Repository File R10-3**) (Student's t-test). PCA1 from LC-MS negative also discriminates CNP from LR (**Repository File R10-4**) (Student's t-test). These results demonstrate that there are subtle differences in metabolite profiles between tissues that are detectable by our methods.

Mixed linear model shows that crown and lateral roots differ little from each other in metabolic profile

While the PCA data (**Figure 3A-3C**) gives insights into the overall structure of the dataset, it does not allow for determining the distribution of individual metabolites. Therefore, for the GC-MS data, which is the only quantitative spectrometry method we used, we built an initial series of linear models for each metabolite with a fixed term for structural location (with levels "crown root", "lateral root", and "stem") and plant number as a random effect. Using a False Discovery Rate (FDR) adjusted threshold of $p <= 0.05$, the "structural locations" term showed significant associations with 21 of the 28 metabolites (**Supplementary Table S4**). Pairwise testing via Student's t-test of each metabolite with a $p <0.05$ Benjamini-Hochberg corrected p-value revealed that the majority of the significant differences were between the "stem" and the two

root types (**Repository File R10-5**) with only 5 of the 43 pairwise significant tests between "crown root" and "lateral roots". The metabolites that were significantly different between the root types were flavokavain C, pinostrobin, hedycaryol, δ-cadinol, and bornyl cinnamate (**Repository File R10-5**, denoted by "#").

These "structural locations" data give a broad picture of the metabolites in different parts of the plant. In order to develop a more refined understanding, we built a second series of mixed linear models with a term for tissue type (with levels: "LR", "CRP", "CNP", "SP", and "SNP") and plant as a random effect. These 'tissue type" models showed significant associations for 24 of the 29 metabolites for the tissue type term (**Supplementary Table S4**). Of the 354 pairwise tests, 104 were significant (**Repository File R10-6**). However, only 6 of these significant tests were between lateral root and crown root tissues, and they were all CNP vs LR. In addition to squalene, the same 5 metabolites were found significant in the "structural location" model (**Repository File R10-6**, denoted by "#"). None of the significant metabolites different between CNP and LR are kavalactones, which indicates that the lateral roots and crown roots have very few differences in presence of kavalactones, but that they are both different from the stem tissues in terms of kavalactone profiles.

Pairwise analysis shows that kavain, desmethoxyyangonin, and dihydromethysticin are elevated in roots compared to stems

Of all the kavalactones for which we built statistical models, kavain, desmethoxyyangonin, and dihydromethysticin are especially of interest due to their well-known neurological activities amongst other kavalactones in the plant [10, 11, 22]. Consistent with the traditional use of roots

in folk medicine concentrations of all three of these metabolites were significantly lower in both stem tissue types than all of the root samples based on FDR adjusted Student's t-tests for all 29 tested metabolites (**Figure 3D-3F**) and quantitative GC-MS analysis (**Table 1**) [17]. All 3 metabolites appear to have a higher concentration in the lateral roots than the crown roots, however, our statistical analysis was unable to significantly differentiate these metabolites in the isolated tissues.

Lateral roots have morphological characteristics suitable for highest content of kavalactone synthesis

While kava roots serve as a major source of kavalactones, study of their morphological features remains an unexplored area . We therefore investigated the 3D topological structures of lateral and crown roots with X-ray computed microtomography using (μCT) **(Figure 4, Supplementary Figure S1 and Repository Figure R7)**. The 3D images provide visualization of internal tissue structure and insights into morphology and function relationship. Due to limited sample size (n=2 individual plants), application of rigorous statistical analysis was not possible for these images. However, to explore the differences in tissue properties that can be correlated to differences in metabolite synthesis, we calculated various geometrical descriptors on the available image datasets [23, 24]. Void shape factor and Feret diameter$_{max}$ showed a fold difference greater than 4.0 and 1.6, respectively, suggesting that lateral roots and crown roots are clearly distinguishable in the morphological characteristics that affect their gas exchange properties. We found that crown roots have a higher Feret diameter$_{max}$ and the air-filled spaces appear to be more unstructured, wide and merged compared to lateral roots where the air-filled

spaces are more structured and in the radial direction along the medullary rays **(Supplementary Table S5** and **Repository video files S1** and **S2)** [25, 26].

The volume of intercellular spaces, sphericity of voids and % porosity, were comparatively higher in lateral roots than the crown roots indicating a more intricate and highly connected air space network **(Supplementary Table S5)** [27-29]. Anisotropy, which affects morphogenesis of plant organs [30] had values that were higher for the crown roots compared to lateral roots indicating higher morphogenesis in crown roots [31]. The void shape factor values of crown roots were found to be higher than lateral roots (Table S5) and may have a correlation with the correspondingly high anisotropy values [32]. Based on the results of morphometric parameters and geometrical descriptors found in this study, we suggest that the lateral roots exhibit characteristics for gas-exchange and metabolism, that can be considered to be better than crown roots. These findings are in agreement with the results of quantitative analysis by GC-MS, where lateral roots were found to have the highest content of kavalactones. While future work with a larger sample size will be required to determine the statistical significance of these associations, these data do suggest that lateral roots have tissue structures for better gas exchange and metabolite synthesis compared to crown roots.

On-tissue mass imaging reveals kavain has a higher *in-situ* abundance in all tissues of crown roots

While spectrometry analyses used in this study are informative, they required disruption of tissue structures for metabolite extraction. In order to visualize the *in planta* distribution of

kavalactones in the stems, lateral and crown roots, prior to any processing or extraction, we used IR-MALDESI analysis.

Of the six kava lactones analysed, kavain (*m/z*: 231.1016), dihydrokavain (*m/z*: 233.1172) and yangonin (*m/z*: 259.0965) had a relatively higher abundance compared to dihydromethysticin (*m/z*: 277.1071), methysticin (*m/z*: 275.0914) and desmethoxyyangonin (*m/z*: 229.0859) (**Figure 5**). In the lateral roots, kavain is found to be abundant in the parenchyma, whereas in crown roots it is found in all three tissues (parenchyma, cork and cortex). Dihydromethysticin, methysticin, dihydrokavain and yangonin were found to be the most abundant in the parenchyma with a lower abundance in the cork and cortex region of both the types of roots (**Supplementary Figure S2-S4**). The stem tissues show lower abundance of all kavalactones tested, except desmethoxyyangonin when compared to the lateral and crown roots. Abundance of desmethoxyyangonin was found to be uniform through all roots and stems samples.

Identifications from IR-MALDESI are based on high resolution and accurate mass. However, as plant tissues have a complex secondary metabolite profile, we also validated these identifications by measuring spectral accuracy in addition to mass measurement. Isotope count heat maps generated by carbon counting based on spectral accuracy for ^{12}C, ^{13}C-1 revealed that, the on-tissue signals for all the ions identified were highly similar as the ones identified in abundance heat maps, confirming the robustness of our identifications across methods **(Supplementary Figure S5)** [33-35]**.**

Validation of the identification of target kavalactones in plant tissues was carried out by MS/MS fragmentation of standards, and comparison of the generated fragment ions and overlay of

spectra from standards and the plant tissues (**Figure 6A** and **Repository Figures R8** and **R9)**. The ratios of fragments of standard kavain, matched with the ratios of fragments obtained from root and stem tissues. This demonstrates that the identification of kavain in crown root and stem samples is valid and confirmed by MS/MS analysis.

Discussion

Kava has a wide presence in the global herbal market for its calming and recreational uses, with an unregulated product range consisting of beverages, herbal drugs and dietary supplements. There is a rising need to validate through scientific investigation the traditional practice of using 'only peeled' kava roots for preparation of beverages. With each kavalactone having a complex array of neurological effects (psychotic, anxiolytic and mood stabilizing), it is also important to characterize the tissue-specific presence and contents in kava roots [36-38]. This study represents an important initial step towards this goal.

In this study, the quantitative tissue-specific analysis by GC-MS revealed that, among the separated tissues, the crown root peels had the highest concentration of kavain and desmethoxyyangonin. Desmethoxyyangonin and kavain are reported to be absorbed faster than other kavalactones and cause a sudden euphoric "high" [2, 39]. The practice of using only peeled roots may avoid undesired effects of sudden euphoric "high" in consumers of kava beverages made with peels. The findings of this study provide scientific evidence, that agrees with the traditional practice of using only 'peeled' roots.

The concentration of unregulated beverages served in kava bars (*Nakamals*) are reported to be 150 times of the therapeutic dose, often leading to acute intoxication, cognitive impairment and

dissociative (hallucinogenic) [40, 41]. The quantitative analysis in this study reveal that, the lateral roots had the highest concentration of all three target kavalactones, kavain, dihydromethysticin and desmethoxyyangonin.

Our study discloses the important correlation between, tissue-specific secondary metabolite synthesis in kava roots, their traditional use and the resultant pharmacological effects. The state-of-the-art non-invasive imaging techniques, and analysis of the unprocessed plant tissues overcomes the drawbacks of the previously published studies by mapping *in-situ* metabolite profiles of kava. By investigation of metabolites biosynthesized in specific tissues of kava plant, the study provides future avenues for harvesting medicinally important kavalactones, in discovery of anti-epileptic and sedative hypnotic drugs from natural sources. The morphological and mass spectrometry-based identification of kava, provide data that can aid in distinguishing adulterants and undesired plant material in raw material used for preparing kava products. The findings of this study are important for kava product manufacturers, food regulatory authorities and consumers, for safe selection of kava plant parts for product formulation and consumption. The significance of this study lies in addressing the basic, but very critical issues that lie in the current unregulated and unstandardized use of kava, that has become a globally widespread tranquility recreational alternative to neuroactive drugs.

Methods

LC-MS analysis

Roots of noble kava variety named "Loa Leka" were collected in the last week of May 2017 from Taveuni, Fiji. The samples were more than three years of maturity and provided as gift samples

from Haridaya Enterprises Ltd., Fiji. About 6 kgs of the root samples were collected, with three samples each for selected parts of the plant. At the time of harvest the stump portion was left attached to the root. Samples of roots and stems of *P.methysticum* were analysed by an Agilent 7890A GC system, coupled to an Agilent 5975C electron ionization (EI) mass selective detector (MSD) and a UPLC-QTOF MS system (Acquity UPLC-SYNAPT MS, Waters Corp., Milford, MA). For liquid chromatography–mass spectrometry (LC-MS), samples were analysed after extraction of powdered plant material in ethanol (0.5 mg/ml) with ultrasonication (Elma, Elmasonic P30H) at room temperature for 30 mins. For untargeted profiling of the prepared extracts with LC-MS analysis, a UPLC-QTOF MS system was used. It was equipped with an ACQUITY BEH UPLC C_{18} analytical column (i.d. 1.7 μm, dimensions 2.1 × 100 mm, Waters, MA). The analyses were performed in both positive and negative electrospray ionization (ESI) to obtain comprehensive coverage in profiling. In positive ESI mode, the mobile phase comprised of 0.1% formic acid in water (solvent A) and 0.1% formic acid in acetonitrile (solvent B). In negative ESI mode, 1mM ammonium fluoride in water (solvent A) and acetonitrile (solvent B) constituted the mobile phase. The gradient used in positive mode ESI was: 0-1 min (1-15% B), 1-3 min (15-50% B), 3-8 min (50-85% B), 8-10 min (85-100% B), 10-11 min (100% B), 11-11.5 min (100-1% B), 11.5-13 min (1% B). For negative mode ESI the gradient used was: 0-1 min (1-20% B), 1-3 min (20-60% B), 3-6 min (60-85% B), 6-8 min (85-100% B), 8-11 min (100% B), 11-11.5 min (100-1% B), 11.5-13 min (1% B). Leucine enkephalin was used as a lock mass standard in both positive and negative modes ($[M+H]^+$ 556.2771 Da and $[M-H]^-$ 554.2615 Da). An internal standard method was used for normalisation. To each sample, 2 μg of para-chloro-phenylalaine was added as an internal standard, prior to analysis. The flow rate was set to

0.4ml/min with capillary voltages of 3.2 and 3.5 in positive and negative ESI modes, respectively. The desolvation temperature was set to 350 ºC and the mass range used was 50-1000 Da. The raw data files obtained from LC-MS analysis were processed using Progenesis QI software (Waters Corp., Milford, MA). The resulting data set was organized in a matrix including sample information, labels for all the detected peaks (retention time mass pairs) and an intensity determination for each detected peak. The accurate mass information was used for identification of compounds. METLIN metabolite search and PubChem databases were used for verifying the identity of all the metabolites, by comparison of their specific molecular ions and masses.

GC-MS analysis

For gas chromatography–mass spectrometry (GC-MS), powdered plant material were extracted in acetone (0.25g/ml) with the same conditions as LC-MS samples. Each of these samples were further diluted with 2ml of acetone. All the samples were centrifuged at 12000 rpm for 10 min (Centrifuge 5427R, Eppendorf). The extracted supernatants were stored at 4 ºC until analysis. To each sample, 20 µl of Docosanoic acid methyl ester was added as an internal standard (200 µg/ml). The samples were dried under nitrogen gas flow, derivatized with Trimethylsilyl (TMS) (Fisher Scientific, USA), and incubated at 70 ºC for 60 min prior to analysis.

The three standards kavain, dihydromethysticin and desmethoxyyangonin were purchased from Avachem Scientific, San Antonio, USA. All solvents used for analysis were of mass spectrometry grade. The quantitative and qualitative analyses were performed on three samples of each selected experimental group.

A DB-5MS capillary column (30 m length, 250 μm i.d., 0.25 μm film thickness) was used with helium as the carrier gas with a flow rate of 1 mL min^{-1}. Splitless injection mode was used with oven program set to 50 ºC initial temperature for 1 min, and then ramped up to 280 ºC at the rate of 50 ºC for 5 min. The transfer interface temperature was set to 280 ºC, injection volume 1μl, electron energy -70V and MS source temperature at 230 ºC. Scan mode was used for acquisition of characteristic ions and recording their retention times (mass range *m/z* 45-600). The raw data was processed by baseline smoothing and peak picking. Baseline smoothing, peak picking, automated and manual peak identification and peak integration were performed using LECO ChromaTOF software (version 4.51.6.0). As part of the method development process, a data processing method to integrate specific ion masses at specific retention times was developed to quantify the data. Peak identifications were performed manually and through the automated output. All identifications were manually interrogated and corrected, as necessary. NIST library search and PubChem databases were used for metabolite annotation. For quantitative analysis, calibration curves of standard compounds of kavain, desmethoxyyangonin and dihydromethysticin were constructed.

For quantitative analysis using GC-MS analysis, the raw data were processed using Agilent Chemstation, exported in .aia format and processed using LECO ChromaTOF software (4.51.6.0, Leco Corporation, MI). Calibration curves of standards were constructed for quantitative analysis of the kavain, dihydromethysticin and desmethoxyyangonin in all the selected samples. Normalisation with internal standard was carried out prior to use of data for statistical analysis.

Methods applied for development of statistical visualization models

Principal Component Analysis (PCA): The metabolite data were transformed using the R-stats function prcomp for multi-dimensional scaling (PCA) using Euclidean distance. In brief, this function produces a multi-dimensional matrix, which is then divided by axis where variance is highest. The function then returns a matrix of the 1D axis in order of highest variance to lowest. For most analyses presented, only the top three axes were used.

Mixed Effects Linear Model: From the nlme library in R (version 3.1-144), a mixed effects linear model was used to investigate the fixed effects of the metadata data categories and random effects of plant number on the metabolite data (metabolite ~ metadata + plant number) and on the results of the PCA (PCA ~ metadata + plant number). Significance was determined with the ANOVA function in R.

One-way ANOVA: Using R's Base ANOVA function, a one-way ANOVA was used to evaluate the significance of the metabolites data and the PCA data of the metabolites, as grouped by the metadata categories (tissue type, plant part, root vs. stem, whole section vs part of section, only peel vs not only peel, peel present vs peel not present, and plant number). P-values were adjusted using the Benjamini–Hochberg method. $P < 0.05$ was arbitrarily set as the significance threshold.

Student's t-test: Student's t-test was used to test pairwise comparisons using R's base t.test function. P-values were adjusted using the Benjamini–Hochberg method. $P < 0.05$ was arbitrarily set as the significance threshold.

Cryo-sectioning of samples

The samples for cryo-sectioning were prepared with a modified method published in one of our previous studies [42]. Kava roots and stem samples were wrapped in non-cellulose paper moistened with ultrapure water for 12 hours at room temperature. The samples were then kept overnight under vacuo at 25 inHg, prior to cryo-sectioning to facilitate softening of tissues by infiltration of moisture. The samples were cut into sections (about 1.5-2 cm in diameter) to enable mounting on the cryostat block. The embedding of the cut sections on cryostat blocks was accomplished by use of Surgipath Cryo-Gel matrix (Leica microsystems, Germany). Cryo-Gel is a water-soluble viscous liquid that forms a polymeric gel upon freezing [43]. Cryo-sectioning of tissues was carried out with a Leica CM1950 Cryostat (Buffalo Grove, IL, USA) at -20°C. Sections with thickness of 25 μm were prepared and carefully thaw mounted on pre-cleaned glass microscope slides.

IR-MALDESI analysis

The slides with sections were mounted on water-cooled Peltier stage with XY motion control, housed within the custom MALDESI enclosure. The enclosure was purged with N_2 gas until a relative humidity of <10% was reached, at which point the Peltier stage was cooled to -10°C. Allowing some time for temperature equilibration, the enclosure was opened, and the sample was exposed to ambient relative humidity. This resulted in formation of a thin ice layer forming over the sample. The enclosure was closed again, and the relative humidity kept constant to ~8-12% throughout analyses. A mid-IR tunable laser (IR Opolette 2371, OPOTEK, Carlsbad, CA, USA) tuned to 2.94 μm was used to fire at the tissue, resulting in desorption of neutrals from the tissue. This occurred by resonance excitation of the O—H stretching mode of water

endogenously present in the sample tissues and the created ice layer. The desorbed neutrals were encountered with an orthogonal electrospray plume that ionized them in an ESI-like manner. Ions from each desorption event were synchronously analysed in a Q Exactive Plus (Thermo Fisher Scientific, Bremen, Germany) with the automatic gain control (AGC) turned off to match the pulsed nature of IR-MALDESI. Instead of AGC, a fixed injection time (IT) was used to accumulate ions resulting from the laser pulses firing at 20 Hz. Over the *m/z* range of 100-400, the achieved resolving power was 140,000 (FWHM, *m/*z=200). The mass accuracy was parts per million (ppm), and lock mass calibrants (source 47 in polarity switching) were used for calibration.

Tissue sample ablation was performed with a 150 μm beam profile and images were captured at 100 μm step size, to ensure complete tissue ablation due to oversampling. Imaging was performed at 100 μm step size, and it was found adequate for visualisation of the structural features of the roots and stems samples. A positive ion mode was used for analysis with 100-400 *m/z* low mass-to-charge range. Initially ions were identified by the monoisotopic, $[M+H^+]^+$ *m/z* of each molecule. Followed by this step, the spectral accuracy (SA) was determined to ensure that the identified m/z value had the appropriate $^{13}C_1$ isotope ratio, for the respective naturally occurring compound. Isotope Count Heatmap compares the A+1 peak to the A peak as a certain percentage. This percentage is divided by the percentage of carbon that is naturally ^{13}C (ranging between 0.96-1.15%). In this study a ~1.12% was used, and each pixel was plotted by how many estimated carbons away it was from the original compound's *m/z* value. Images were then constructed with each voxel correlating to the appropriate desorption event and instrumental analysis. Carbon counting based on spectral accuracy for ^{12}C, $^{13}C_1$ was carried out for selected

kavalactones, to characterise the samples [33, 34]. The ‘Isotope Count Heatmap’ function in MSiReader was used to plot the estimated carbons for each of the target kava lactones [44].

Tandem-MS analysis in parallel reaction monitoring (PRM) mode with the same instrument settings, was used to determine presence of kavain (K), desmethoxyyangonin (DMY) and dihydromethysticin (DHM). Parallel Reaction Monitoring (PRM) mode was employed for fragmentation of the precursor ion and the other fragments generated. Each generated fragment was identified using predictive software (metfrag) and compared against published reports in literature [45, 46]. Fragments of the target compounds from tissues were compared with standard compounds and literature reports [47]. In Parallel Reaction Monitoring (PRM) mode in Orbitrap-based Thermo MS, every nth scan is dedicated to a full MS scan across the entire m/z range of interest, or one or another of select m/z of interest. In this particular case the 1st scan was a full MS scan from 120-480 m/z, the next was an MS/MS scan of 231.1016 with an isolation window of 1.5 m/z fragmented with NCE (normalized collision energy) = 30, the 3rd was an MS/MS scan of 277.1071 with NCE = 20, and the 4th was an MS/MS scan of 229.0859 with NCE = 35, then the cycle repeated. Each NCE value was optimized in unpublished experiments (**Figure. 6**). MSiReader (v1.01k), a freely available software developed in house, specifically for mass spectrometry imaging, was employed to ensure the identity of ions of interest visualized at given *m/z* [48].

X-ray microtomography analysis

The µCT analyses were performed using a mobile compact table-top system, developed at Fraunhofer Development Center X-Ray Technology EZRT (beam energy 50 kV). Two samples

each, from lateral and crown root which showed the best representation of morphological features and were devoid of any morphological damage were used for analysis. Two image data sets of each, the crown and lateral roots were used for data analysis. A reference capillary of known diameter was used to measure resolutions from the images. The samples were mounted vertically on the rotary stage and their fixation was confirmed, prior to exposure to SR light. Beam energy of 50 kV was used with isotropic nominal resolution of 38.1 and 35.3 µm/pixel for crown roots and 17.3 and 17.2 µm/pixel for lateral roots, respectively. The exposure time for each sample during scanning was 400 ms. The distances between the scanner and samples for each of the crown roots were 183.4 mm and 169.9 mm, and for the lateral roots the distances were 83.3 mm and 82.8 mm, respectively. A total of 3200 projections were recorded with 360° rotation steps.

Image processing of SR-µCT data

The postprocessing of the acquired µCT projections was performed using the "Python X-Ray Imaging Tool" (pyXIT) software (Maximilian Ullherr, Department of X-ray Microscopy, University of Wuerzburg, Germany, see https://www.physik.uni-wuerzburg.de/lrm/forschung/software/). From the µCT data of crown and lateral roots, 3D surface reconstructions were rendered using the Avizo Fire software 9.3.0 (FEI, Oregon, US) and subsequently analysed using Avizo Fire again. 3D rendering and segmentation of various parts of the roots were carried out by applying a project protocol developed for visualization and segmentation. The protocol steps included creation of ortho slices, labels for segmentation of various root parts, resampling, surface generation and viewing. Images were labelled and segmented into regions including the exterior, the whole root, intercellular air spaces and the

epidermis. Resampling of the labelled fields was carried out prior to generation of surface, to shrink the dimensions of the grid and facilitate ease in surface generation. Surface view function was used for 3D rendering and visualization of the μCT images of the root samples. The porosity of the samples analysed was calculated by applying the ASBMR module. Geometrical descriptors of tissue structures that correlate to their gas-exchange functions were calculated, to identify the correlation between structure and secondary metabolite profiles of the lateral and crown roots. Feret's diameters, 3D volumes, anisotropy etc. were calculated by applying arithmetic and label analysis module. Details of all parameters used in image analysis are provided in **Repository data Figure R7** in [49].

Availability of supporting data and materials

For statistical analysis, R studio version, 1.0.143 and R 3.5.1 (Feather Spray) were used for all computations and data manipulations. Metabolomics data have been deposited to the EMBL-EBI MetaboLights database [50] (DOI: 10.1093/nar/gkz1019, PMID:31691833) with the identifier MTBLS1485. Codes for all tests can be found at [51]. Additional repository data files are located in [49]. Snapshots of our code and other supporting data can be found in the *GigaScience* repository, GigaDB [52].

Additional files

Supplementary Table S1. Metabolites identified in roots of *P.methysticum* by GC-MS analysis.

Supplementary Table S2. Metabolites identified in common in various parts of *P.methysticum* by LC-MS analysis.

Supplementary Table S3. Secondary metabolites in various tissues of *P.methysticum* identified by LC/MS analysis

Supplementary Table S4. P-values of mixed linear model with GC/MS data

Supplementary Table S5. 3D morphological and geometric descriptors of roots of *P. methysticum.*

Supplementary Figure S1. Pictorial representations of plant parts of *P.methysticum* and their μCT imaging and sectional views.

Supplementary Figure S2. IR-MALDESI ion abundance heatmaps for various constituents in different parts of *P. methysticum*.

Supplementary Figure S3. Isotope Count Heatmap for kavalactones on various tissues of *P.methysticum*.

Abbreviations

3D: three dimensional; GC-MS: gas chromatography-mass spectrometry; LC-MS: Liquid chromatography-mass spectrometry; IR-MALDESI: infrared matrix-assisted laser desorption electrospray ionization; μCT: X-ray micro-computed tomography; GAD: generalized anxiety disorder; WHO: world health organization; FAO: food and agriculture organization; CRP: crown roots peels; CNP: crown roots with no peels; CWP: crown roots with peels; LR: lateral roots; SP: stem peels; SNP: stems with no peels; SWP: stems with peels; PCA: principal component analysis; FDR: false discovery rate; EI: electron ionization; MSD: mass selective detector; UPLC-QTOF MS: ultra-high performance liquid chromatography-quadrupole time-of-flight

mass spectrometry; ESI: electrospray ionization; TMS: trimethylsilyl; 1D: one dimensional; ANOVA: analysis of variance; AGC: automatic gain control; IT: injection time; K: kavain; DMY: desmethoxyyangonin; DHM: dihydromethysticin; PRM: parallel reaction monitoring; pyXIT: python X-Ray Imaging tool

Consent for publication

Not applicable

Funding

D.W. and D.H. acknowledge financial support by the Bavarian Ministry of Economic Affairs, Regional Development and Energy. This study received financial assistance from the National Institutes of Health grants R01GM087964 and T32 Biotechnology Traineeship T32GM008776 (M.C.B).

Author contributions

Y.S.J. and L.L.W designed and initiated the study. Y.S.J., D.W., D.H., M.C.B, M.E., performed the research. A.F., D.C.M., D.H. and L.L.W. provided assistance and expert opinions in design, analysis and execution of the experiments. Y.S.J., A.M.Y., M.C.B., and A.F., wrote the manuscript and analysed the data. A.M.Y. and A.F., wrote the Python and R scripts for statistical analysis of data and generating visualization plots.

Acknowledgments

The Center for Excellence in Post-Harvest Technologies appreciates the support of Mr. Abhishek Sapra of Haridaya Enterprises Ltd., Fiji, for the in-kind donation of kava samples and arranging the logistics for plant sample collection. No conflict of interest declared.

References

1. WHO. Piperis methystici rhizoma. WHO Monographs on Selected Medicinal Plants. Geneva: World Health Organization, 2002;2:231.
2. Lebot V. An overview of kava production in the Pacific Islands: what we do know and what we don't know. J. South Pacific Agric. 1997;4:55-62.
3. Purdel C. Assessment report on Piper methysticum G. Forst., rhizoma. Committee on Herbal Medicinal Products (HMPC), London, UK, 2017.
4. FSANZ. Kava A Human Health Risk Assessment. Wellington, New Zealand: Food Standards Australia New Zealand, 2004:1-26.
5. Singh YN. Kava: an overview. J. Ethnopharmacol. 1992;37:13-45.
6. SPC. Pacific Kava-A producer's guide: Secretariat of the Pacific Community (SPC), Hawaiian Kava Center, Fiji Islands 2001.
7. Goldberg AB, Mark. Herbal Medicine. Austin, TX: American Botanical Council; 2000.
8. BPC. Kavae Rhizoma. British Pharmaceutical Codex: Authority of the Council of the Pharmaceutical Society of Great Britain; 1907. p. 566.
9. Blumenthal M, Busse WR. The complete German Commission E monographs : therapeutic guide to herbal medicines. In: Blumenthal M, editor. Boston, MA: American Botanical Council 1998;xxii:685.
10. Yang JX. A New Kavalactone Dimer from Piper methysticum. Chemistry of Natural Compounds. 2019;55:606-9.
11. Yuan Y, Yang JX, Nie LH, Li BL, Qin XB, Wu JW, Qiu SX. Three new kavalactone dimers from Piper methysticum (kava). J Asian Nat Prod Res. 2018;20:837-43.
12. FAO. Kava Act 2002: An Act to regulate the cultivation, sale and export of kava and kava products. Vanuatu Legislation: Republic of Vanuatu, 2002;7.
13. Nerurkar PV, Dragull K, Tang CS. In vitro toxicity of kava alkaloid, pipermethystine, in HepG2 cells compared to kavalactones. Toxicol Sci. 2004;79:106-11.
14. WHO. Assessment of the Risk of Hepatotoxicity with Kava Products. Geneva: World Health Organization, 2007:6-25.

15. CAC. Discussion Paper on the Development of a Standard for Kava Products. Madang, Papua New Guinea: Joint FAO/WHO Food Standards Programme, 2012.

16. Lasme P, Davrieux, F., Montet, D., Lebot, V. Quantification of kavalactones and determination of kava (Piper methysticum) chemotypes using near-infrared reflectance spectroscopy for quality control in Vanuatu. J. Agric. Food Chem. 2008;56:4976-81.

17. Lebot V, Levesque, J. Genetic control of kavalactone chemotypes in Piper methysticum cultivars. Phytochemistry. 1996;43:397-403.

18. Lebot V, Johnston, E., Zheng, Q.Y., McKern, D., and McKenna, D.J. . Morphological, phytochemical, and genetic variation in Hawaiian cultivars of 'awa (Kava, Piper methysticum, Piperaceae). Econ. Bot. 1999;53:407-18.

19. Siméoni PL, V. Identification of factors determining kavalactone content and chemotype in Kava (Piper methysticum Forst. f.). Biochem. Syst. Ecol. 2002;30:413-24.

20. Teschke R, Lebot V. Proposal for a kava quality standardization code. Food Chem Toxicol. 2011;49:2503-16.

21. Rowe A, Zhang LY, Ramzan I. Toxicokinetics of kava. Adv Pharmacol Sci. 2011;2011:326724.

22. Pluskal T, Torrens-Spence MP, Fallon TR, De Abreu A, Shi CH, Weng JK. The biosynthetic origin of psychoactive kavalactones in kava. Nat Plants. 2019;5:867-78.

23. Ho QT, Verboven P, Verlinden BE, Herremans E, Wevers M, Carmeliet J, Nicolai BM. A three-dimensional multiscale model for gas exchange in fruit. Plant Physiol. 2011;155:1158-68.

24. Walton WH. Feret's statistical diameter as a measure of particle size. Nature. 1948;162:329-30.

25. Schmitt M, Halisch M, Muller C, Peres Fernandes C. Classification and quantification of pore shapes in sandstone reservoir rocks with 3-D X-ray micro-computed tomography. Solid Earth. 2016;7:285-300.

26. Patterson B, Escobedo-Diaz, J., Dennis-Koller, D., Cerreta, E. . Dimensional Quantification of Embedded Voids or Objects in Three Dimensions Using X-Ray Tomography. Microsc. Microanal. 2012;18:390-8.

27. Jaiswal Y, Weber D, Yerke A, Xue Y, Lehman D, Williams T, Xiao T, et al. A substitute variety for agronomically and medicinally important Serenoa repens (saw palmetto). Sci. Rep. 2019;9:4709.

28. Van Noordwijk M, Brouwer G. Quantification of air-filled root porosity: A comparison of two methods. Plant and Soil. 1988;111:255-8.

29. Wongs-Aree C, Noichinda S. Glycolysis Fermentative By-Products and Secondary Metabolites Involved in Plant Adaptation under Hypoxia during Pre- and Postharvest. In: Kusal K. D.; Biradar MS, editor. Hypoxia and Anoxia. London, UK: Intechopen; 2018. p. 59-72.

30. Piekarska-Stachowiak A, Szymanowska-Pulka J, Potocka I, Lipowczan M. Topological traits of a cellular pattern versus growth rate anisotropy in radish roots. Protoplasma. 2019;256:1037-49.

31. Atkinson JA, Rasmussen A, Traini R, Voss U, Sturrock C, Mooney SJ, Wells DM, et al. Branching out in roots: uncovering form, function, and regulation. Plant Physiol. 2014;166:538-50.

32. Herremans E, Verboven, P., Verlinden, B.E., Cantre, D., Abera, M., Wevers, M., Nicolaï, B.M. Automatic analysis of the 3-D microstructure of fruit parenchyma tissue using X-ray micro-CT explains differences in aeration. BMC Plant Biol. 2015;15:1-14.

33. Barry JA, Groseclose MR, Robichaud G, Castellino S, Muddiman DC. Assessing drug and metabolite detection in liver tissue by UV-MALDI and IR-MALDESI mass spectrometry imaging coupled to FT-ICR MS. Int. J. Mass Spectrom. 2015;377:448-155.

34. Rosen EP, Bokhart MT, Nazari M, Muddiman DC. Influence of C-Trap Ion Accumulation Time on the Detectability of Analytes in IR-MALDESI MSI. Anal. Chem. 2015;87:10483-90.

35. Nazari M, Ekelof M, Khodjaniyazova S, Elsen NL, Williams JD, Muddiman DC. Direct screening of enzyme activity using infrared matrix-assisted laser desorption electrospray ionization. Rapid Commun. Mass Spectrom. 2017;31:1868-74.

36. Baum SS, Hill R, Rommelspacher H. Effect of kava extract and individual kavapyrones on neurotransmitter levels in the nucleus accumbens of rats. Prog. Neuro-Psychopharmacol. Biolog. Psychiatry. 1998;22:1105-20.

37. Walden J, Von Wegerer J, Winter U, Berger M, Grunze H. Effects of kawain and dihydromethysticin on field potential changes in the hippocampus. Prog. Neuro-Psychopharmacol. Biol. Psychiatry. 1997;21:697-706.
38. Smith KK, Dharmaratne HR, Feltenstein MW, Broom SL, Roach JT, Nanayakkara NP, Khan IA, et al. Anxiolytic effects of kava extract and kavalactones in the chick social separation-stress paradigm. Psychopharmacology (Berl). 2001;155:86-90.
39. Lèvesque VLaJ. The origin and distribution of kava (Piper methysticum Forst. f., Piperaceae): a phytochemical approach. Allertonia. 1989;5:223-81.
40. Cairney S, Maruff P, Clough AR, Collie A, Currie J, Currie BJ. Saccade and cognitive impairment associated with kava intoxication. Hum. Psychopharmacol. 2003;18:525-33.
41. Pollock NJ. Sustainability of the kava trade. Contemp. Pacific. 2009;21:265-97.
42. Jaiswal Y, Weber D, Yerke A, Xue Y, Lehman D, Williams T, Xiao T, et al. A substitute variety for agronomically and medicinally important Serenoa repens (saw palmetto). Sci Rep. 2019;9:4709.
43. Snijders MLH, Zajec M, Walter LAJ, de Louw R, Oomen MHA, Arshad S, van den Bosch TPP, et al. Cryo-Gel embedding compound for renal biopsy biobanking. Sci Rep. 2019;9:15250.
44. Khodjaniyazova S, Nazari M, Garrard KP, Matos MPV, Jackson GP, Muddiman DC. Characterization of the Spectral Accuracy of an Orbitrap Mass Analyzer Using Isotope Ratio Mass Spectrometry. Anal. Chem. 2018;90:1897-906.
45. Tarbah F, Mahler H, Kardel B, Weinmann W, Hafner D, Daldrup T. Kinetics of kavain and its metabolites after oral application. J. Chromatogr. B. 2003;5:115-30.
46. Wang Y, Eans SO, Stacy HM, Narayanapillai SC, Sharma A, Fujioka N, Haddad L, et al. A stable isotope dilution tandem mass spectrometry method of major kavalactones and its applications. PLoS One. 2018;13:e0197940.
47. Smith RM, Thakrar H, Arowolo TA, Shafi AA. High-performance liquid chromatography of kava lactones from piper methysticum. J. Chromatogr. A. 1984;283:303-8.
48. Bokhart MT, Nazari M, Garrard KP, Muddiman DC. MSiReader v1.0: Evolving Open-Source Mass Spectrometry Imaging Software for Targeted and Untargeted Analyses. J Am Soc Mass Spectrom. 2018;29:8-16.

49. Kava 3D Imaging and Metabolomics project page. https://github.com/palomnyk/kava_3D_imaging_and_metabolomics.

50. MetaboLights Database. https://www.ebi.ac.uk/metabolights/.

51. Root stem crown comparison Project Homepage. https://github.com/palomnyk/root_stem_crown_comparison.

52. Jaiswal YS; Yerke AM; Bagley MC; Ekelöf M; Weber D; Haddad D; Fodor AA; Muddiman DC; Williams LL: Supporting data for "3D imaging and metabolomic profiling reveal higher neuroactive kavalactone contents in lateral roots and crown root peels of Piper methysticum (Kava)" GigaScience Database. 2020. http://dx.doi.org/10.5524/100784.

Figure legends

Figure 1. Specific kava roots and stems tissues selected for the study, and the applied statistical models. Two mixed linear models were made based on the selection of the various parts of *P. methysticum*. **CRP, CNP,** and **LR** denote the crown root peel, crown with no peel, and lateral roots. **SP** and **SNP** denote stem peels and stems with no peels, respectively. The plant parts are divided into stem, crown roots and lateral roots, thus there was a model for these structural locations with levels: stem, lateral root, and crown root. Finally the stems and crown roots were divided into “peel” or “no peel” and we used these, with the lateral roots, as the levels for the model with a term for tissue (Stems and crown roots “with peels” samples were excluded from the mixed linear model).

Figure 2. GC-MS Total Ion chromatograms of various parts of *P. methysticum* roots. **A**, peel of crown roots (**CRP**), **B**, crown root with no peel (**CNP**), **C**, crown root with peel (**CWP**), and **D**, lateral roots (**LR**). Some representative kavalactones and other compounds found in the extracts are denoted as δ-Cadinol (**c**), dihydromethysticin (**d**), α-epi-7-epi-5-Eudesmol (**e**), kavain (**k**) and desmethoxyyangonin (**y**).

Figure 3. PCA and Box plots for GC-MS and LC-MS analysis of kava roots and stems. PCA plots of **(A)** GC-MS, **(B)** LC-MS positive and **(C)** LC-MS negative mode indicate a clear distinction between roots (green) and stems (purple) for each dataset. PCA1 separated the crown roots, lateral roots and stems with adjusted p-values of the pairwise Student’s t-test as [3.43E-05, 0.0003458] in **(A)**, [0.000475, 0.00257] in **(B)**, and [0.000585, 0.00274] in **(C)**, respectively.

PCA1 also separates both crown roots (triangles) and lateral roots (circles) from stem (squares). The mixed linear model of the adjusted p-values for PCA1 and PCA2 of GC-MS, LC-MS positive, LC-MS negative mode are 2.037E-06, 0.00206, and 2.137E-05, respectively. The axes of each plot show the principal components and the percentage of the variance that they explain rounded to the nearest percent.

(D) kavain, **(E)** desmethoxyyangonin and **(F)** dihydromethysticin indicated in the boxplots, have adjusted p-values of 7.05E-06, 3.40E-06, and 7.05E-06 in the mixed linear model for different tissue types. Black bars below box plots indicate statistical significance from adjusted p-values from pairwise Student's t-test where '*' indicates $P \leq 0.05$, '**' indicates $P \leq 0.0$, and '***' indicates $P \leq 0.001$. Significant differences were observed between roots (green) and stems (purple). **CRP, CNP,** and **LR** denote the crown root peel, crown with no peel, and lateral roots. **SP** and **SNP** denote stem peels and stems with no peels, respectively. These plots exclude the whole stem and crown sections (**SWP** and **CWP**).

Figure 4. X-ray µCT images of crown and lateral roots of *P. methysticum*.
(A-H) and **(I-P)** denote images of 3D surface reconstructions based on µCT data of crown and lateral roots, respectively.
(A) and **(B)** are the segmented air-filled spaces in parenchyma and the cork (peel). **(C)** represents the overlay of the cork and air-filled regions in parenchyma of the crown roots. **(D)** 3D rendering image of the whole section of crown root. **(E)** and **(F)** represent the transverse view images of crown roots in 3D rendering and grey scale, respectively. **(G)** and **(H)** represent the longitudinal view images of crown roots in grey scale and 3D rendering, respectively.
(I) and **(J)** are the segmented tissues of lateral roots, comprising of air-filled spaces in parenchyma and the cork (peel), respectively. **(K)** an overlay of the segmented regions of lateral roots including cork and air-filled regions in parenchyma. **(L)** 3D rendering image of the whole section of lateral roots. **(M)** and **(N)** represent the transverse view of lateral roots in 3D rendering and grey scale. **(O)** and **(P)** represent the longitudinal view images of lateral roots in grey scale and 3D rendering. The sections **a**, **b**, **c** and **d** represent cork, cortex, parenchyma and air-filled spaces, respectively.

Figure 5. IR-MALDESI ion abundance heatmaps for various constituents in different parts of *P. methysticum*. (**A**) Distribution of kavain (K), 7,8-Dihydro-5,6-dehydrokawain (5,6-DDK): *m/z* 231.1016, (**B**) Optical images of various parts (**C**) Distribution of desmethoxyyangonin (DMY): *m/z* 229.0859 (**D**) Distribution of dihydromethysticin (DHM) *m/z* 277.107. Images in **a-c**, **d-f** and **g-i**, indicate lateral roots, crown roots and stem sample images, respectively.

Figure 6. MS-MS overlay chromatograms and fragmentation pattern of kavain obtained in Parallel Reaction Monitoring (PRM) MALDESI. (A) represent the overlay chromatograms of standard compound, **(B)** represents the fragmentation pattern of kavain, **(C)** and **(D)** represent the overlay chromatograms of kavain on crown root and stem tissues, respectively. The y-axis is

abundance of the precursor (top) and MS/MS fragments (bottom) of the kavain m/z (231.1016). The x-axis indicates time (min).

Tables

Table 1. Results of quantitative analysis of selected kava lactones by GC-MS in various plant parts and separated tissues of *P. methysticum*

Sample	Kavain (mg/g ± SD)	Dihydromethysticin (mg/g ± SD)	Desmethoxyyangonin (mg/g ± SD)
CWP	0.425 ± 0.235	1.689 ± 0.862	0.355 ± 0.138
LR	2.003 ± 0.615	2.842 ± 0.748	0.901 ± 0.182
SWP	0.042 ±0.012	0.290 ±0.110	0.080 ±0.015
CRP	0.987 ± 0.235	1.875 ± 0.467	0.660 ± 0.146
CNP	0.922 ± 0.187	2.055 ± 0.445	0.516 ± 0.088
SP	0.069 ± 0.021	0.500 ± 0.204	0.161 ± 0.044
SNP	0.128 ± 0.027	0.356 ± 0.067	0.165 ± 0.029

CWP, **LR** and **SWP** denote the whole crown roots, lateral roots and stems of *P. methysticum*, respectively. **CRP, CNP, SP** and **SNP denote** the crown root peel, crown root with no peel, stem peels and stems with no peels, respectively. The concentrations of kavalactones in selected plant parts were calculated on a dry weight basis.

Figure 1

Click here to access/download;Figure;Figure 1.tif

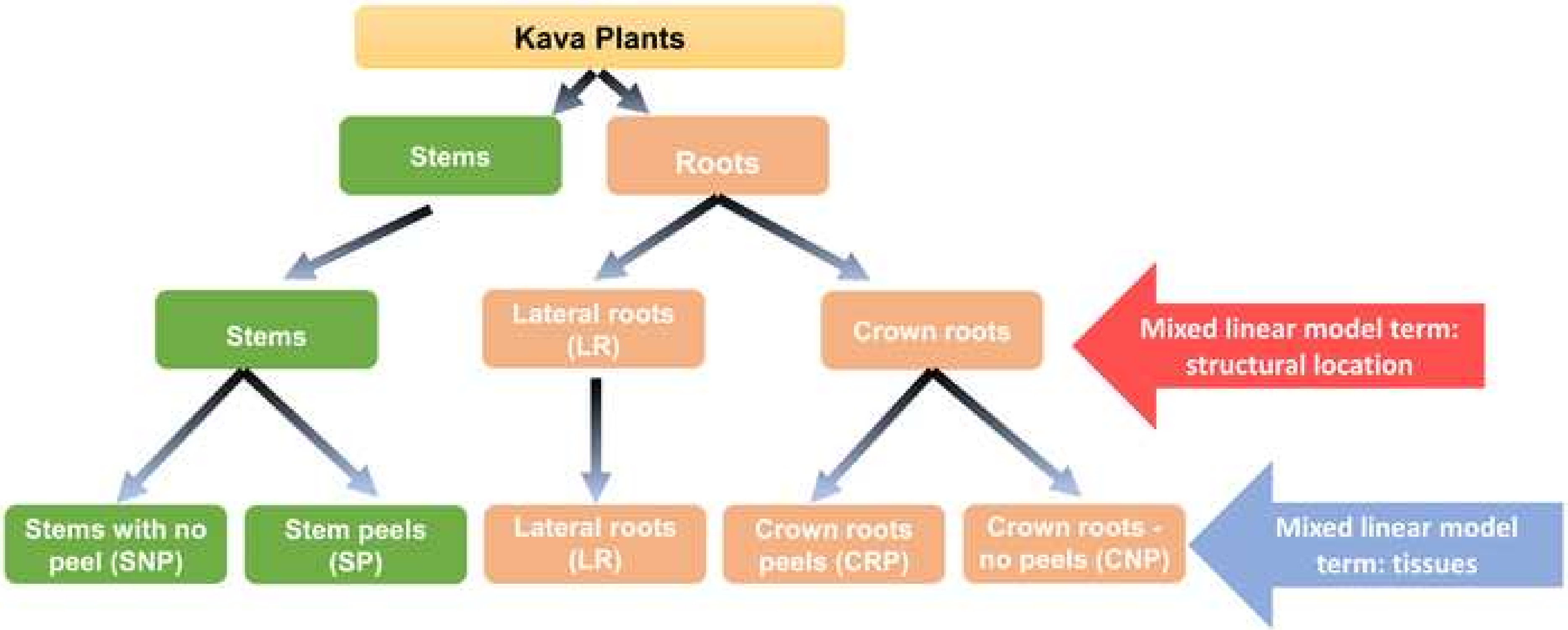

Figure 2

Click here to access/download;Figure;Figure 2.tif

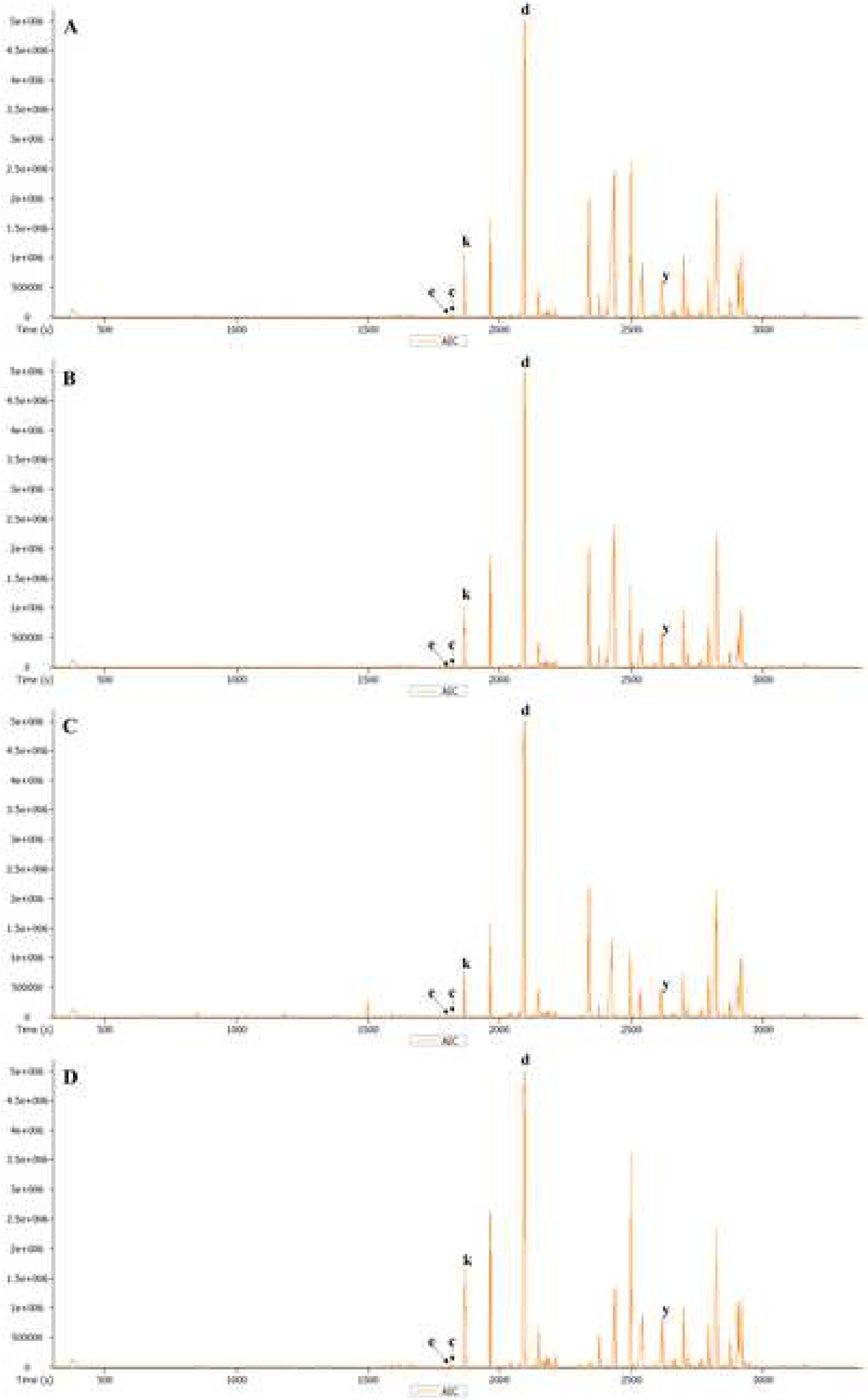

Figure 3

Click here to access/download;Figure;Figure 3.tif

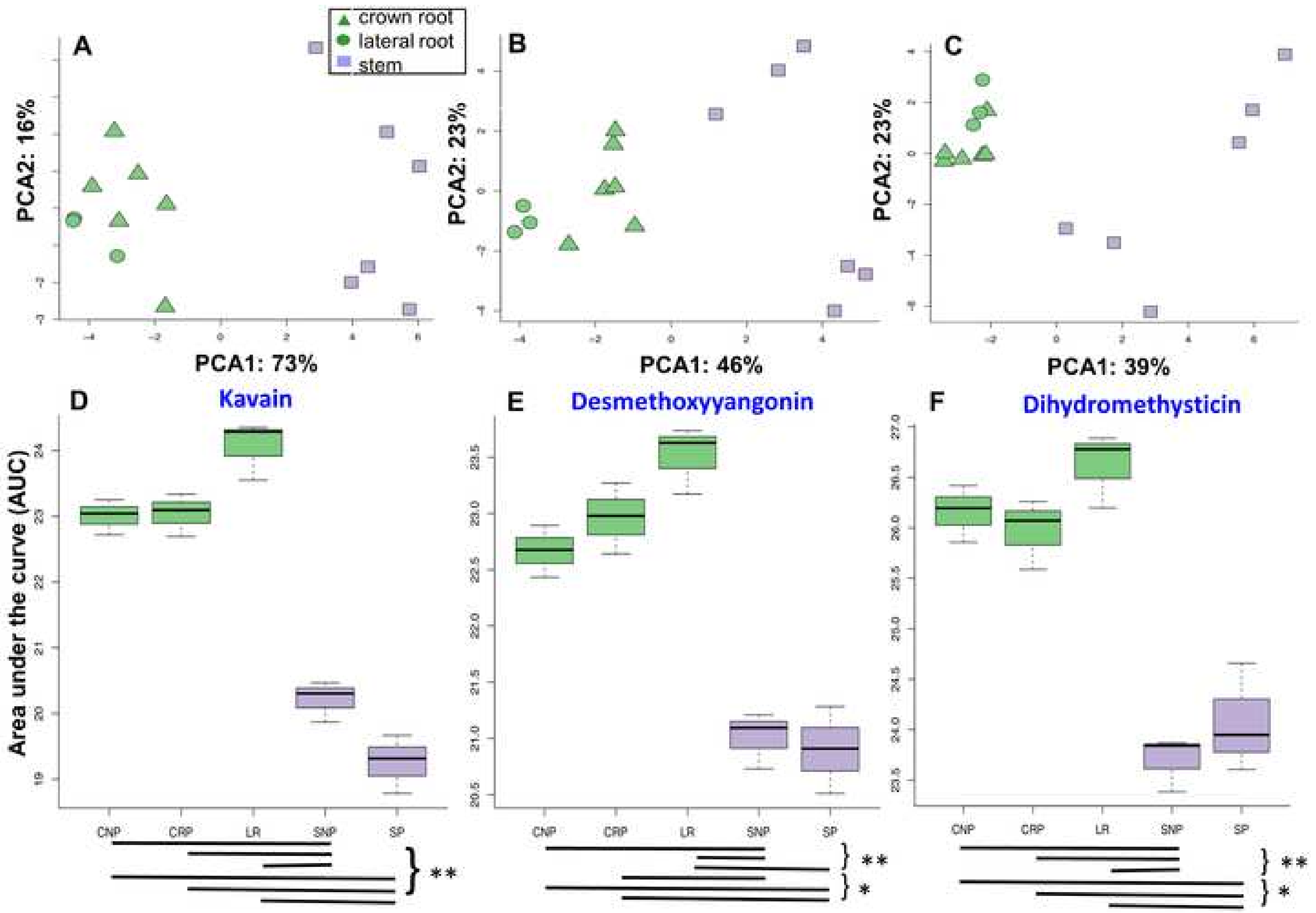

Figure 4

Click here to access/download;Figure;Figure 4.tif

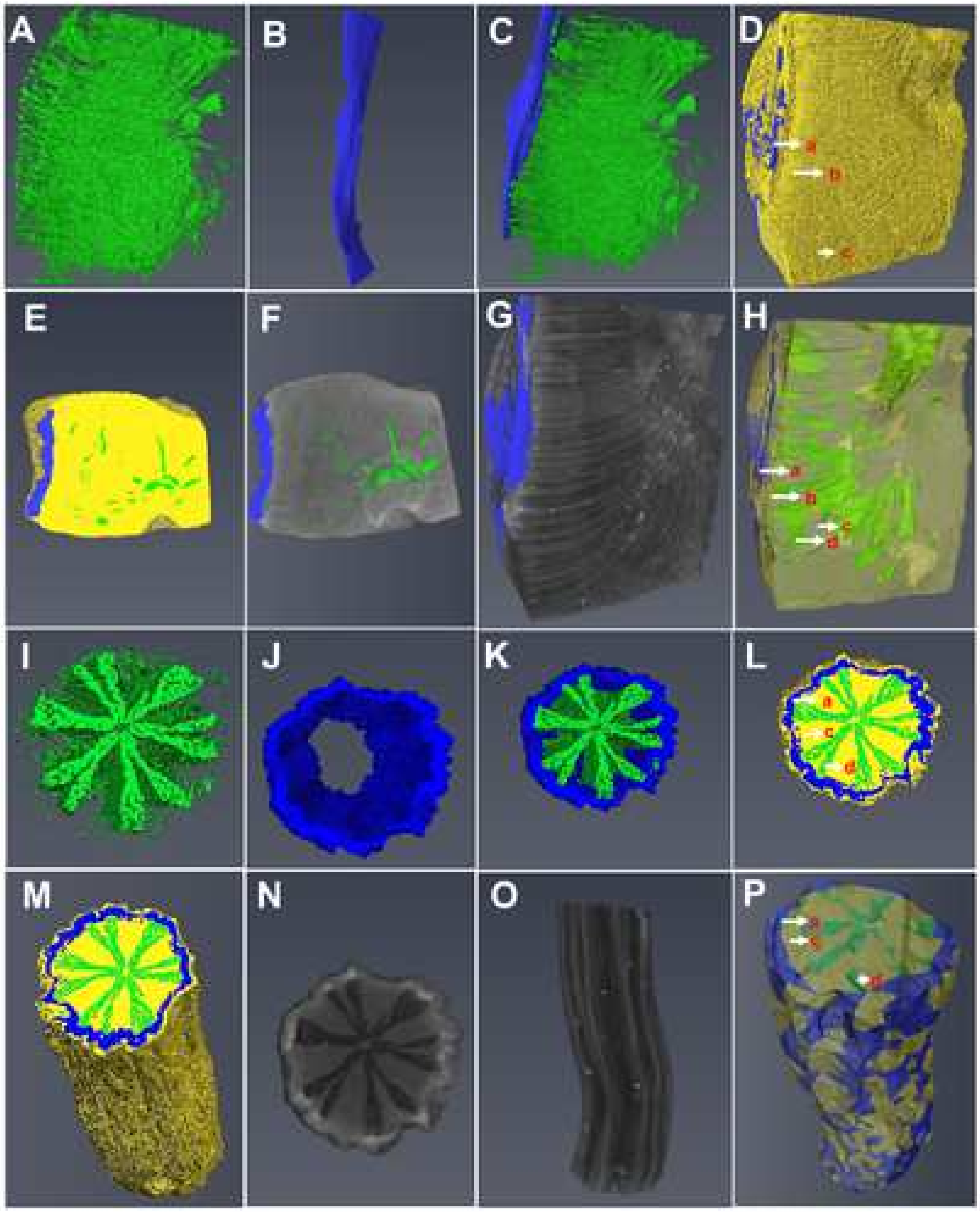

Figure 5

Click here to access/download;Figure;Figure 5.tif

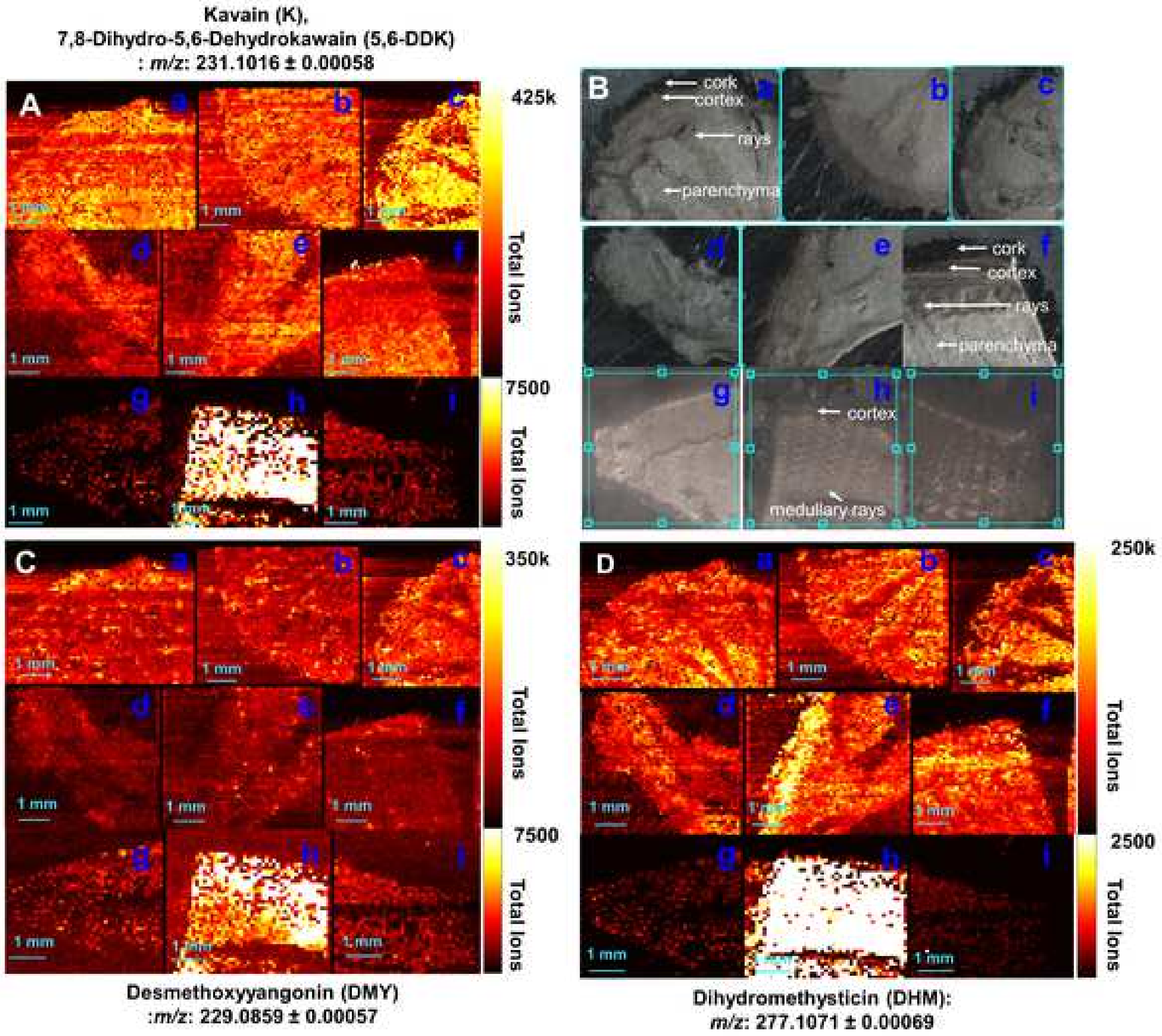

Figure 6

Click here to access/download;Figure;Figure 6.tif

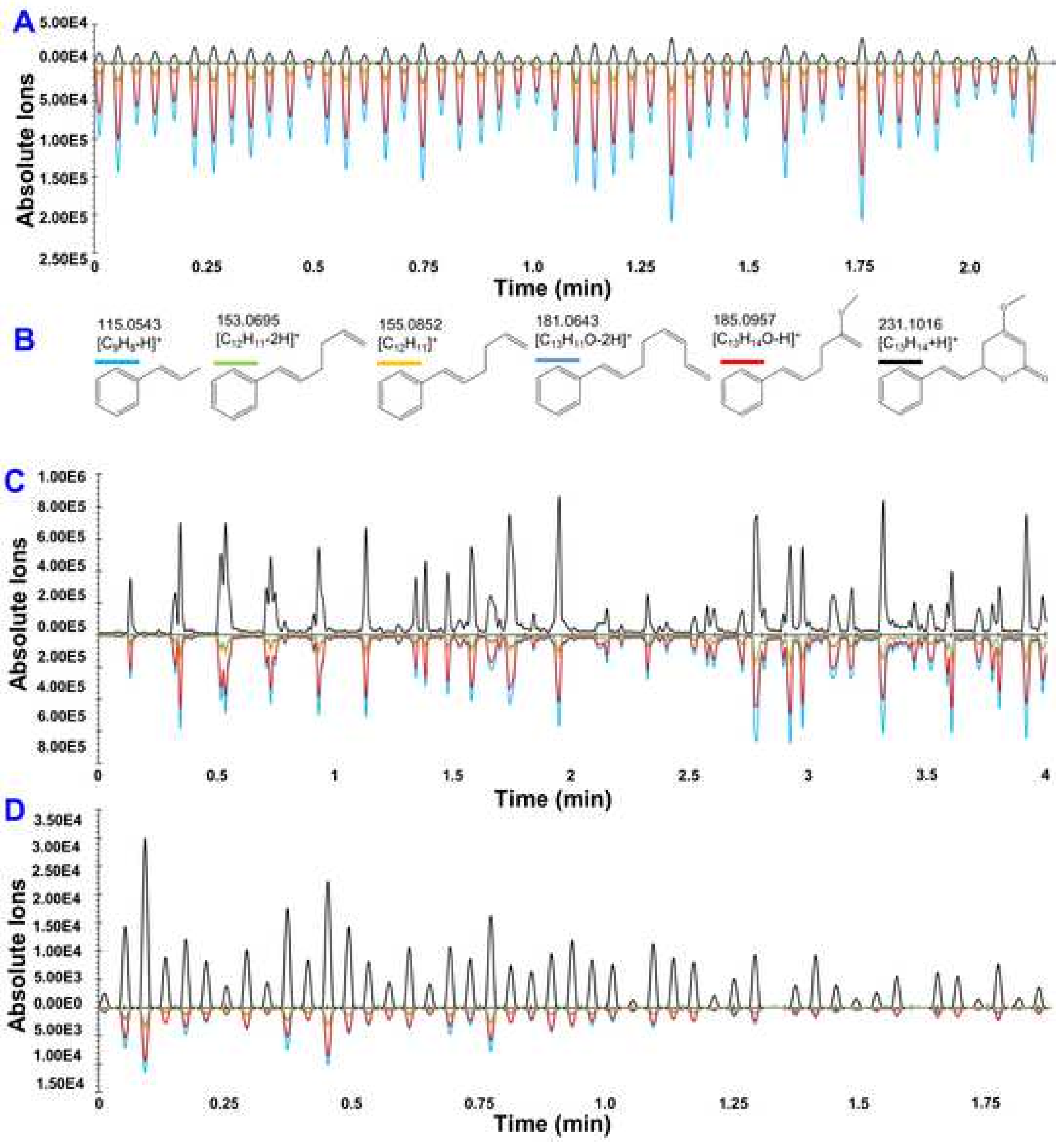

Click here to access/download
Supplementary Material
Additional Files - revised.docx

Personal Cover and response letter
Click here to access/download;Personal Cover;Cover letter-Giga Science - Revision 2.docx

Date: 08.05.2020

To,
The Editor
GigaScience, Oxford Academic Press.

Sub: Revised article submission (MS. Ref.# GIGA-D-20-00163)

Title: 3D imaging and metabolomic profiling reveal higher neuroactive kavalactone contents in lateral roots and crown root peels of *Piper methysticum* (Kava)

Dear Editor,

Please find enclosed our revised research manuscript for further processing in *GigaScience*. We sincerely thank the editor for the positive feedback and interest in publication of our manuscript.

We appreciate the constructive critiques provided by the reviewers and the appreciation for our research work. The suggestions were helpful in improving the quality of our manuscript.

We have adopted suggestions from the reviewers and modified the manuscript accordingly. Specifically, (i) Figures and their legends have been modified, (ii) suggested references were deleted and, (iii) additional information was provided in the methodology section. A detailed point-to point response letter to the reviewers' comments, follows this cover letter. The changes mentioned above in the manuscript are marked in red font. The manuscript is suitably improved, and we hope it will be found satisfactory for further evaluation.

The findings of the study presented in the article remain unchanged after revision. Publication in *GigaScience* will provide an effective impact on the wide range of readership, for supportive action towards quality control and safe use of Kava.

Thank you again for favourable consideration of our revised article.

Best regards,

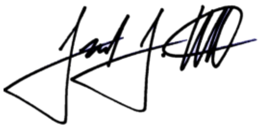

Leonard L. Williams, PhD, MBA
Center for Excellence in Post Harvest Technologies
NC A&T State University, 500 Laureate Way,
Kannapolis, NC 28081, USA.
Phone: (704) 250-5700 ext. 5703, Fax: (704) 250-5709
Email: llw@ncat.edu

Response letter for Manuscript ref. no: GIGA-D-20-00163

The authors sincerely appreciate the time and effort invested by the reviewers in providing their valuable inputs. These suggestions have improved our manuscript significantly.

In the revised manuscript, all the comments made by the reviewers have been suitably addressed. Specifically, (i) Figures and figure legends have been corrected (ii) tables have been modified, and (iii) certain references have been deleted, as recommended by the reviewers.

A detailed point-to-point response for each of reviewers' comments and indications of following it, are detailed below. We hope that the reviewers will find the revised manuscript satisfactory for further evaluation.

Queries/critiques are numbered and in blue Times Roman font.

Responses follow in black Times Roman.

Revised text from the manuscript is indicated in red Times Roman.

Reviewer reports:

Reviewer #1: The authors addressed most of my concerns. However, I found some issues in the revised manuscript that should be fixed prior to publication.

1. The authors added additional references to introduction, but the referencing is quite confusing. Specifically, this sentence: "Novel dimeric kavalactones, namley diyangonins (A-C) have also reported to be isolated from kava roots [10-15]." References 10-13 have nothing to do with dimeric kavalactones.

Response: The authors thank the reviewer for pointing out this error. As per the recommendations, the non-relevant references have been deleted from the specified sentence.

Section: Manuscript - Introduction
Please refer: Line numbers 54-55, page 3 - Reference nos. 10, 11
Text: "Novel dimeric kavalactones, ………….. to be isolated from kava roots [10, 11]"

2. The authors claim they deposited their raw data into the Metabolights repository, but no such information (or deposition ID) is mentioned in the "Availability of Data and Materials" section.

Response: As per the reviewer's suggestion, the Metabolights repository/deposition ID is mentioned in the "Availability of supporting data and materials" section.

Section: Manuscript: Availability of supporting data and materials
Please refer: Line numbers 464-466, page 22
Text: "Metabolomics data have been deposited ……….with the identifier MTBLS1485".

3. Fig.5: The mass of 7,8-dihydro-5-hydroxykavain (5-DHK) ion is not 231.1016 m/z. Furthermore, p-hydroxykavain is a known metabolite of kavain in liver (doi:10.1016/S1570-0232(03)00046-1), but has it ever been reported in the plant?

Response: We thank the reviewer for pointing out these important errors. The necessary modifications have been carried out in Fig.5, and these two compounds have been removed from the figure.

Please refer: Figure 5 legend, Page 31, Line nos: 685-689

Text: Legend for Figure 5 – "IR-MALDESI ion abundance …….. (**A**) Distribution of kavain (K), 7,8-Dihydro-5,6-dehydrokawain (5,6-DDK): *m/z* 231.1016".

4. I do not understand Figure 6 at all. The legend says it contains MS/MS spectra, but it clearly doesn't contain any spectra (mass spectrum = m/z vs intensity). There is no label or scale for X axis! In their response the authors explained that "The yaxis is abundance of the precursor (top) and MS/MS fragments (bottom) of the kavain m/z (231.1016)." but they didn't add this description into the legend. I am missing clear description how the MS/MS experiments were performed (precursor selection, collision energy?).

Response: We thank the reviewer for pointing out this important error.

Our apologies for the confusion. During revisions we meant to change 'spectra' into 'chromatograms'. The X-axis in (A) is an arbitrary length of time that the standards were analyzed. In (C) and (D) the time scale is just from the beginning of the experiment to the end across tissue.

In Parallel Reaction Monitoring (PRM) mode in Orbitrap-based Thermo MS, every nth scan is dedicated to a full MS scan across the entire m/z range of interest, or one or another of select m/z of interest. In this particular case the 1st scan was a full MS scan from 120-480 m/z, the next was an MS/MS scan of 231.1016 with an isolation window of 1.5 m/z fragmented with NCE (normalized collision energy) = 30, the 3rd was an MS/MS scan of 277.1071 with NCE = 20, and the 4th was an MS/MS scan of 229.0859 with NCE = 35, then the cycle repeated. Each NCE value was optimized in unpublished experiments. We have attached the updated figure to make this more clear.

As per the reviewer's suggestions, the requested description is now added in the manuscript's "Materials and Methods" section and the figure legend is now modified.

Please refer: Page 20, Line nos: 416-422 and Figure 6.

Text: In Parallel Reaction Monitoring (PRM) mode in ……………………was optimized in unpublished experiments (**Figure. 6**).

5. (a) Supplementary figures S2 to S5 need more careful preparation. The legend of Fig. S2 points to images a-c, d-f, and g-i,which are not indicated in the figure.
(b) It is unclear why Fig S2 shows 3 kavalactones, Fig. S3.1 shows 15 kavalactones, Fig.S3.2 shows 10 kavalactones, and Fig. S4 shows 20 kavalactones.
(c) Dihydrokawain is labeled as 233.1172 m/z in Fig. S3.1 but 215.1066 m/z in Fig. S3.2, why?
(d)The inclusion of p-hydroxykavain is questionable (see above).
(e) Fig. S5 suffers again from the superposition of some signals (231.1016 m/z is not only kavain).

Responses: We thank the reviewer for queries. Following are the specific responses:

(a) We apologize for the oversights in Supplementary Figure S2 and the confusion they have caused. The legend and figure of Supplementary Figure S2 have been revised, as per the reviewer's sugegstions.
Please refer: Additional files: Page no-53-56, Supplementary Figure S2.

(b) The focus of the study has been much more oriented towards the 3 kavalactones shown in Figure S2, hence its own figure here. Figure S3.1 and S3.2 go together but were split up for clearer presentation as a Figure with them 5 sub-figure wide would make the images too small. Their purposes were to show that we analyzed more kavalactones than just the ones of focus shown in the main bodies and figures of the paper in the crown roots, and then S4 has the same purpose, but for lateral roots. Supplementary Figures S3.1 and S3.2 show constituents from crown roots and Supplementary Figure S4 shows constituents from lateral roots.

(c) Dihydrokawain was identified both times but the 233.1172 *m/z* was its proton adduct peak, and the 215.1066 is the proton adduct with water loss. The water loss proton adduct is a commonly identifiable peak in many of our studies.

(d) As suggested by the reviewers, all occurrences of p-hydroxykavain in figures and text have been deleted.

(e) Yes, not all the images we provide in the supplementary images are as high quality as others. Since we had performed MS/MS on kava roots and stems and compared it to the standard, the identification of 231.1016 m/z as kavain is a solid one. For other kava lactones putatively identified without MS/MS, we have to rely on a combination of biological and spectral verification. Supplementary Figures S2-S4 have been suitably revised.

6. The methods section needs a clear explanation how the metabolites in Sup. Tables S1 and S2 were identified, and confidence of the identification should be noted in the tables, see Blaženović, I., Kind, T., Ji, J. & Fiehn, O. Software Tools and Approaches for Compound Identification of LC-MS/MS Data in Metabolomics. Metabolites 8, 31 (2018).

Response: A clear explanation of how metabolites were identified in Table S1 and S2 is provided in the methods section. The confidence of identification are now mentioned in Tables S1 and S2.

(a) Please refer: Manuscript: Methodology, Page 16, Line nos. 327-332
Text: "Baseline smoothing, peak picking, automated ……. were used for metabolite annotation."

(b) Please refer: Manuscript: Methodology, Page 15, Line nos. 302-306
Text: "The resulting data …………..specific molecular ions and masses".

(c) Please refer: Supplementary Tables S1 and S2 (Additional files: page 35-41).

We thank the reviewer once again for their efforts in providing us these important suggestions.

Reviewer #2: The authors have made substantial changes in the manuscript and have addressed all the issues that were raised for the previous version. The manuscript now reads well and there are no further comments or questions for the latest version.

Responses: The authors thank the reviewer for considering out manuscript favourably, and for the time and effort in providing us valuable suggestions to improve it.

Reviewer #3: I am satisfied how authors addressed all my questions with enough supporting data. I have one minor comment that can significantly improve citation of your manuscript:

1. Please provide more detailed information on sectioning of kava roots. What was duration of soaking? Have you used some polymeric material to embed roots? There is a big challenge to section roots of woody plants without disturbing their morphology (without chemical fixation) and your optical images shows that you achieved that!

Response: We sincerely thank the reviewer for a positive approval of our manuscript and appreciating our work.

As per your recommendations, we have provided the requested information in the manuscript.

Please refer: Manuscript: Methods, Page: 18, Line nos. 364-371
Text: "The samples for cryo-sectioning were prepared ……..a polymeric gel upon freezing [43]".

We thank the reviewer once again, for the efforts in providing us constructive suggestions and helping us improve our manuscript further.
