# Supplementary material for: 3D Imaging and metabolomic profiling reveal higher neuroactive kavalactone contents in lateral roots and crown root peels of Piper methysticum (kava)
Source: Gigascience. 2020 Sep 22;9(9):giaa096. doi: 10.1093/gigascience/giaa096 (PMC7507772; doi:10.1093/gigascience/giaa096)
Supplement: giaa096_Supplemental_File [file giaa096_supplemental_file.docx]

**3D imaging and metabolomic profiling reveal higher**

**neuroactive kavalactone contents in lateral roots**

**and crown root peels of *Piper methysticum* (Kava)**

Yogini S. Jaiswal*^1,7^, Aaron M. Yerke^2,7^, Michael C. Bagley^3^, Måns Ekelöf^3^, Daniel Weber^4^, Daniel Haddad^4,6^, Anthony Fodor^2,6^, David C. Muddiman^3,5,6^, Leonard L. Williams*^1^

*^1^Center for Excellence in Post-Harvest Technologies, North Carolina Agricultural and Technical State University, The North Carolina Research Campus, 500 Laureate Way, Kannapolis, NC-28081, USA.* ***Emails:*** [*llw@ncat.edu*](mailto:llw@ncat.edu)*,* [*yoginijaiswal@gmail.com*](mailto:yoginijaiswal@gmail.com)

*^2^Department of Bioinformatics and Genomics, University of North Carolina at Charlotte, Charlotte, North Carolina-28223, USA.* ***Emails:*** [*afodor@uncc.edu*](mailto:afodor@uncc.edu)*,* [*amyerke@uncc.edu*](mailto:amyerke@uncc.edu)

*^3^Department of Chemistry, North Carolina State University, Raleigh, NC-27695, USA.* ***Emails:*** [*mcbagley@ncsu.edu*](mailto:mcbagley@ncsu.edu)*,* [*moekeloe@ncsu.edu*](mailto:moekeloe@ncsu.edu)*,* [*dcmuddim@ncsu.edu*](mailto:dcmuddim@ncsu.edu)

*^4^ Fraunhofer Development Centre X-Ray Technology EZRT, Division of Fraunhofer Institute for Integrated Circuits IIS, Department Magnetic Resonance and X-Ray Imaging MRB, Am Hubland D-97074 Wurzburg, Germany.* ***Emails:*** [*Daniel.Haddad@physik.uni-wuerzburg.de*](mailto:Daniel.Haddad@physik.uni-wuerzburg.de)*,* [*weber@mr-bavaria.de*](mailto:weber@mr-bavaria.de)

*^5^Molecular Education, Technology and Research Innovation Center (METRIC), North Carolina State University, Raleigh, NC-27695, USA.* ***Email:*** [*dcmuddim@ncsu.edu*](mailto:dcmuddim@ncsu.edu)

*^6^*Co-Senior authors

*^7^*These authors contributed equally to this article

***Correspondence:** llw@ncat.edu, [yoginijaiswal@gmail.com](mailto:yoginijaiswal@gmail.com)

**Additional information**

| **Table of contents** | |
| --- | --- |
| **Table S1** | Metabolites identified in roots of *P.methysticum* by GC-MS analysis. |
| **Table S2** | Metabolites identified in common in various parts of *P.methysticum* by LC-MS analysis. |
| **Table S3** | Secondary metabolites in various tissues of *P.methysticum* identified by LC/MS analysis |
| **Table S4** | P-values of mixed linear model with GC/MS data |
| **Table S5** | 3D morphological and geometric descriptors of roots of *P. methysticum* |
| **Fig. S1** | Pictorial representations of plant parts of *P.methysticum* and their μCT imaging and sectional views. |
| **Fig. S2** | IR-MALDESI ion abundance heatmaps for various constituents in different parts of *P. methysticum.* |
| **Fig. S3.1** | **IR-MALDESI ion abundance heatmaps for various constituents in crown roots of *P. methysticum*** |
| **Fig. S3.2** | **IR-MALDESI ion abundance heatmaps of some constituents identified in crown roots of *P. methysticum*** |
| **Fig. S4** | **IR-MALDESI ion abundance heatmaps for various constituents in lateral roots of *P. methysticum*** |
| **Fig. S5** | Isotope Count Heatmap for kavalactones on various tissues of *P.methysticum.* |
|  |  |

**Supplementary Table S1:**

Metabolites identified in roots of *P.methysticum* by GC-MS analysis.

| **Putative Compound Name** | **Formula** | **Theoretical Molecular Weight** | | **Quantific-ation masses** | **Retention Time** | | | **RI (iu)** | **CRP** | | | **CNP** | **CWP** | **LR** | | **SWP** | | **SP** | **SNP** | **Identification confidence level (A-B)*** |
| --- | --- | --- | --- | --- | --- | --- | --- | --- | --- | --- | --- | --- | --- | --- | --- | --- | --- | --- | --- | --- |
| **Kava lactones** | | | | | | | | | | | | | | | | | | | |  |
| Kavain | C_14_H_14_O_3_ | 230.263 | 186 | | | 1862.06 | 1934 | | | + | + | | + | + | + | | + | | + | A |
| Dihydromethysticin | C_15_H_16_O_5_ | 276.288 | 135 | | | 2092.58 | 2270 | | | + | + | | + | + | + | | + | | + | A |
| Dihydro-5,6-dehydrokawain | C_14_H_14_O_3_ | 230.263 | 91 | | | 2376.47 | 1937 | | | + | + | | + | + | + | | + | | + | B |
| Dihydrokavain | C_14_H_16_O_3_ | 232.279 | 127 | | | 2429.84 | 1926 | | | + | + | | + | + | + | | + | | + | B |
| Desmethoxyyangonin | C_14_H_12_O_3_ | 228.247 | 228 | | | 2614.18 | 1945 | | | + | + | | + | + | + | | + | | + | A |
| Yangonin | C_15_H_14_O_4_ | 258.273 | 258 | | | 2698.59 | 2115 | | | + | + | | + | + | + | | + | | + | B |
| Tetrahydro-11-methoxyiangonin | C_16_H_20_O_5_ | 292.331 | 151 | | | 2854.54 | 2304 | | | + | + | | + | + | + | | + | | + | B |
| **Dihydrochalcones** | | | | | | | | | | | | | | | | | | | |  |
| Flavokavain B | C_17_H_16_O_4_ | 284.311 | 207 | | | 2876.12 | 2408 | | | + | + | | + | + | + | | + | | + | B |
| Flavokawain A | C_18_H_18_O_5_ | 314.337 | 314 | | | 3159.63 | 2597 | | | + | + | | + | + | + | | + | | + | B |
| Flavokawain C | C_17_H_16_O_5_ | 300.31 | 193 | | | 2663.7 | 2440 | | | + | + | | + | + | + | | + | | + | B |
| **Non-Kava lactones** | | | | | | | | | | | | | | | | | | | |  |
| Palmitic Acid | C_16_H_32_O_2_ | 256.430 | 73 | | | 2201.97 | 1968 | | | + | + | | + | + | - | | + | | + | B |
| Pyrrolidine, 1-(m-methoxycinnamoyl)- | C_14_H_17_NO_2_ | 231.295 | 161 | | | 2619.86 | 1918 | | | + | + | | + | + | + | | + | | + | B |
| Benzenepropanal | C_9_H_10_O | 134.178 | 91 | | | 1038.03 | 1181 | | | + | + | | + | + | + | | - | | - | B |
| Hydrocinnamic acid | C_9_H_10_O_2_ | 150.177 | 91 | | | 1331 | 1349 | | | + | + | | + | + | - | | + | | - | B |
| Hedycaryol | C_15_H_26_O | 222.372 | 59 | | | 1670.16 | 1694 | | | + | + | | + | + | + | | + | | + | B |
| δ-Cadinol | C_15_H_26_O | 222.372 | 59 | | | 1822.7 | 1598 | | | + | + | | + | + | - | | - | | - | B |
| α-epi-7-epi-5-Eudesmol | C_15_H_26_O | 222.372 | 119 | | | 1809.07 | 1580 | | | + | + | | + | + | - | | - | | - | B |
| 1,10-Undecadiene | C_11_H_20_ | 152.281 | 67 | | | 2390.47 | 1095 | | | - | - | | + | - | + | | + | | + | B |
| Linoelaidic acid | C_18_H_32_O_2_ | 280.452 | 67 | | | 2402.59 | 2183 | | | + | + | | + | + | - | | + | | + | B |
| 13-Tetradece-11-yn-1-ol | C_14_H_24_O | 208.345 | 79 | | | 2403.72 | 1663 | | | + | + | | + | + | - | | + | | + | B |
| Bornyl cinnamate | C_19_H_24_O_2_ | 284.399 | 131 | | | 2494.95 | 2058 | | | + | + | | + | + | + | | + | | + | B |
| Pipermethystine | C_16_H_17_NO_4_ | 287.315 | 104 | | | 2511.22 | 2358 | | | + | + | | - | + | + | | - | | - | B |
| Pinostrobin | C_16_H_14_O_4_ | 270.284 | 193 | | | 2670.2 | 2371 | | | + | + | | + | + | + | | + | | + | B |
| Pyrrolidine, 1-(m-methoxycinnamoyl) | C_14_H_17_NO_2_ | 231.295 | 161 | | | 2619.86 | 1918 | | | + | + | | + | + | + | | + | | + | B |
| Dihydroalatamide | C_16_H_17_NO_2_ | 255.317 | 134 | | | 2626.29 | 2255 | | | + | + | | + | + | + | | + | | + | B |
| 5,7-Dimethoxyflavanone | C_17_H_16_O_4_ | 284.311 | 134 | | | 3066.89 | 2529 | | | + | + | | + | + | + | | + | | + | B |
| Squalene | C_30_H_50_ | 410.730 | 69 | | | 3039.64 | 2914 | | | + | + | | + | + | + | | + | | + | B |
| Hispidin | C_13_H_10_O_5_ | 246.218 | 288 | | | 3079.91 | 2323 | | | + | + | | + | + | + | | + | | + | B |
| 5-Hydroxy-4',7-dimethoxyflavanone | C_17_H_16_O_5_ | 300.31 | 134 | | | 2937.06 | 2560 | | | + | + | | + | + | + | | + | | + | B |

**CRP, CNP, CWP, LR** denote the crown root peel, crown with no peel, crown with peel, and lateral roots of *Piper methysticum*, respectively. **SWP, SP** and **SNP** denote stems with peel, stem peels, and stems with no peels, respectively. Kovat’s retention indices for all identified putative metabolites are denoted as RI.

* Metabolite Identification confidence level (A-B)*: (A) Standards compounds used – level 1, and (B) Putatively identified - level 2 (e.g. these compounds were identified without use of standards by spectral similarity and chemical characteristics reported in public or commercially available libraries/databases and repositories.

**Supplementary Table S2:** Metabolites identified in common in various parts of *P.methysticum* by LC-MS analysis.

| **Compound name** | **Formula** | **Theoretical molecular weight** | **Observed m/z** | **Adducts** | **Mass error (ppm)** | **Retent-ion Time** | **CRP** | **CNP** | **CWP** | **LR** | **SWP** | **SP** | **SNP** | **Identification confidence level*** |
| --- | --- | --- | --- | --- | --- | --- | --- | --- | --- | --- | --- | --- | --- | --- |
| **Kava lactones** | | | | | | | | | | | | | | |
| 11-Hydroxy-12-methoxydihydrokawain | C_15_H_18_O_5_ | 278.304 | 277.110 | M-H | 9.91 | 2.94 | + | + | + | + | + | + | + | A |
| Desmethoxyyangonin | C_14_H_12_O_3_ | 228.247 | 209.058 | M-H_2_O-H | -9.00 | 3.58 | + | + | + | + | + | + | + | A |
|  |  |  | 457.167 | 2M+H | 7.03 | 4.34 | + | + | + | + | + | + | + | A |
| Dihydrokavain | C_14_H_16_O_3_ | 232.279 | 231.101 | M-H | -1.31 | 3.64 | + | + | + | + | + | + | + | A |
|  |  |  | 255.102 | M+Na | 14.31 | 3.49 | + | + | + | + | + | + | + | A |
|  |  |  | 233.118 | M+H | 7.74 | 3.21 | + | + | + | + | + | + | + | A |
| Dihydromethysticin | C_15_H_16_O_5_ | 276.288 | 275.091 | M-H | -0.50 | 3.48 | + | + | + | + | + | + | + | A |
|  |  |  | 553.203 | 2M+H | -4.94 | 3.247 | + | + | + | + | + | + | + | A |
| Methysticin | C_15_H_14_O_5_ | 274.272 | 273.077 | M-H | 3.88 | 3.48 | + | + | + | + | + | + | + | A |
|  |  |  | 275.092 | M+H | 4.24 | 2.94 | + | + | + | + | + | + | + | A |
|  |  |  | 297.077 | M+Na | 15.84 | 5.46 | + | + | + | + | + | + | + | A |
| p-Hydroxykavain | C_14_H_14_O_4_ | 246.262 | 267.065 | M+Na-2H | 3.41 | 3.64 | + | + | + | + | + | + | + | A |
|  |  |  | 269.081 | M+Na | 13.38 | 2.96 | + | + | + | + | + | + | + | A |
| Kavain | C_14_H_14_O_3_ | 230.263 | 211.075 | M-H_2_O-H | -1.29 | 2.69 | + | + | + | + | + | + | + | A |
|  |  |  | 231.102 | M+H | 5.74 | 3.05 | + | + | + | + | + | + | + | A |
| Dihydro-5,6-dehydrokawain | C_14_H_14_O_3_ | 230.263 | 211.075 | M-H_2_O-H | -3.70 | 3.62 | + | + | + | + | + | + | + | A |
|  |  |  | 231.105 | M+H | 17.97 | 3.30 | + | + | + | + | + | + | + | A |
|  |  |  | 253.086 | M+Na | 15.39 | 4.76 | + | + | + | + | + | + | + | A |
| p-Hydroxy-7,8-dihydrokavain | C_14_H_14_O_3_ | 230.263 | 229.085 | M-H | -3.77 | 3.58 | + | + | + | + | + | + | + | A |
| yangonin | C_15_H_14_O_4_ | 258.273 | 257.080 | M-H | -4.62 | 3.42 | + | + | + | + | + | + | + | A |
|  |  |  | 259.100 | M+H | 18.61 | 4.10 | + | + | + | + | + | + | + | A |
| 10-Methoxyyangonin | C_16_H_16_O_5_ | 288.299 | 269.084 | M-H_2_O-H | 9.90 | 4.18 | + | + | + | + | + | + | + | A |
|  |  |  | 289.111 | M+H | 15.37 | 4.42 | + | + | + | + | + | + | + | A |
| 11-Methoxyyangonin | C_16_H_16_O_5_ | 288.299 | 289.108 | M+H | 6.98 | 5.657 | + | + | + | + | + | + | + | A |
| 3a,4a-epoxy-5b-pipermethystine | C_16_H_17_NO_5_ | 303.314 | 304.115 | M+H | -6.11 | 3.53 | + | + | + | + | + | + | + | A |
| 5, 6, 7, 8-Tetrahydroyangonin | C_15_H_18_O_4_ | 262.305 | 263.131 | M+H | 17.35 | 8.77 | + | + | + | + | + | + | + | A |
| **Dihydrochalcones** | | | | | | | | | | | | | |  |
| Flavokawain A | C_18_H_18_O_5_ | 314.337 | 313.111 | M-H | 12.57 | 5.14 | + | + | + | + | + | + | + | A |
| Flavokawain B | C_17_H_16_O_4_ | 284.311 | 283.098 | M-H | 3.95 | 5.29 | + | + | + | + | + | + | + | A |
|  |  |  | 285.116 | M+H | 16.28 | 3.50 | + | + | + | + | + | + | + | A |
| Flavokawain C | C_17_H_16_O_5_ | 300.31 | 281.082 | M-H_2_O-H | 3.65 | 4.84 | + | + | + | + | + | + | + | A |
|  |  |  | 323.092 | M+Na | 11.86 | 3.33 | + | + | + | + | + | + | + | A |
| **Non-Kava lactones** | | | | | | | | | | | | | |  |
| (-)-bornyl ferulate | C_20_H_26_O_4_ | 330.424 | 329.179 | M-H | 14.57 | 5.82 | + | + | + | + | + | + | + | A |
|  |  |  | 353.177 | M+Na | 14.19 | 10.27 | + | + | + | + | + | + | + | A |
| (-)-bornyl-caffeate | C_19_H_24_O_4_ | 316.397 | 315.163 | M-H | 12.97 | 5.02 | + | + | + | + | + | + | + | A |
|  |  |  | 339.160 | M+Na | 15.08 | 10.13 | + | + | + | + | + | + | + | A |
| (-)-bornyl-p-coumarate | C_19_H_24_O_3_ | 300.398 | 299.164 | M-H | -0.02 | 7.27 | + | + | + | + | + | + | + | A |
| Bornyl cinnamate | C_19_H_24_O_2_ | 284.399 | 307.161 | M+Na | -17.90 | 3.51 | + | + | + | + | + | + | + | A |
| Mosloflavone | C_17_H_14_O_5_ | 298.294 | 281.082 | M-H_2_O-H | -1.02 | 3.39 | + | + | + | + | + | + | + | A |
| 1-Cinnamoyl  pyrrolidine | C_13_H_15_NO | 201.269 | 224.107 | M+Na | 15.52 | 5.46 | + | + | + | + | + | + | + | A |
| 3,4-methylene dioxy cinnamic acid | C_10_H_8_O_4_ | 192.170 | 191.034 | M-H | 1.43 | 1.89 | + | + | + | + | + | + | + | A |
|  |  |  | 193.049 | M+H | 2.40 | 8.50 | + | + | + | + | + | + | + | A |
| 9-Oxononanoic acid | C_9_H_16_O_3_ | 172.224 | 171.101 | M-H | -3.52 | 4.59 | + | + | + | + | + | + | + | A |
| Benzoic acid | C_7_H_6_O_2_ | 122.123 | 121.029 | M-H | -1.44 | 2.45 | + | + | + | + | + | + | + | A |
|  |  |  | 123.044 | M+H | 8.13 | 4.91 | + | + | + | + | + | + | + | A |
| Cinnamalacetone | C_12_H_12_O | 172.227 | 153.070 | M-H_2_O-H | -3.61 | 3.58 | + | + | + | + | + | + | + | A |
|  |  |  | 173.097 | M+H | 9.38 | 3.05 | + | + | + | + | + | + | + | A |
|  |  |  | 195.075 | M+Na | -13.56 | 4.83 | + | + | + | + | + | + | + | A |
| Cinnamic acid | C_9_H_8_O_2_ | 148.161 | 147.043 | M-H | -8.33 | 3.40 | + | + | + | + | + | + | + | A |
|  |  |  | 149.060 | M+H | 5.46 | 2.77 | + | + | + | + | + | + | + | A |
| Caproic acid | C_6_H_12_O_2_ | 116.16 | 137.060 | M+Na-2H | 17.55 | 3.31 | + | + | + | + | + | + | + | A |
| Glutathione | C_10_H_17_N_3_O_6_S | 307.321 | 306.079 | M-H | 11.56 | 3.46 | + | + | + | + | + | + | + | A |
|  |  |  | 308.093 | M+H | 9.43 | 3.64 | + | + | - | - | + | + | + | A |
| Phenylacetic acid | C_8_H_8_O_2_ | 136.15 | 135.044 | M-H | -4.27 | 3.35 | + | + | + | + | + | + | + | A |
|  |  |  | 137.060 | M+H | 5.27 | 3.83 | + | + | + | + | + | + | + | A |
| Pipermethystine | C_16_H_17_NO_4_ | 287.315 | 268.097 | M-H_2_O-H | 0.50 | 4.69 | + | + | + | + | + | + | + | A |
| Prenyl caffeate | C_14_H_16_O_4_ | 248.105 | 247.097 | M-H | -0.47 | 5.58 | + | + | + | + | + | + | + | A |
| Octadecadienoic acid, methyl ester | C_19_H_34_O_2_ | 294.479 | 317.247 | M+Na | 10.21 | 10.75 | + | + | + | + | + | + | + | A |
| 2,5,8-Trimethyl-1-Napthol | C_13_H_14_O | 186.254 | 187.114 | M+H | 16.46 | 2.98 | + | + | + | + | + | + | + | A |
| 5-methyl-1-phenylhexen-3-yn-5-ol | C_13_H_14_O | 186.254 | 209.092 | M+Na | -2.76 | 4.60 | + | + | + | + | + | + | + | A |

**CRP, CNP, CWP,** and **LR** denote the crown root peel, crown with no peel, crown with peel, and lateral roots of *Piper methysticum*. **SWP, SP** and **SNP** denote stems with peel, stem peels, and stems with no peels, respectively.

* Metabolite Identification confidence level (A)*: Putatively identified - level 2 identification (e.g. these compounds were identified without use of standards by spectral similarity and chemical characteristics reported in public or commercially available libraries/databases and repositories.

**Supplementary Table S3:** Secondary metabolites in various tissues of *P.methysticum* identified by LC/MS analysis.

| **Metabolites** | **CRP** | | **CNP** | | **CWP** | | **LR** | | **SP** | | **SNP** | | **SWP** | |
| --- | --- | --- | --- | --- | --- | --- | --- | --- | --- | --- | --- | --- | --- | --- |
|  | LC-MS Neg | LC-MS Pos | LC-MS Neg | LC-MS Pos | LC-MS Neg | LC-MS Pos | LC-MS Neg | LC-MS Pos | LC-MS Neg | LC-MS Pos | LC-MS Neg | LC-MS Pos | LC-MS Neg | LC-MS Pos |
| (-)-bornyl ferulate | +++ | +++ | +++ | +++ | +++ | +++ | +++ | +++ | +++ | +++ | +++ | +++ | +++ | +++ |
| (-)-bornyl-caffeate | +++ | +++ | +++ | +++ | +++ | +++ | +++ | +++ | +++ | +++ | +++ | +++ | +++ | +++ |
| (-)-bornyl-p-coumarate | +++ | --- | +++ | --- | +++ | --- | +++ | --- | +++ | --- | +++ | --- | +++ | --- |
| 1-Cinnamoylpyrrolidine | --- | +++ | --- | +++ | --- | +++ | --- | +++ | --- | +++ | --- | +++ | --- | +++ |
| 10-Methoxyyangonin | +++ | +++ | +++ | +++ | +++ | +++ | +++ | +++ | +++ | +++ | +++ | +++ | +++ | +++ |
| 11-Hydroxy-12-methoxydihydrokawain | +++ | --- | +++ | --- | +++ | --- | +++ | --- | +++ | --- | +++ | --- | +++ | --- |
| 11-Methoxyyangonin | --- | +++ | --- | +++ | --- | +++ | --- | +++ | --- | +++ | --- | +++ | --- | +++ |
| 2,5,8-Trimethyl-1-Napthol | --- | +++ | --- | +++ | --- | +++ | --- | +++ | --- | +++ | --- | +++ | --- | +++ |
| 3,4-methylene dioxy cinnamic acid | +++ | +++ | +++ | +++ | +++ | +++ | +++ | +++ | +++ | +++ | +++ | +++ | +++ | +++ |
| 3a,4a-epoxy-5b-pipermethystine | --- | +++ | --- | +++ | --- | +++ | --- | +++ | --- | +++ | --- | +++ | --- | +++ |
| 5-methyl-1-phenylhexen-3-yn-5-ol | --- | +++ | --- | +++ | --- | +++ | --- | +++ | --- | +++ | --- | +++ | --- | +++ |
| 5, 6, 7, 8-Tetrahydroyangonin | --- | +++ | --- | +++ | --- | +++ | --- | +++ | --- | +++ | --- | +++ | --- | +++ |
| 5,6,7,8-Tetrahydroyangonin^2^ | --- | +++ | --- | +++ | --- | +++ | --- | +++ | --- | +++ | --- | +++ | --- | +++ |
| 9-Oxononanoic acid | +++ | --- | +++ | --- | +++ | --- | +++ | --- | +++ | --- | +++ | --- | +++ | --- |
| Benzoic acid | +++ | +++ | +++ | +++ | +++ | +++ | +++ | +++ | +++ | +++ | +++ | +++ | +++ | +++ |
| Bornyl cinnamate | --- | +++ | --- | +++ | --- | +-+ | --- | +++ | --- | +++ | --- | +++ | --- | +++ |
| Caproic acid | +++ | --- | +++ | --- | +++ | --- | +++ | --- | +++ | --- | +++ | --- | +++ | --- |
| Cinnamalacetone | +++ | +++ | +++ | +++ | +++ | +++ | +++ | +++ | +++ | +++ | +++ | +++ | +++ | +++ |
| Cinnamalacetone^2^ | --- | +++ | --- | +++ | --- | +++ | --- | +++ | --- | +++ | --- | +++ | --- | +++ |
| Cinnamic acid | +++ | +++ | +++ | +++ | +++ | +++ | +++ | +++ | +++ | +++ | +++ | +++ | +++ | +++ |
| Desmethoxyyangonin | +++ | --- | +++ | --- | +++ | --- | +++ | --- | +++ | --- | +++ | --- | +++ | --- |
| Dihydro-5,6-dehydrokawain | +++ | +++ | +++ | +++ | +++ | +++ | +++ | +++ | +++ | +++ | +++ | +++ | +++ | +++ |
| Dihydro-5,6-dehydrokawain^2^ | --- | +++ | --- | +++ | --- | +++ | --- | +++ | --- | +++ | --- | +++ | --- | +++ |
| Dihydrokavain | +++ | +++ | +++ | +++ | +++ | +++ | +++ | +++ | +++ | +++ | +++ | +++ | +++ | +++ |
| Dihydrokavain2 | --- | +++ | --- | +++ | --- | +++ | --- | +++ | --- | +++ | --- | +++ | --- | +++ |
| Dihydromethysticin | +++ | --- | +++ | --- | +++ | --- | +++ | --- | +++ | --- | +++ | --- | +++ | --- |
| Flavokawain A | +++ | --- | +++ | --- | +++ | --- | +++ | --- | +++ | --- | +++ | --- | +++ | --- |
| Flavokawain B | +++ | +++ | +++ | +++ | +++ | +++ | +++ | +++ | +++ | +++ | +++ | +++ | +++ | +++ |
| Flavokawain C | +++ | +++ | +++ | +++ | +++ | +++ | +++ | +++ | +++ | +++ | +++ | +++ | +++ | +++ |
| Glutathione | +++ | ++- | +++ | --+ | +++ | --- | +++ | --- | +++ | +++ | +++ | -++ | +++ | +-- |
| Kavain | +++ | +++ | +++ | +++ | +++ | +++ | +++ | +++ | +++ | +++ | +++ | +++ | +++ | +++ |
| Methysticin | +++ | +++ | +++ | +++ | +++ | +++ | +++ | +++ | +++ | +++ | +++ | +++ | +++ | +++ |
| Methysticin^2^ | --- | +++ | --- | +++ | --- | +++ | --- | +++ | --- | +++ | --- | +++ | --- | +++ |
| Mosloflavone | +++ | --- | +++ | --- | +++ | --- | +++ | --- | +++ | --- | +++ | --- | +++ | --- |
| Octadecadienoic acid, methyl ester | --- | +++ | --- | +++ | --- | +++ | --- | +++ | --- | +++ | --- | +++ | --- | +++ |
| p-Hydroxy-7,8-dihydrokavain | +++ | --- | +++ | --- | +++ | --- | +++ | --- | +++ | --- | +++ | --- | +++ | --- |
| p-Hydroxykavain | +++ | +++ | +++ | +++ | +++ | +++ | +++ | +++ | +++ | +++ | +++ | +++ | +++ | +++ |
| Phenyl acetic acid | +++ | +++ | +++ | +++ | +++ | +++ | +++ | +++ | +++ | +++ | +++ | +++ | +++ | +++ |
| Pipermethystine | +++ | --- | +++ | --- | +++ | --- | +++ | --- | +++ | --- | +++ | --- | +++ | --- |
| Prenyl caffeate | +++ | --- | +++ | --- | +++ | --- | +++ | --- | +++ | --- | +++ | --- | +++ | --- |
| Yangonin | +++ | +++ | +++ | +++ | +++ | +++ | +++ | +++ | +++ | +++ | +++ | +++ | +++ | +++ |

**CRP, CNP, CWP, LR** denote the crown root peel, crown with no peel, crown with peel, and lateral roots of *Piper methysticum*, respectively. **SWP, SP** and **SNP** denote stems with peel, stem peels, and stems with no peels, respectively. Samples where the compounds are present are indicated by (+) and the compounds absent are indicated by (-). Since there were three samples of each tissue, each tissue should be counted three times. Compounds designated with superfix ^1^ and ^2^ with their chemical names, indicate different adducts detected in positive and negative mode of LC-MS analysis. Where ^1^ and ^2^  in positive mode denote [M+H] and [M+Na] adducts and for negative mode they denote [M-H] and [M-H2O-H] adducts, respectively. **LC-MS Neg** and **LC-MS pos** denote negative and positive mode of analysis.

**Supplementary Table S4: P-values of mixed linear model with GC/MS data, except SWP and CWP.**

| **Metabolites** | **Model** | **P-values** | **Adjusted p-values** |
| --- | --- | --- | --- |
| Tetrahydro-11-methoxyiangonin | tissue | 1.17808643951278E-08 | 3.35754635261143E-07 |
| Tetrahydro-11-methoxyiangonin | structural location | 1.12157338971741E-08 | 3.35754635261143E-07 |
| Kavain | structural location | 1.56824889741003E-07 | 2.97967290507906E-06 |
| Hedycaryol | structural location | 2.31909205616887E-07 | 3.30470618004064E-06 |
| Desmethoxyyangonin | structural location | 2.98560005607129E-07 | 3.40358406392127E-06 |
| Kavain | tissue | 8.71479257091856E-07 | 7.04964789007834E-06 |
| Dihydromethysticin | structural location | 9.5321358395406E-07 | 7.04964789007834E-06 |
| Pipermethystine | tissue | 1.17683208378239E-06 | 7.04964789007834E-06 |
| Flavokavain C | tissue | 1.23678033159269E-06 | 7.04964789007834E-06 |
| Pinostrobin | tissue | 1.23678033159269E-06 | 7.04964789007834E-06 |
| Flavokavain C | structural location | 1.71285673566945E-06 | 8.13606949442991E-06 |
| Pinostrobin | structural location | 1.71285673566945E-06 | 8.13606949442991E-06 |
| 5,7-Dimethoxyflavanone | tissue | 2.09936260164856E-06 | 9.20489756107445E-06 |
| Hedycaryol | tissue | 2.39861793005502E-06 | 9.76580157236689E-06 |
| Bornyl cinnamate | structural location | 2.65482663475147E-06 | 1.00883412120556E-05 |
| 5,7-Dimethoxyflavanone | structural location | 3.38463137583567E-06 | 1.13484699072137E-05 |
| Flavokavain B | tissue | 3.21104194600519E-06 | 1.13484699072137E-05 |
| 5-Hydroxy-4',7-dimethoxyflavanone | structural location | 5.24920882893909E-06 | 1.66224946249738E-05 |
| Hispidin | structural location | 6.93395120365814E-06 | 2.08018536109744E-05 |
| Flavokavain B | structural location | 8.48238314965499E-06 | 2.41747919765167E-05 |
| Yangonin | structural location | 1.07694103117506E-05 | 2.92312565604659E-05 |
| Pyrrolidine, 1-(m-methoxycinnamoyl)- | structural location | 1.13267332106926E-05 | 2.93465360458852E-05 |
| Bornyl cinnamate | tissue | 1.28322076620169E-05 | 3.18015581189114E-05 |
| Desmethoxyyangonin | tissue | 1.36651761903916E-05 | 3.24547934521802E-05 |
| Dihydromethysticin | tissue | 3.16880704616107E-05 | 7.22488006524724E-05 |
| Dihydro-5,6-dehydrokawain | structural location | 3.64760123721108E-05 | 7.99666425080892E-05 |
| Dihydro-5,6-dehydrokawain | tissue | 4.23606798339859E-05 | 8.81051458593296E-05 |
| Dihydroalatamide | tissue | 4.3279720773004E-05 | 8.81051458593296E-05 |
| 5-Hydroxy-4',7-dimethoxyflavanone | tissue | 0.000160794575246981 | 0.000316044509968204 |
| Hispidin | tissue | 0.000288700663445884 | 0.00054853126054718 |
| Pyrrolidine, 1-(m-methoxycinnamoyl)- | tissue | 0.000331809291186813 | 0.000607065082339764 |
| Yangonin | tissue | 0.000340808467278464 | 0.000607065082339764 |
| Benzenepropanal | structural location | 0.000768296646768696 | 0.00132705784441866 |
| Linoelaidic acid | structural location | 0.00192948737466425 | 0.00323472883399595 |
| Hydrocinnamic acid | structural location | 0.00257290031861235 | 0.00419015194745439 |
| 13-Tetradece-11-yn-1-ol | structural location | 0.00312603784219756 | 0.0049495599168128 |
| 13-Tetradece-11-yn-1-ol | tissue | 0.0051504638001858 | 0.00793449828677272 |
| Linoelaidic acid | tissue | 0.00535598442737761 | 0.00803397664106642 |
| Palmitic Acid | tissue | 0.00559118630532374 | 0.00817173383085777 |
| Benzenepropanal | tissue | 0.00613713798625426 | 0.00874542163041232 |
| Flavokawain A | tissue | 0.0100443689605078 | 0.0139641227011937 |
| Palmitic Acid | structural location | 0.0136342656997011 | 0.0185036463067371 |
| Hydrocinnamic acid | tissue | 0.0163771914601789 | 0.021709300307679 |
| δ-Cadinol | structural location | 0.0188348810048697 | 0.024399732210854 |
| δ-Cadinol | tissue | 0.0247886562895032 | 0.0313989646333707 |
| α-epi-7-epi-5-Eudesmol | structural location | 0.049881514927136 | 0.0618097032792772 |
| Squalene | tissue | 0.0680707001141239 | 0.08255382779798 |
| Pipermethystine | structural location | 0.0772657956864828 | 0.0917531323776984 |
| (E)-N-Cinnamoylpiperidine | structural location | 0.108622680203535 | 0.126356995338806 |
| Flavokawain A | structural location | 0.113847191950331 | 0.129785798823377 |
| α-epi-7-epi-5-Eudesmol | tissue | 0.151377706423306 | 0.169186848355459 |
| Dihydrokavain | structural location | 0.160558492282797 | 0.175996808848451 |
| (E)-N-Cinnamoylpiperidine | tissue | 0.248113295536212 | 0.266838827274794 |
| Dihydrokavain | tissue | 0.268237883548967 | 0.283139988190577 |
| Dihydroalatamide | structural location | 0.309904174467242 | 0.321173417175142 |
| Squalene | structural location | 0.630662469101325 | 0.641924298906705 |
| 1,10-Undecadiene | tissue | 0.71947465649012 | 0.71947465649012 |

**Supplementary Table S5. 3D morphological and geometric descriptors of roots of *P. methysticum***

| **Parameters** | **CR-1** | **CR-2** | **LR- 1** | **LR-2** | **Fold difference** |
| --- | --- | --- | --- | --- | --- |
| Porosity (%) | 11.8392 | 3.34639 | 13.5404 | 0.965264 | 1.04687 |
| Void shape factor | 5.22242 | 16.3949 | 2.73936 | 2.60712 | 4.04328 |
| Feret shape 3D_max_ | 4.66 | 4.31 | 2.26 | 3.29 | 1.61621 |
| Spheriphicity of pores | 1.13887 | 0.941099 | 1.24854 | 1.2478 | 0.83320 |
| Anisotropy | 0.658634 | 0.995278 | 0.603178 | 0.568136 | 1.41201 |
| Volume of segmented regions (μm^3^) | | | | |  |
| Whole root | 1.18E+13 | 3.39E+12 | 4.96E+11 | 1.86E+11 | 2.23E+01 |
| Intercellular air spaces | 2.19E+11 | 7.75E+10 | 9.07E+10 | 6.71E+10 | 1.88E+00 |
| Cork | 3.72E+11 | 2.43E+10 | 1.40E+11 | 7.18E+10 | 1.87E+00 |

**CR-1** and **CR-2** denote crown root samples, and **LR-1** and **LR-2** denote lateral root samples.

**
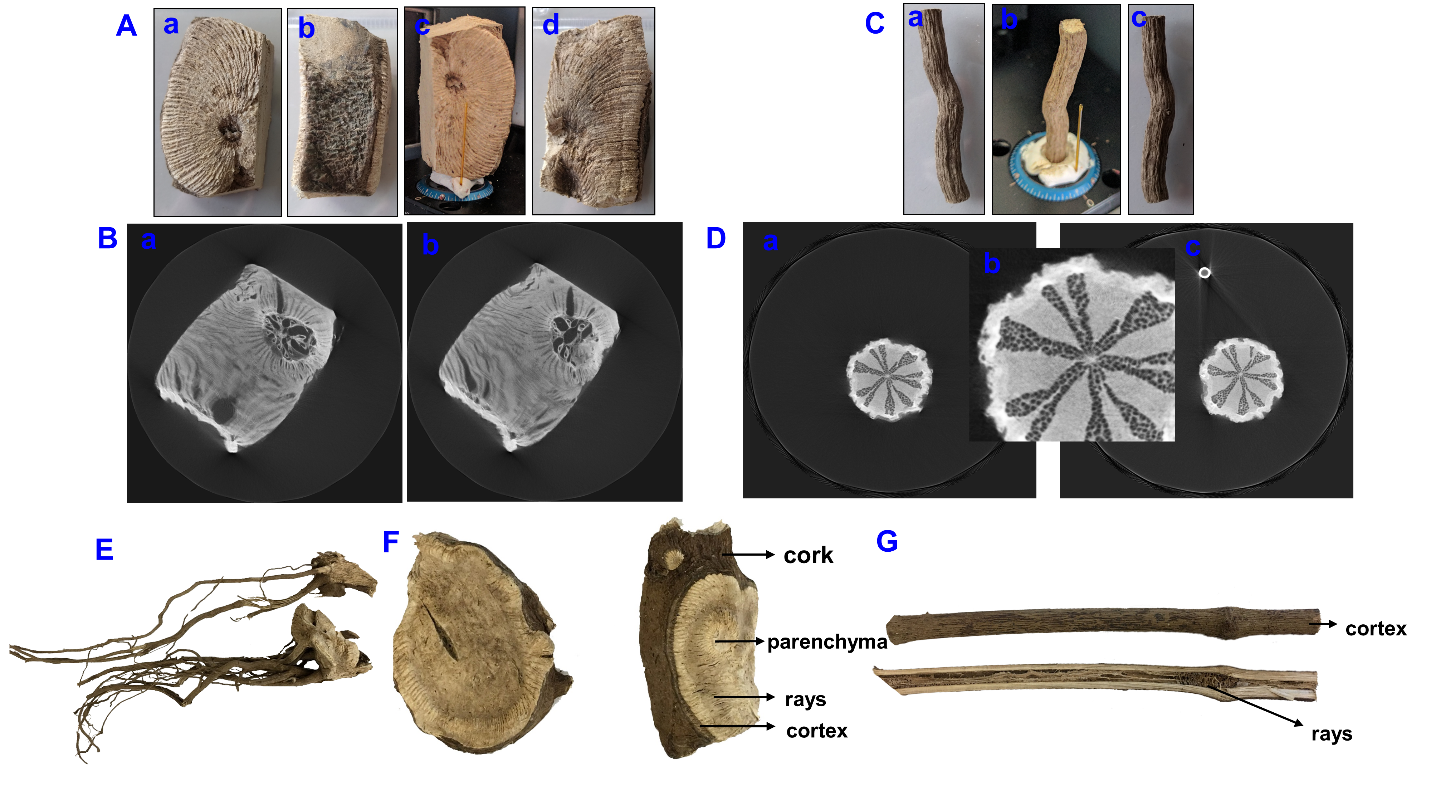
****Supplementary Figure S1. Pictorial representations of plant parts of *P.methysticum* and their μCT imaging and sectional views. A:** Images of the crown root sections from different views **(a)** front view indicating the cork, cortex and parenchyma **(b)** side view indicating the cork **(c)** view of the root section mounted for scanning **(d)** back view of the root section, **B:** Images (a) and (b) both represent μCT grayscale image slices (view along rotation axis). The isotropic nominal resolution was 38.1 µm/pixel, **C:** Images of the lateral root sections from different views (a) and (c) indicate side views of the root (b) view of the root section mounted for vertically for scanning, **D:** Images **(a)** and **(c)** both represent μCT grayscale image slices (view along rotation axis), **(b)** shows a zoomed view of the inner root tissue structure. The isotropic nominal resolution was 17.2 µm/pixel, **E:** Lateral roots, **F:** Crown root transverse view, and **G:** Stems longitudinal section.


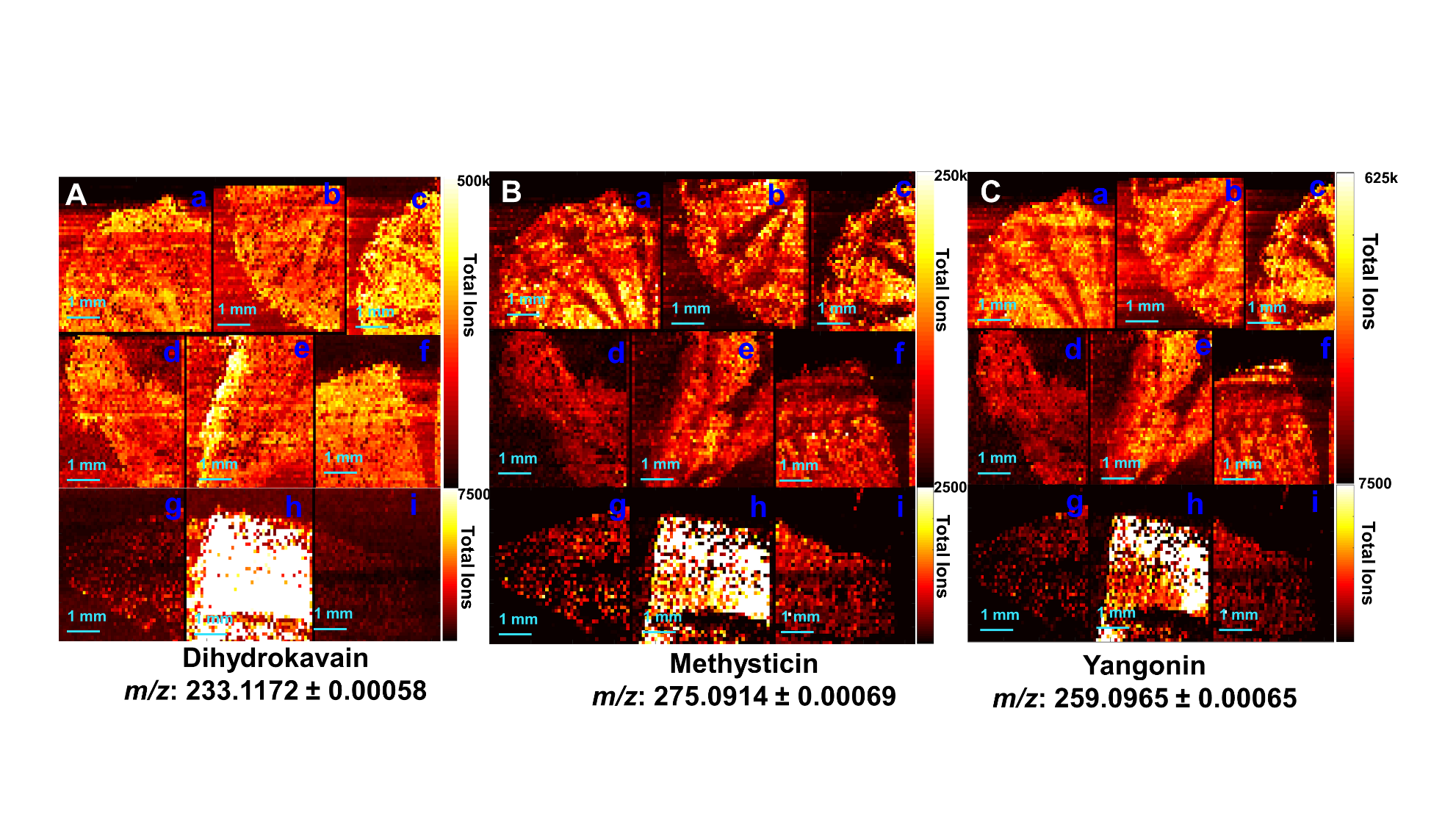


**Supplementary Figure S2. IR-MALDESI ion abundance heatmaps for various constituents in different parts of *P. methysticum****.* **(A)** Distribution of dihydrokavain (*m/z*: 233.1172), **(B)** Distribution of methysticin (*m/z*: 275.0914) **(C)** Distribution of yangonin (*m/z*: 259.0965). Images in **a-c**, **d-f** and **g-i**, indicate lateral roots, crown roots and stem sample images, respectively.


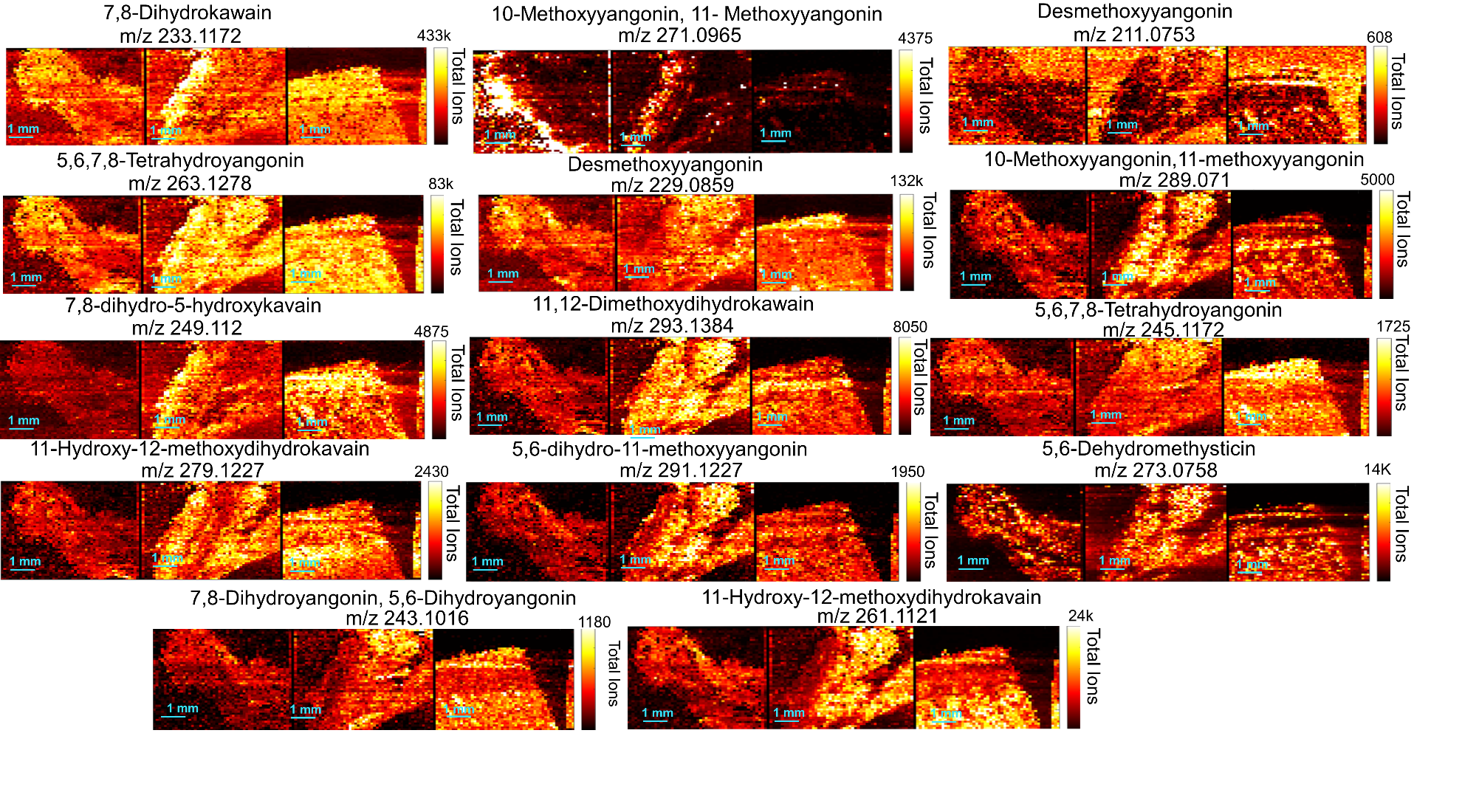


**Supplementary Figure S3.1: IR-MALDESI ion abundance heatmaps for various constituents in crown roots of *P. methysticum***

**
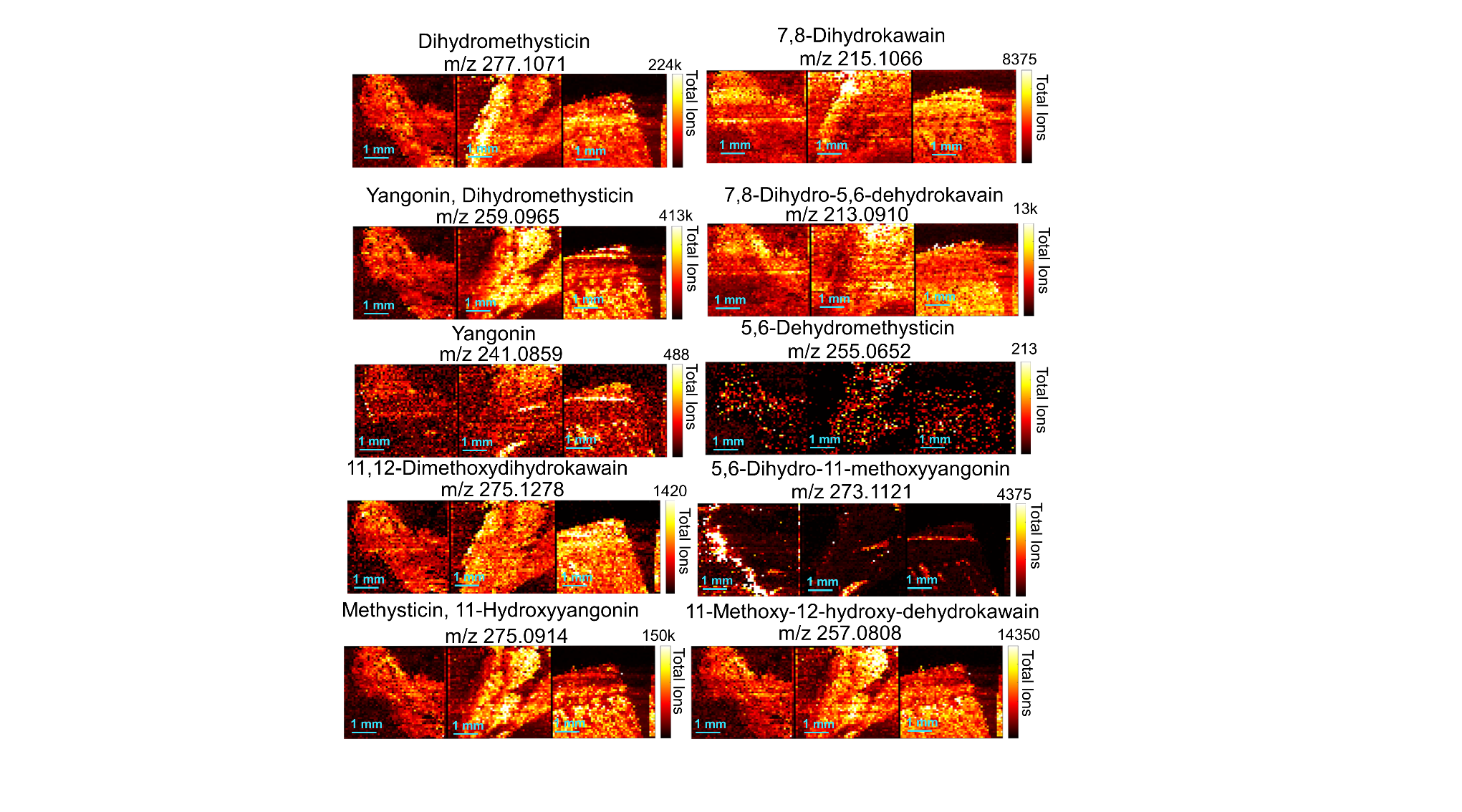
**

**Supplementary Figure S3.2: IR-MALDESI ion abundance heatmaps of some constituents identified in crown roots of *P. methysticum***


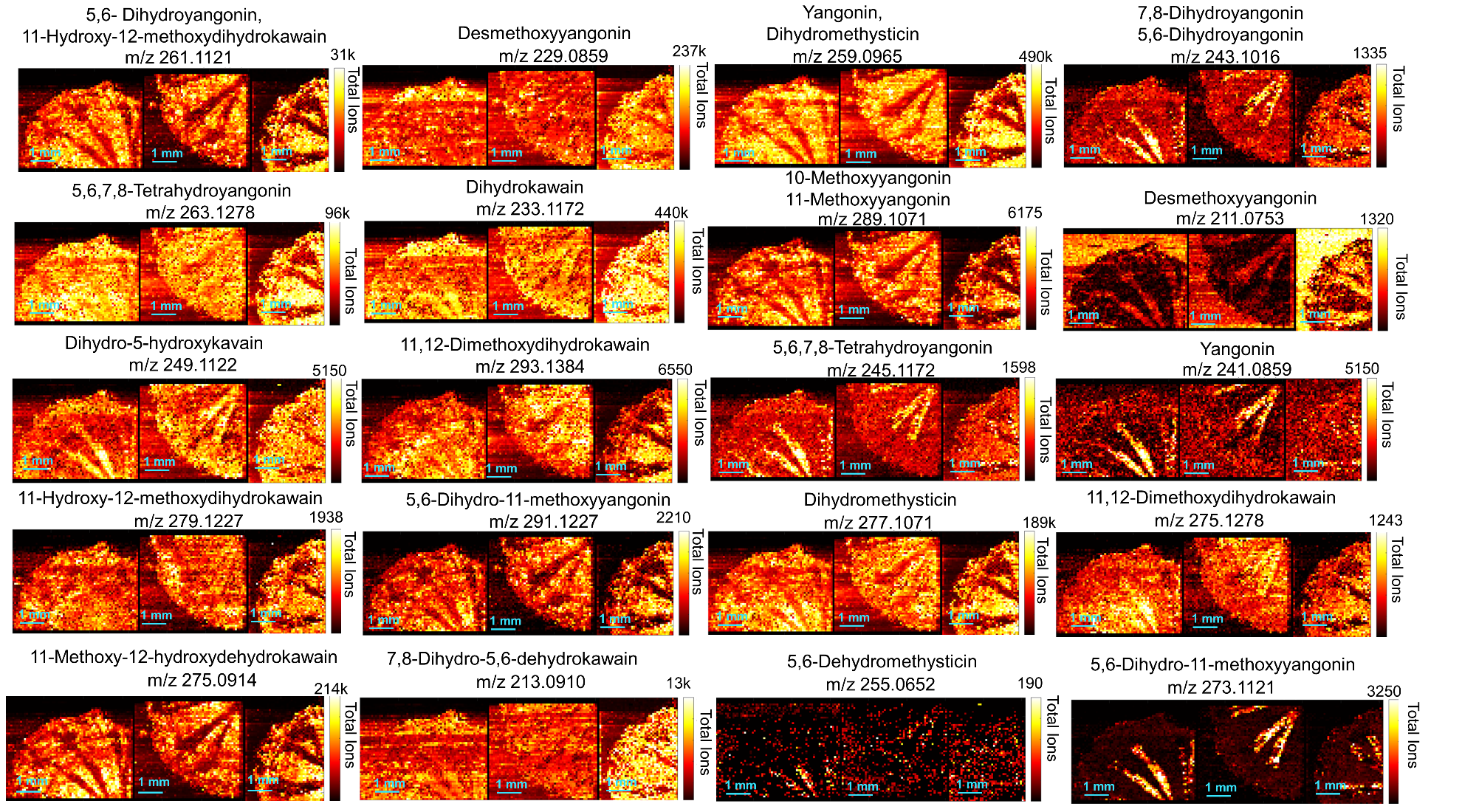


**Fig. S4 : IR-MALDESI ion abundance heatmaps for various constituents in lateral roots of *P. methysticum***

**
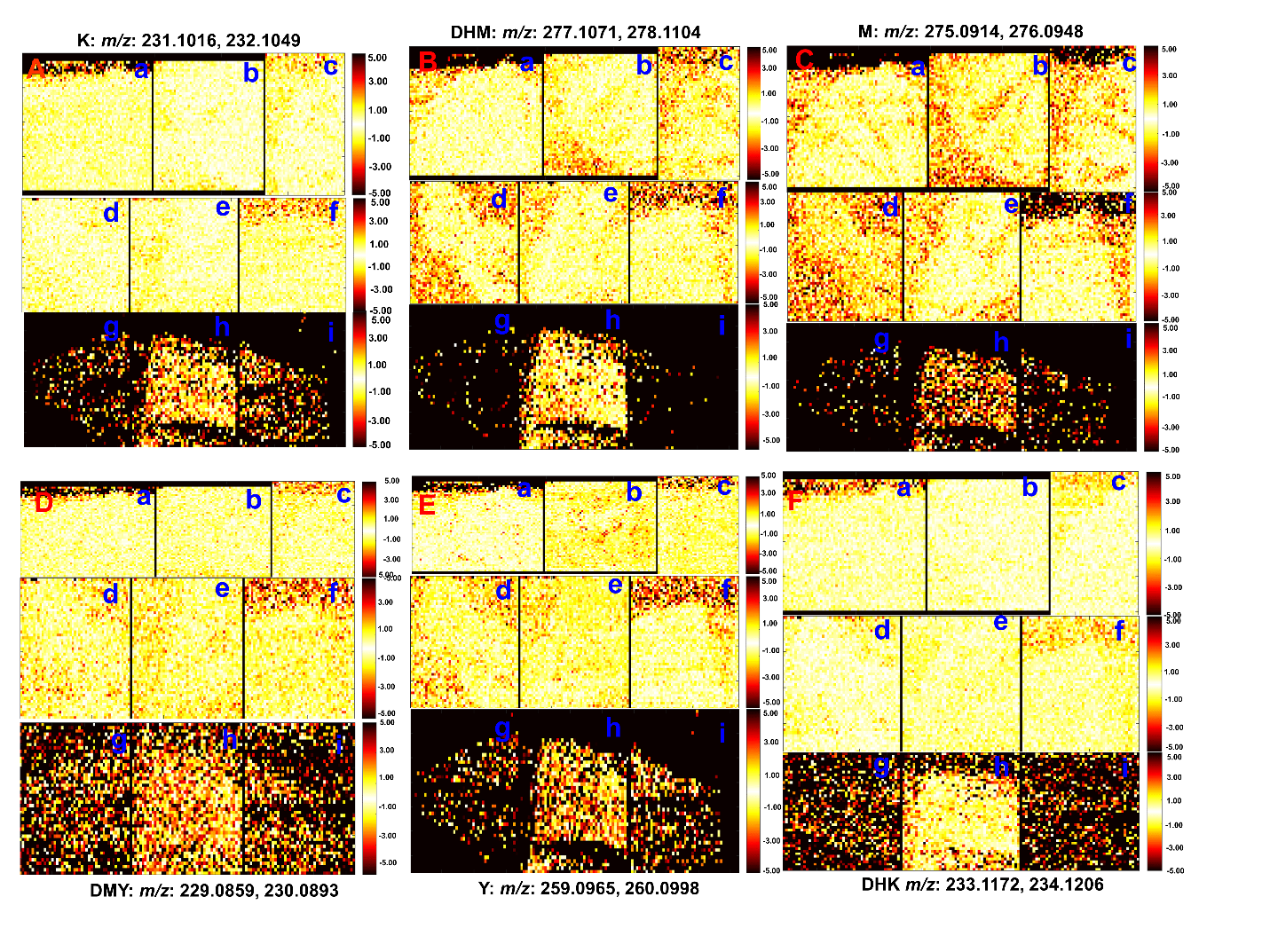
**

**Supplementary Figure S5. Isotope Count Heatmap for kavalactones on various tissues of *P. methysticum*. (A-F)** represent heatmaps for kavain (K, *m/z* = 231.1016), dihydromethysticin (DHM, *m/z* = 277.1071), methysticin (M, m/z = 275.0914), desmethoxyyangonin (DMY, *m/z* = 229.0859), yangonin (Y, *m/z* = 259.0965), and dihydrokavain (DHK, m/z = 233.1172), respectively. Images in **a-c**, **d-f** and **g-i**, indicate lateral roots, crown roots and stem sample images, respectively.
