# Supplementary material for: 3D Imaging and metabolomic profiling reveal higher neuroactive kavalactone contents in lateral roots and crown root peels of Piper methysticum (kava)
Source: Gigascience. 2020 Sep 22;9(9):giaa096. doi: 10.1093/gigascience/giaa096 (PMC7507772; doi:10.1093/gigascience/giaa096)
Supplement: giaa096_Response_to_Reviewer_Comments_Original_Submission [file giaa096_response_to_reviewer_comments_original_submission.pdf]

Author's Response To Reviewer Comments

Close

Response letter for Manuscript ref. no: GIGA-D-20-00163

The authors sincerely appreciate the time and effort invested by the reviewers in providing their valuable inputs. These suggestions have improved our manuscript significantly.
In the revised manuscript, all the comments made by the reviewers have been suitably addressed.
Specifically, (i) Figures and figure legends have been corrected (ii) tables have been modified, and (iii) certain references have been deleted, as recommended by the reviewers.
A detailed point-to-point response for each of reviewers' comments and indications of following it, are detailed below. We hope that the reviewers will find the revised manuscript satisfactory for further evaluation.

Queries/critiques are numbered and in blue Times Roman font.
Responses follow in black Times Roman.
Revised text from the manuscript is indicated in red Times Roman.

Reviewer reports:
Reviewer #1: The authors addressed most of my concerns. However, I found some issues in the revised manuscript that should be fixed prior to publication.

1. The authors added additional references to introduction, but the referencing is quite confusing. Specifically, this sentence: "Novel dimeric kavalactones, namley diyangonins (A-C) have also reported to be isolated from kava roots [10-15]." References 10-13 have nothing to do with dimeric kavalactones.
Response: The authors thank the reviewer for pointing out this error. As per the recommendations, the non-relevant references have been deleted from the specified sentence.

Section: Manuscript - Introduction
Please refer: Line numbers 54-55, page 3 - Reference nos. 10, 11
Text: "Novel dimeric kavalactones, ………….. to be isolated from kava roots [10, 11]"

2. The authors claim they deposited their raw data into the Metabolights repository, but no such information (or deposition ID) is mentioned in the "Availability of Data and Materials" section.
Response: As per the reviewer's suggestion, the Metabolights repository/deposition ID is mentioned in the "Availability of supporting data and materials" section.
Section: Manuscript: Availability of supporting data and materials
Please refer: Line numbers 464-466, page 22
Text: "Metabolomics data have been deposited ………..with the identifier MTBLS1485".

3. Fig.5: The mass of 7,8-dihydro-5-hydroxykavain (5-DHK) ion is not 231.1016 m/z. Furthermore, p-hydroxykavain is a known metabolite of kavain in liver (doi:10.1016/S1570-0232(03)00046-1), but has it ever been reported in the plant?

Response: We thank the reviewer for pointing out these important errors. The necessary modifications have been carried out in Fig.5, and these two compounds have been removed from the figure.
Please refer: Figure 5 legend, Page 31, Line nos: 685-689
Text: Legend for Figure 5 – "IR-MALDESI ion abundance …….. (A) Distribution of kavain (K), 7,8-Dihydro-5,6-dehydrokawain (5,6-DDK): m/z 231.1016".

4. I do not understand Figure 6 at all. The legend says it contains MS/MS spectra, but it clearly doesn't contain any spectra (mass spectrum = m/z vs intensity). There is no label or scale for X axis! In their response the authors explained that "The yaxis is abundance of the precursor (top) and MS/MS fragments (bottom) of the kavain m/z (231.1016)." but they didn't add this description into the legend. I am missing clear description how the MS/MS experiments were performed (precursor selection, collision energy?).

Response: We thank the reviewer for pointing out this important error.
Our apologies for the confusion. During revisions we meant to change 'spectra' into 'chromatograms'. The X-axis in (A) is an arbitrary length of time that the standards were analyzed. In (C) and (D) the time scale is just from the beginning of the experiment to the end across tissue.
In Parallel Reaction Monitoring (PRM) mode in Orbitrap-based Thermo MS, every nth scan is dedicated to a full MS scan across the entire m/z range of interest, or one or another of select m/z of interest. In this particular case the 1st scan was a full MS scan from 120-480 m/z, the next was an MS/MS scan of 231.1016 with an isolation window of 1.5 m/z fragmented with NCE (normalized collision energy) = 30, the 3rd was an MS/MS scan of 277.1071 with NCE = 20, and the 4th was an MS/MS scan of 229.0859 with NCE = 35, then the cycle repeated. Each NCE value was optimized in unpublished experiments. We have attached the updated figure to make this more clear.
As per the reviewer's suggestions, the requested description is now added in the manuscript's "Materials and Methods" section and the figure legend is now modified.
Please refer: Page 20, Line nos: 416-422 and Figure 6.

Text: In Parallel Reaction Monitoring (PRM) mode in ……………………was optimized in unpublished experiments (Figure. 6).

5. (a) Supplementary figures S2 to S5 need more careful preparation. The legend of Fig. S2 points to images a-c, d-f, and g-i,which are not indicated in the figure.
(b) It is unclear why Fig S2 shows 3 kavalactones, Fig. S3.1 shows 15 kavalactones, Fig.S3.2 shows 10 kavalactones, and Fig. S4 shows 20 kavalactones.
(c) Dihydrokawain is labeled as 233.1172 m/z in Fig. S3.1 but 215.1066 m/z in Fig. S3.2, why? (d)The inclusion of p-hydroxykavain is questionable (see above).
(e) Fig. S5 suffers again from the superposition of some signals (231.1016 m/z is not only kavain).

Responses: We thank the reviewer for queries. Following are the specific responses:
(a) We apologize for the oversights in Supplementary Figure S2 and the confusion they have caused. The legend and figure of Supplementary Figure S2 have been revised, as per the reviewer's sugegstions. Please refer: Additional files: Page no-53-56, Supplementary Figure S2.
(b) The focus of the study has been much more oriented towards the 3 kavalactones shown in Figure S2, hence its own figure here. Figure S3.1 and S3.2 go together but were split up for clearer presentation as a Figure with them 5 sub-figure wide would make the images too small. Their purposes were to show that we analyzed more kavalactones than just the ones of focus shown in the main bodies and figures of the paper in the crown roots, and then S4 has the same purpose, but for lateral roots. Supplementary Figures S3.1 and S3.2 show constituents from crown roots and Supplementary Figure S4 shows constituents from lateral roots.
(c) Dihydrokawain was identified both times but the 233.1172 m/z was its proton adduct peak, and the 215.1066 is the proton adduct with water loss. The water loss proton adduct is a commonly identifiable peak in many of our studies.
(d) As suggested by the reviewers, all occurrences of p-hydroxykavain in figures and text have been deleted.
(e) Yes, not all the images we provide in the supplementary images are as high quality as others. Since we had performed MS/MS on kava roots and stems and compared it to the standard, the identification of 231.1016 m/z as kavain is a solid one. For other kava lactones putatively identified without MS/MS, we have to rely on a combination of biological and spectral verification. Supplementary Figures S2-S4 have been suitably revised.

6. The methods section needs a clear explanation how the metabolites in Sup. Tables S1 and S2 were identified, and confidence of the identification should be noted in the tables, see Blaženović, I., Kind, T., Ji, J. & Fiehn, O. Software Tools and Approaches for Compound Identification of LC-MS/MS Data in Metabolomics. Metabolites 8, 31 (2018).
Response: A clear explanation of how metabolites were identified in Table S1 and S2 is provided in the methods section. The confidence of identification are now mentioned in Tables S1 and S2.
(a) Please refer: Manuscript: Methodology, Page 16, Line nos. 327-332
Text: "Baseline smoothing, peak picking, automated ……. were used for metabolite annotation."

(b) Please refer: Manuscript: Methodology, Page 15, Line nos. 302-306
Text: "The resulting data …………..specific molecular ions and masses".

(c) Please refer: Supplementary Tables S1 and S2 (Additional files: page 35-41).

We thank the reviewer once again for their efforts in providing us these important suggestions.

Reviewer #2: The authors have made substantial changes in the manuscript and have addressed all the issues that were raised for the previous version. The manuscript now reads well and there are no further comments or questions for the latest version.

Responses: The authors thank the reviewer for considering out manuscript favourably, and for the time and effort in providing us valuable suggestions to improve it.

Reviewer #3: I am satisfied how authors addressed all my questions with enough supporting data.
I have one minor comment that can significantly improve citation of your manuscript:

1. Please provide more detailed information on sectioning of kava roots. What was duration of soaking? Have you used some polymeric material to embed roots? There is a big challenge to section roots of woody plants without disturbing their morphology (without chemical fixation) and your optical images shows that you achieved that!

Response: We sincerely thank the reviewer for a positive approval of our manuscript and appreciating our work.
As per your recommendations, we have provided the requested information in the manuscript.

Please refer: Manuscript: Methods, Page: 18, Line nos. 364-371
Text: "The samples for cryo-sectioning were prepared ……..a polymeric gel upon freezing [43]".

We thank the reviewer once again, for the efforts in providing us constructive suggestions and helping us improve our manuscript further.

Close
