# Supplementary material for: 3D Imaging and metabolomic profiling reveal higher neuroactive kavalactone contents in lateral roots and crown root peels of Piper methysticum (kava)
Source: Gigascience. 2020 Sep 22;9(9):giaa096. doi: 10.1093/gigascience/giaa096 (PMC7507772; doi:10.1093/gigascience/giaa096)
Supplement: giaa096_Reviewer_1_Report_Original_Submission — Tomas Pluskal -- 7/6/2020 Reviewed [file giaa096_reviewer_1_report_original_submission.pdf]

Reviewer Report

Title: 3D imaging and metabolomic profiling reveal higher neuroactive kavalactone contents in lateral roots and crown root peels of Piper methysticum (Kava)

Version: Original Submission **Date:** 7/6/2020

Reviewer name: Tomas Pluskal

Reviewer Comments to Author:

The authors addressed most of my concerns. However, I found some issues in the revised manuscript that should be fixed prior to publication.

1. The authors added additional references to introduction, but the referencing is quite confusing. Specifically this sentence: "Novel dimeric kavalactones, namley diyangonins (A-C) have also reported to be isolated from kava roots [10-15]." References 10-13 have nothing to do with dimeric kavalactones.
2. The authors claim they deposited their raw data into the Metabolights repository, but no such information (or deposition ID) is mentioned in the "Availability of Data and Materials" section.
3. Fig.5: The mass of 7,8-dihydro-5-hydroxykavain (5-DHK) ion is not 231.1016 m/z. Furthermore, p-hydroxykavain is a known metabolite of kavain in liver (doi:10.1016/S1570-0232(03)00046-1), but has it ever been reported in the plant?
4. I do not understand Figure 6 at all. The legend says it contains MS/MS spectra, but it clearly doesn't contain any spectra (mass spectrum = m/z vs intensity). There is no label or scale for X axis! In their response the authors explained that "The y-axis is abundance of the precursor (top) and MS/MS fragments (bottom) of the kavain m/z (231.1016)." but they didn't add this description into the legend. I am missing clear description how the MS/MS experiments were performed (precursor selection, collision energy?).
5. Supplementary figures S2 to S5 need more careful preparation. The legend of Fig. S2 points to images a-c, d-f, and g-i, which are not indicated in the figure. It is unclear why Fig S2 shows 3 kavalactones, Fig. S3.1 shows 15 kavalactones, Fig. S3.2 shows 10 kavalactones, and Fig. S4 shows 20 kavalactones. Dihydrokawain is labeled as 233.1172 m/z in Fig. S3.1 but 215.1066 m/z in Fig. S3.2, why? The inclusion of p-hydroxykavain is questionable (see above). Fig. S5 suffers again from the superposition of some signals (231.1016 m/z is not only kavain).
6. The methods section needs a clear explanation how the metabolites in Sup. Tables S1 and S2 were identified, and confidence of the identification should be noted in the tables, see BlaÅ¾enoviÄ‡, I., Kind, T., Ji, J. & Fiehn, O. Software Tools and Approaches for Compound Identification of LC-MS/MS Data in Metabolomics. Metabolites 8, 31 (2018).

Methods

Are the methods appropriate to the aims of the study, are they well described, and are necessary controls included? Choose an item.

Conclusions

Are the conclusions adequately supported by the data shown? Choose an item.

Reporting Standards

Does the manuscript adhere to the journal's guidelines on minimum standards of reporting? Choose an item.

Choose an item.

Statistics

Are you able to assess all statistics in the manuscript, including the appropriateness of statistical tests used? Choose an item.

Quality of Written English

Please indicate the quality of language in the manuscript: Choose an item.

Declaration of Competing Interests

Please complete a declaration of competing interests, considering the following questions:

- Have you in the past five years received reimbursements, fees, funding, or salary from an organisation that may in any way gain or lose financially from the publication of this manuscript, either now or in the future?
- Do you hold any stocks or shares in an organisation that may in any way gain or lose financially from the publication of this manuscript, either now or in the future?
- Do you hold or are you currently applying for any patents relating to the content of the manuscript?
- Have you received reimbursements, fees, funding, or salary from an organization that holds or has applied for patents relating to the content of the manuscript?
- Do you have any other financial competing interests?
- Do you have any non-financial competing interests in relation to this paper?

If you can answer no to all of the above, write 'I declare that I have no competing interests' below. If your reply is yes to any, please give details below.

I declare that I have no competing interests

I agree to the open peer review policy of the journal. I understand that my name will be included on my report to the authors and, if the manuscript is accepted for publication, my named report including any attachments I upload will be posted on the website along with the authors' responses. I agree for my

report to be made available under an Open Access Creative Commons CC-BY license (http://creativecommons.org/licenses/by/4.0/). I understand that any comments which I do not wish to be included in my named report can be included as confidential comments to the editors, which will not be published.

Choose an item.

To further support our reviewers, we have joined with Publons, where you can gain additional credit to further highlight your hard work (see: https://publons.com/journal/530/gigascience). On publication of this paper, your review will be automatically added to Publons, you can then choose whether or not to claim your Publons credit. I understand this statement.

Yes Choose an item.
